# Supplementary material for: Long-range movement of large mechanically interlocked DNA nanostructures
Source: Nat Commun. 2016 Aug 5;7:12414. doi: 10.1038/ncomms12414 (PMC4980458; doi:10.1038/ncomms12414)
Supplement: Supplementary Information — Supplementary Figures 1-31, Supplementary Tables 1-2 and Supplementary Methods [file ncomms12414-s1.pdf]

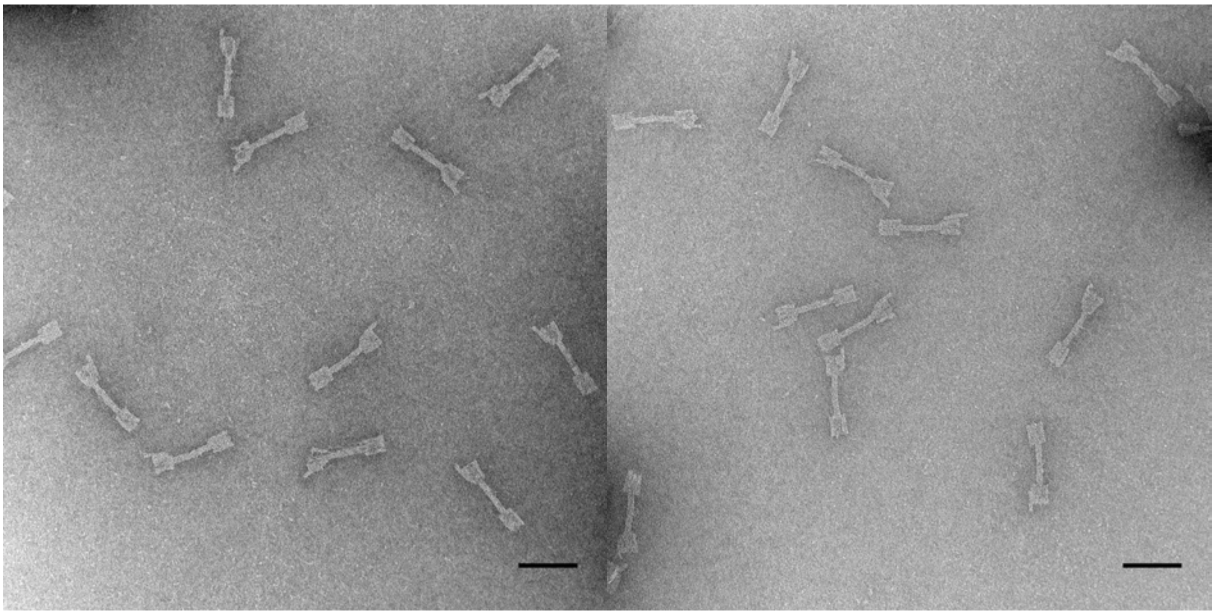

Supplementary Figure 1 l Negative stain TEM images of dumbbell D1. Scale bars: 100 nm.

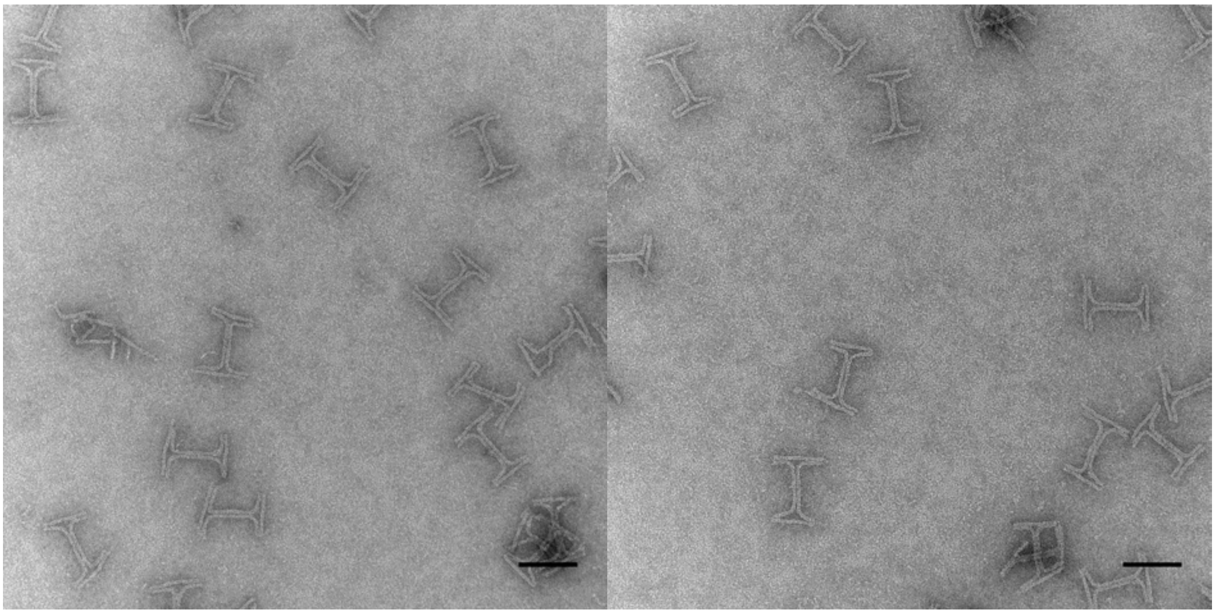

Supplementary Figure 2 l Negative stain TEM images of dumbbell D2. Scale bars: 100 nm.

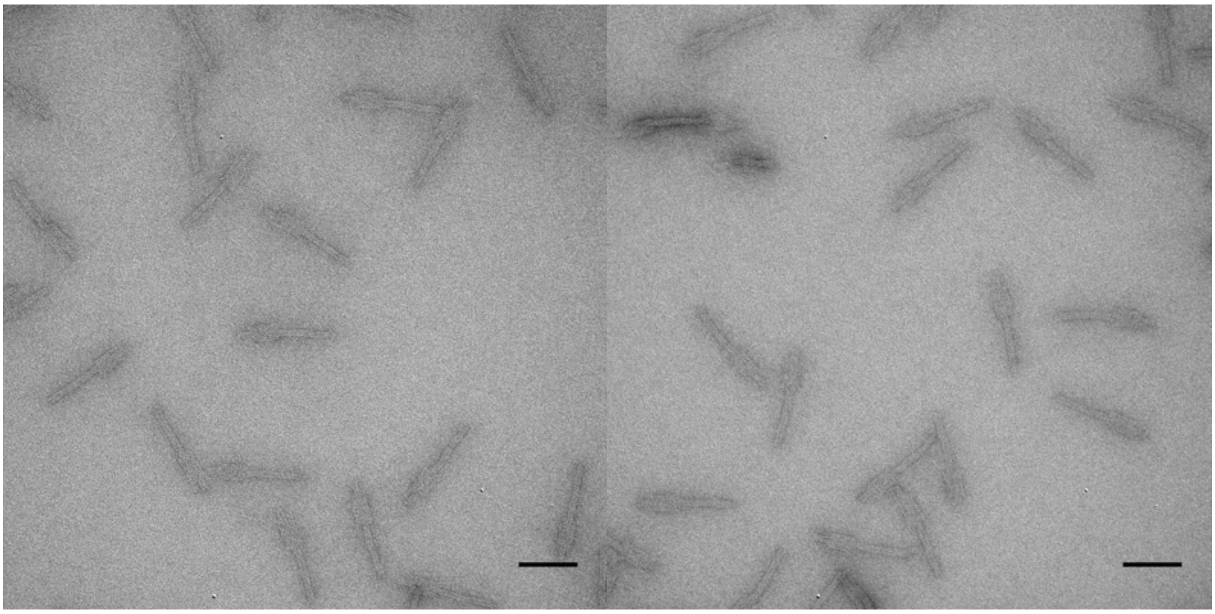

Supplementary Figure 3 l Negative stain TEM images of stopper modules.

Scale bars: 100 nm.

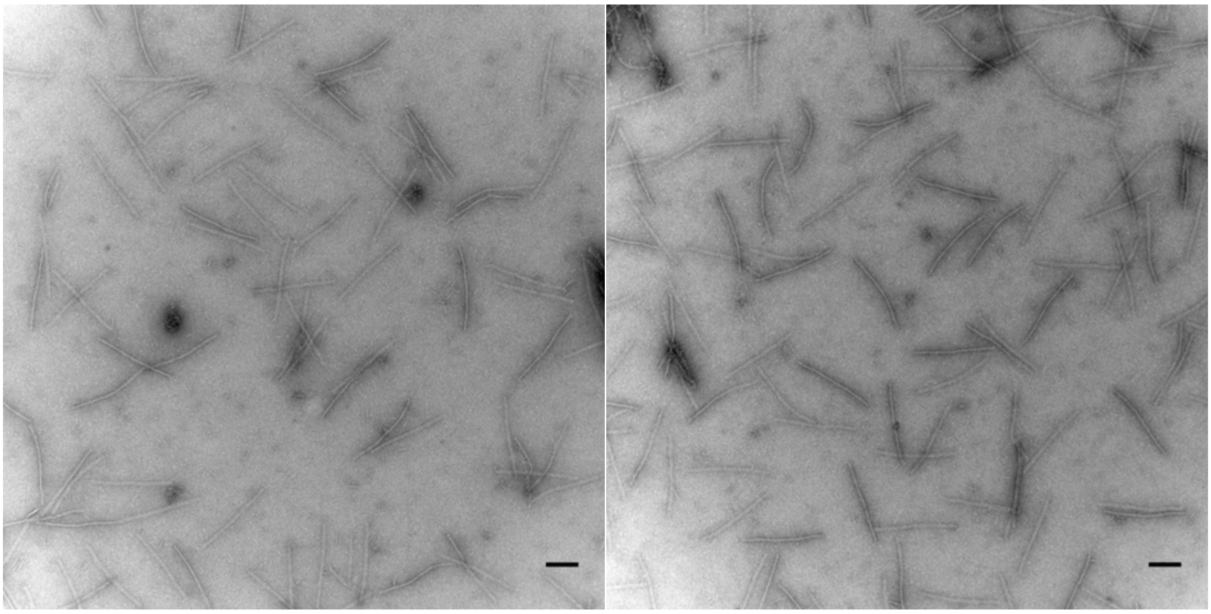

Supplementary Figure 4 l Negative stain TEM images of the axle module. Scale bars: 100 nm.

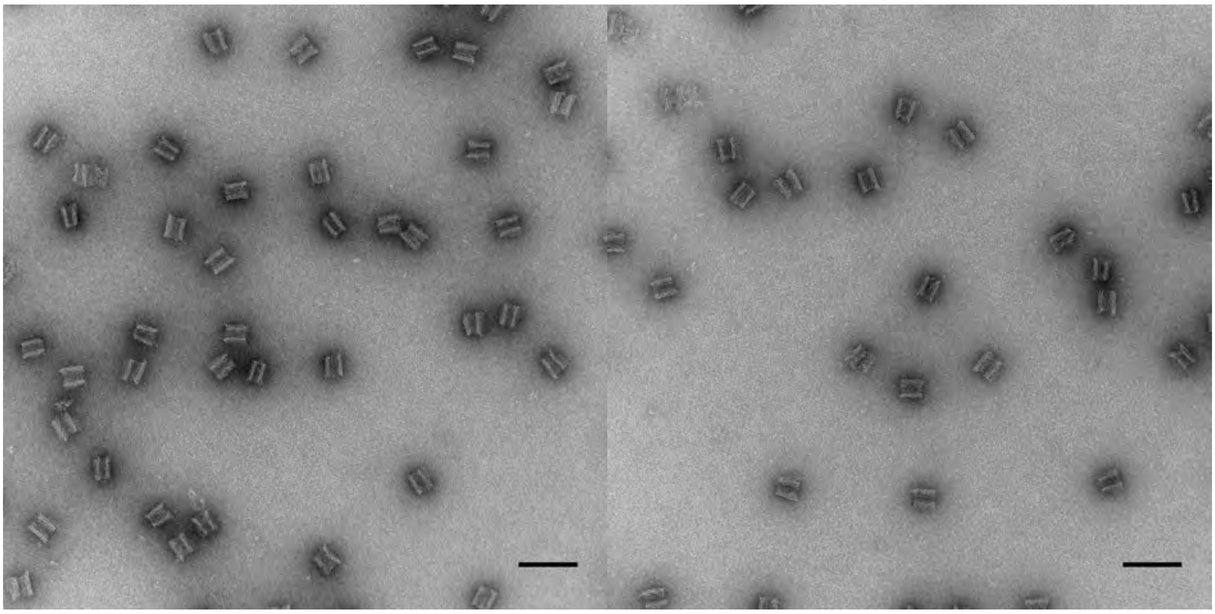

Supplementary Figure 5 l Negative stain TEM images of R1 in the closed state.

Scale bars: 100 nm.

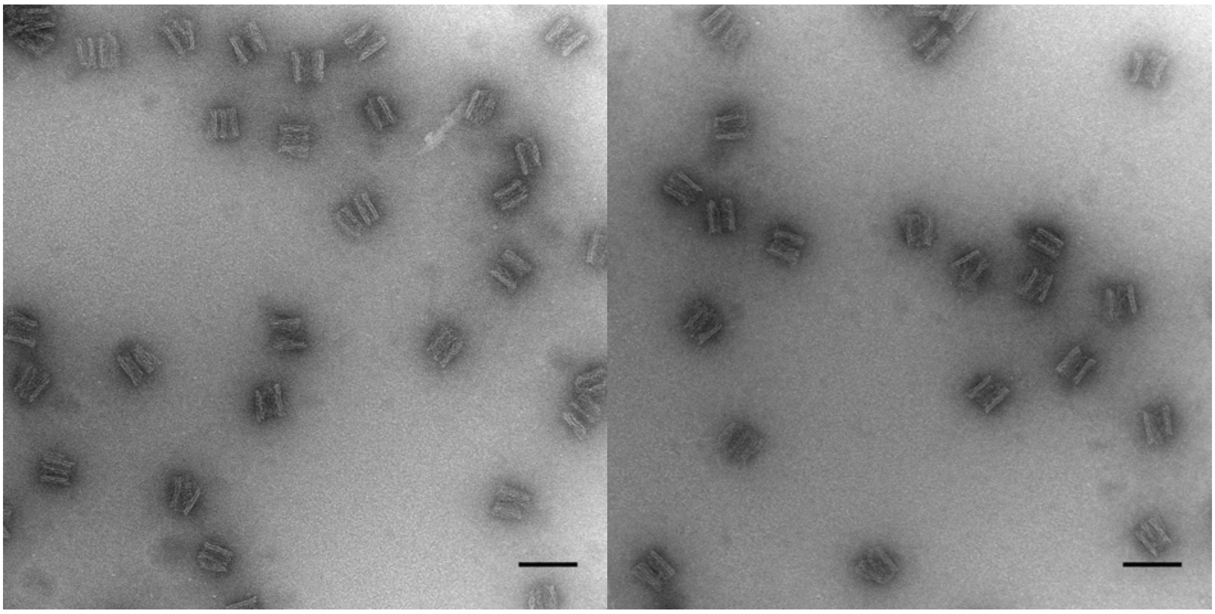

Supplementary Figure 6 l Negative stain TEM images of R1 in the open configuration. Scale bars: 100 nm.

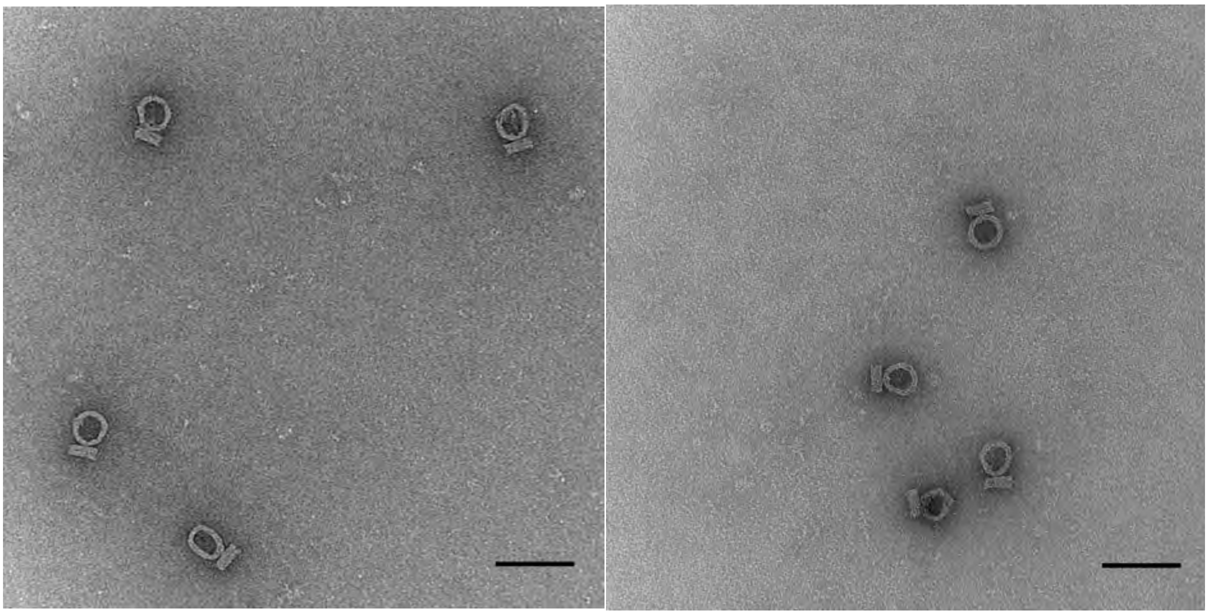

Supplementary Figure 7 l Negative stain TEM images of R2 in the closed state.

Scale bars: 100 nm.

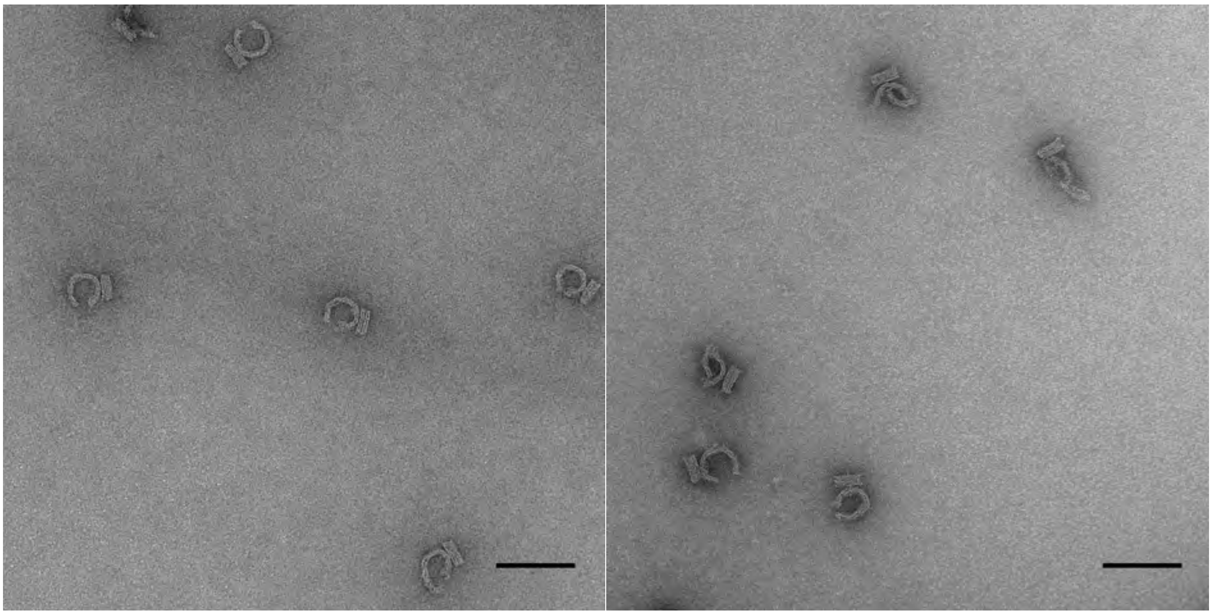

Supplementary Figure 8 l Negative stain TEM images of R2 in the open state.

The left image shows a structure without a flexible hinge. In the right picture, structures with a hinge are shown. Scale bars: 100 nm.

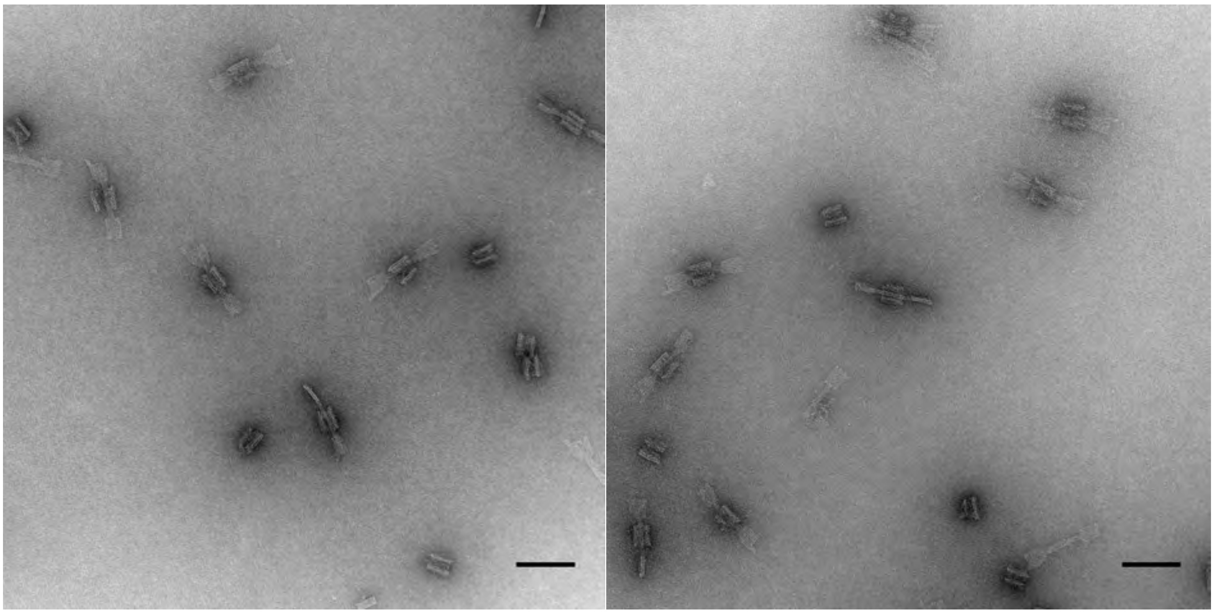

Supplementary Figure 9 | R1D1 rotaxane structures after ring closure. Scale bars: 100 nm.

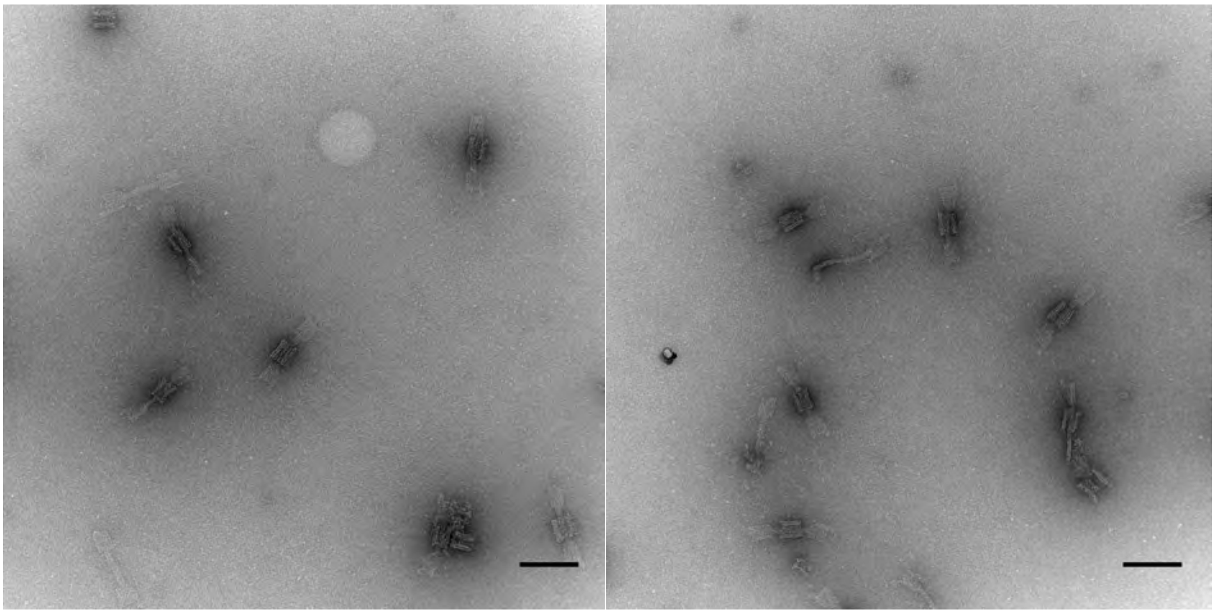

Supplementary Figure 10 | R1D1 rotaxane structures after addition of release strands. Scale bars: 100 nm.

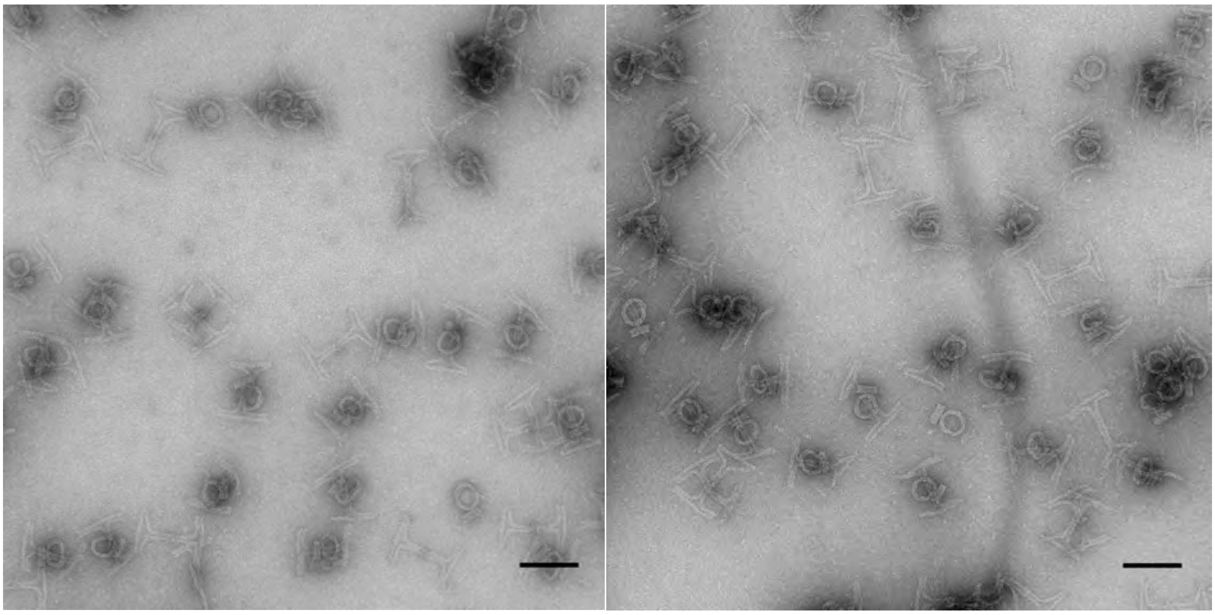

Supplementary Figure 11 I R2D2 rotaxane structures after the ring closure.

Scale bars: 100 nm.

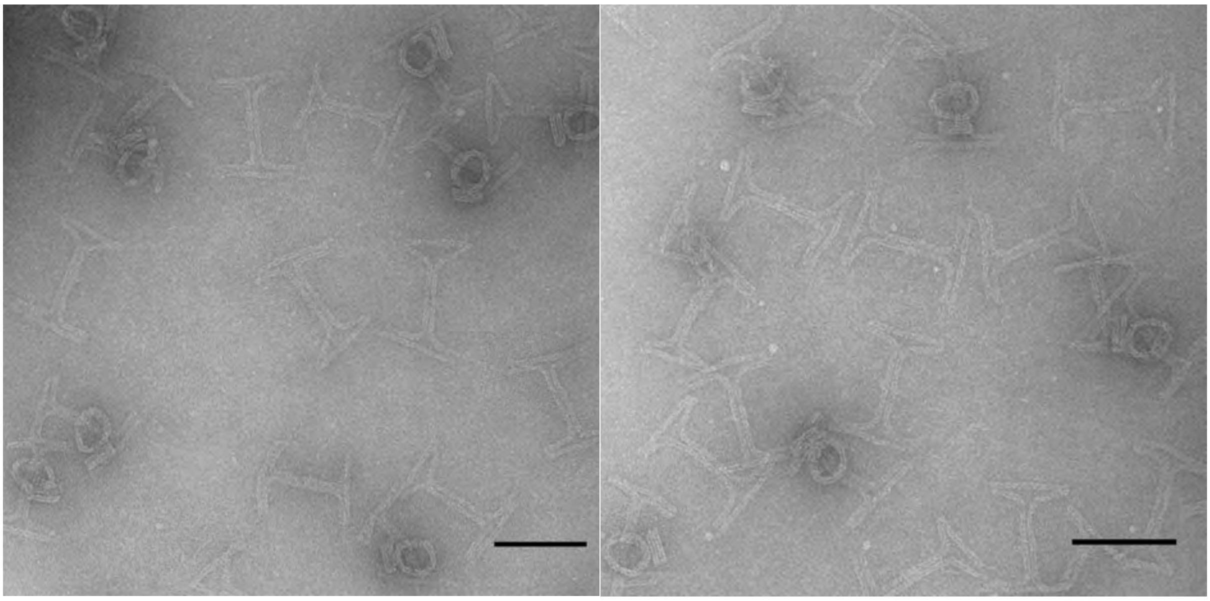

Supplementary Figure 12 I R2D2 rotaxane structures after addition of release strands. Scale bars: 100 nm.

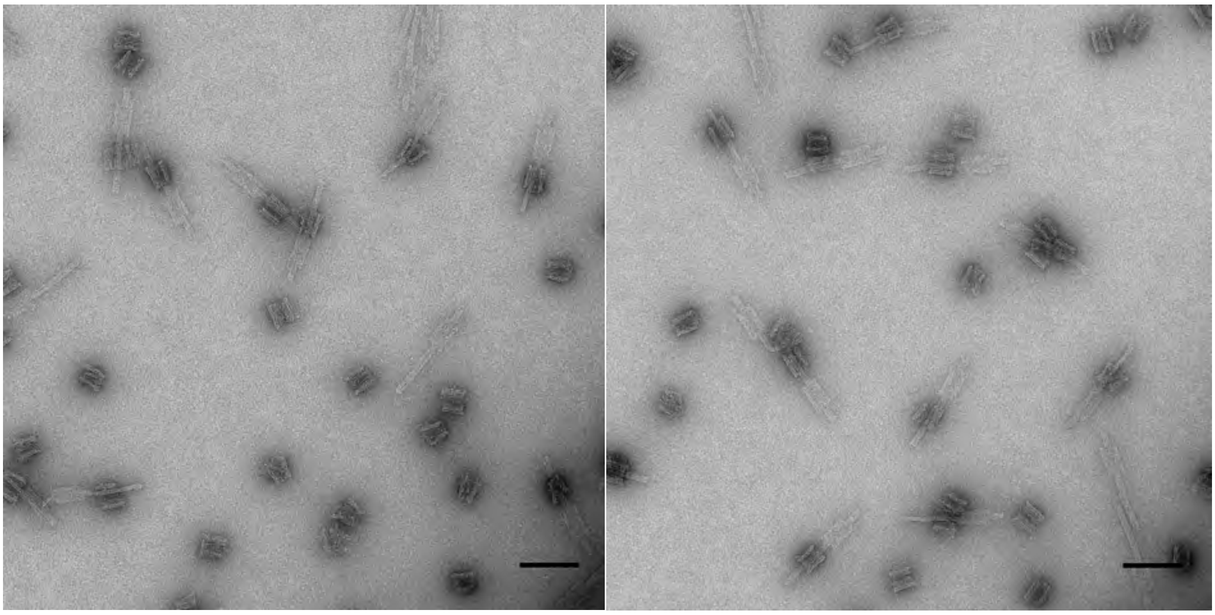

Supplementary Figure 13 I Stopper modules with attached and closed rings.

Scale bars: 100 nm.

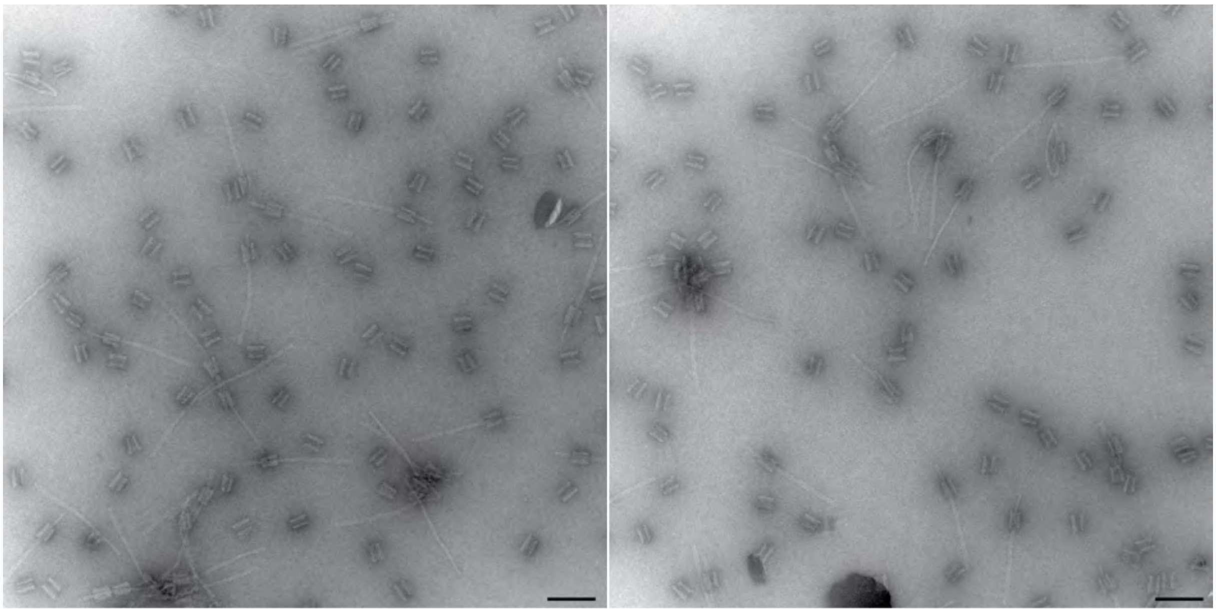

Supplementary Figure 14 I Axle modules with attached and closed rings. Scale bars: 100 nm.

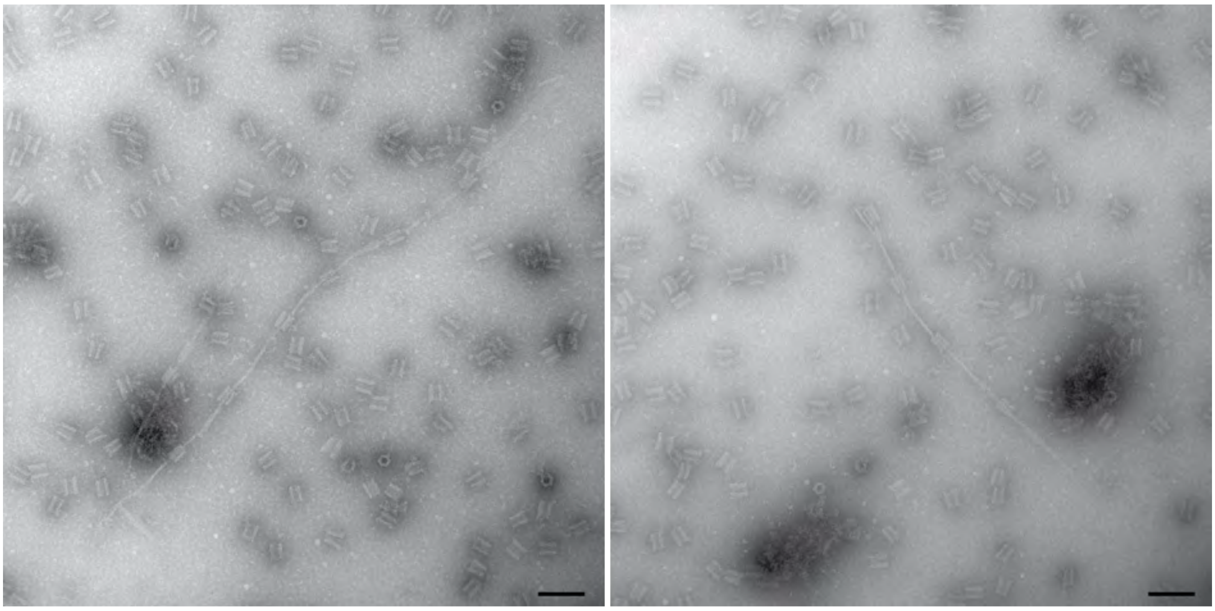

Supplementary Figure 15 I Polymerized stopper module chains with attached and closed rings. Scale bars: 100 nm.

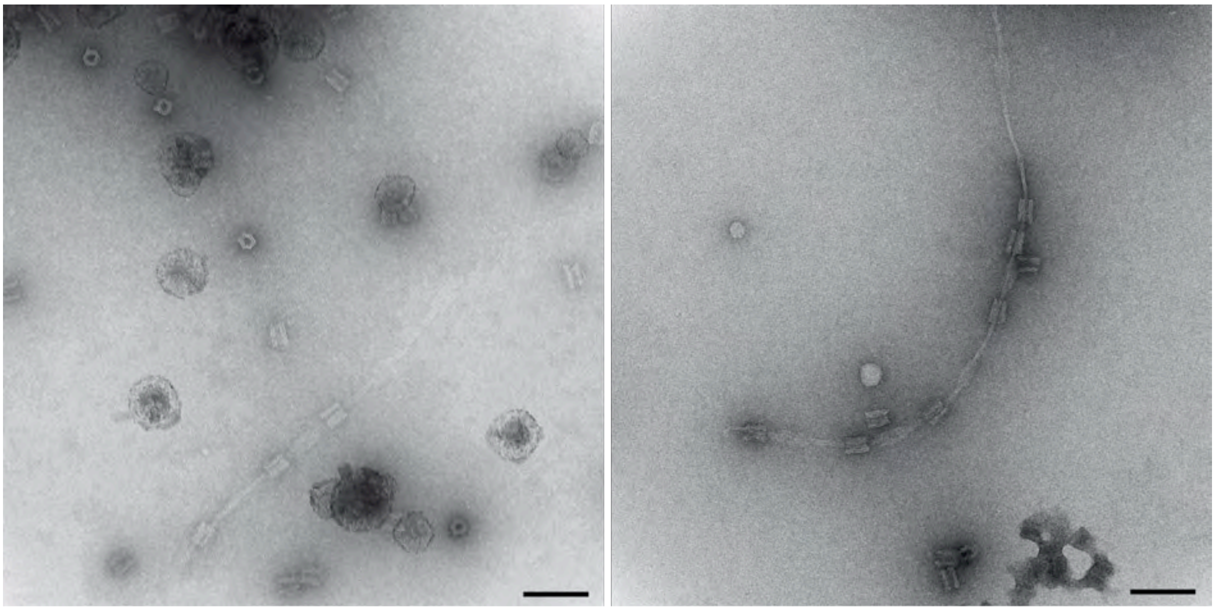

Supplementary Figure 16 I Polymerized stopper module chains with released rings. Scale bars: 100 nm.

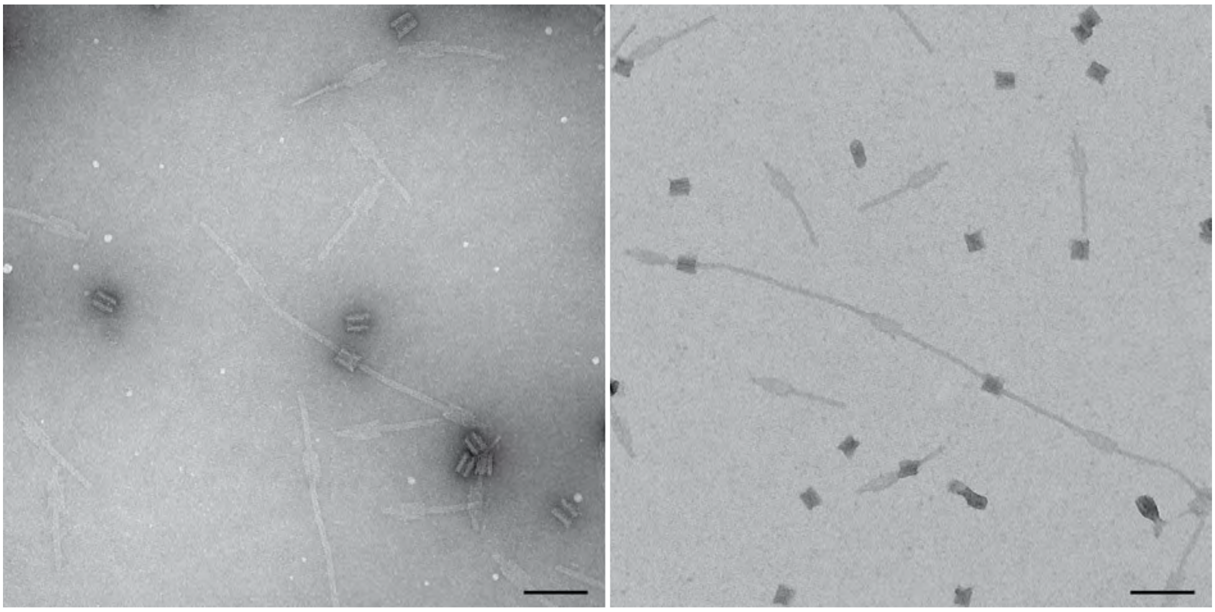

Supplementary Figure 17 l DNA origami rotaxane construct with long axis. Stopper modules with attached rings were connected to both ends of an axle module. A ten-fold excess of stopper modules was added allowing also the formation of rotaxane chains (right). Before the addition of release strands, two rings are attached to one construct, but only one is placed between two stoppers. Scale bars: 100 nm.

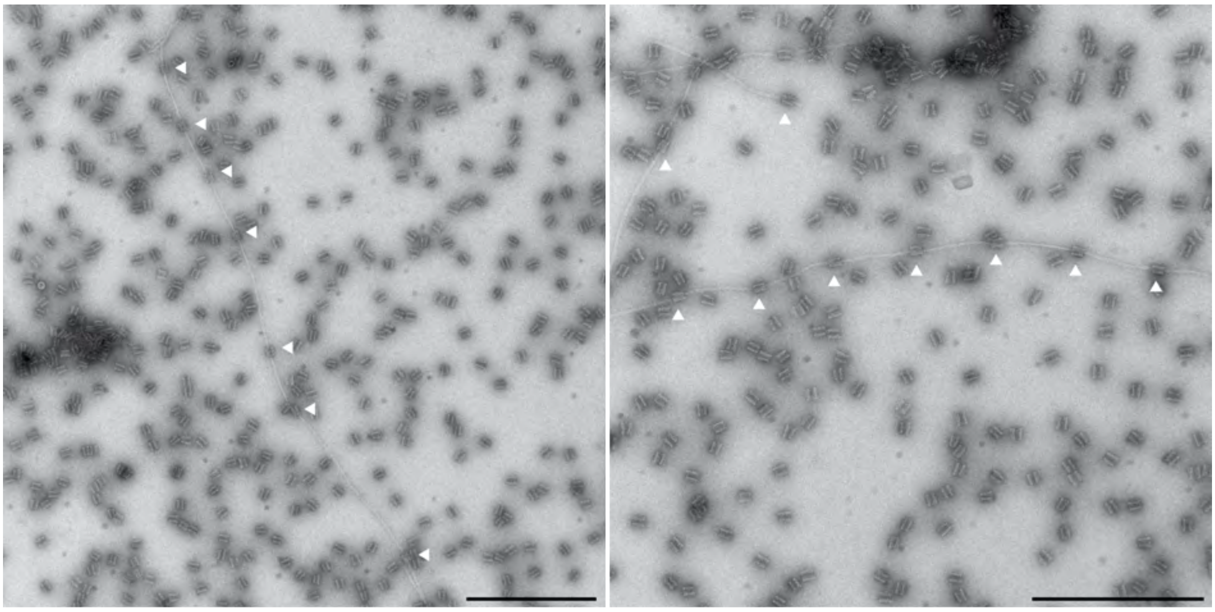

Supplementary Figure 18 l Polymerized axle module chains with attached and closed rings. Scale bars: 500 nm.

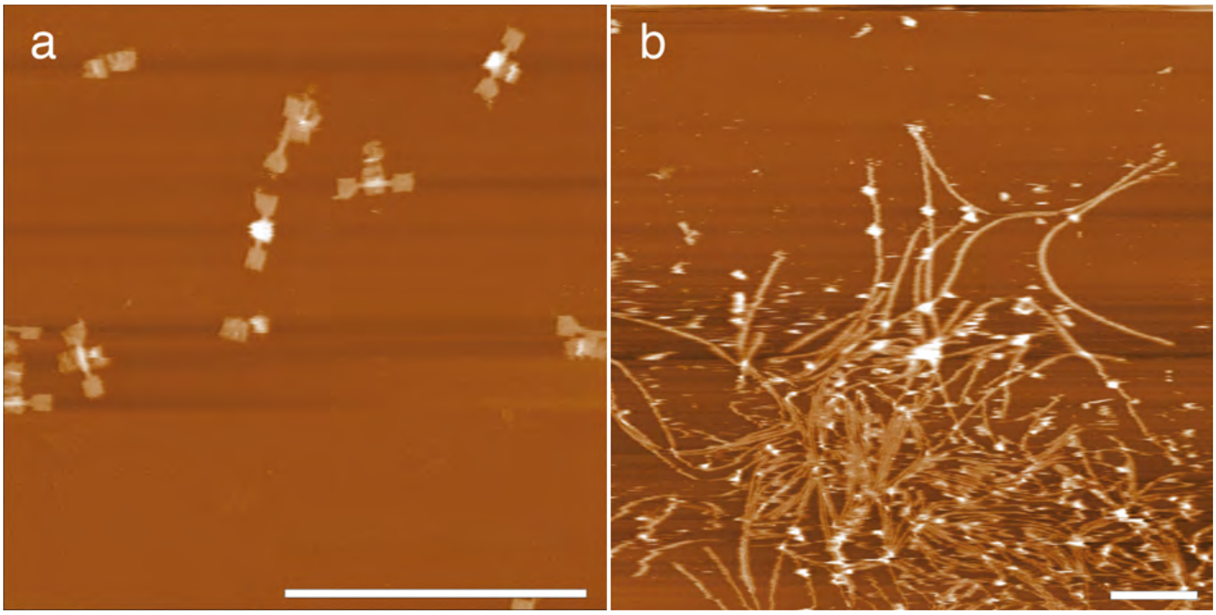

Supplementary Figure 19 l AFM images. a, R1D1 rotaxane. **b,** Polymerized axle modules with attached and released rings. The filaments tend to aggregate on a mica surface when applied in high concentrations. Aggregated pseudorotaxanes are convenient for observation of mobile rings, as the rings cannot escape via the ends and the filaments are sufficiently fixed to the substrate while still maintaining the mobility of the ring. Scale bars: 500 nm.

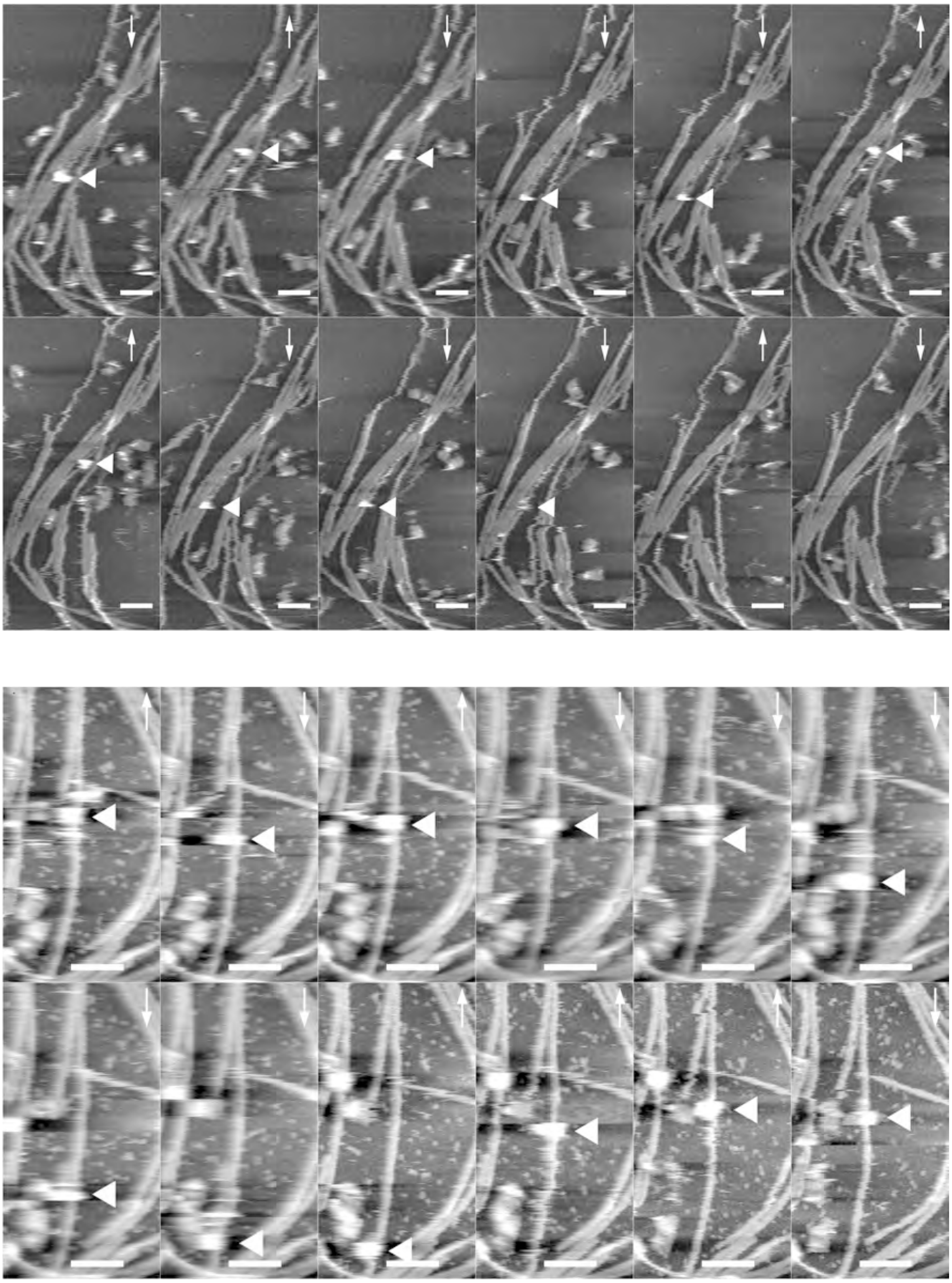

Supplementary Figure 20 l Additional fast scan AFM image sequences. AFM-tip induced motion of R1 along 10 helix bundle filaments. The slow scan direction is indicated by an arrow. Scale bars: 100 nm.

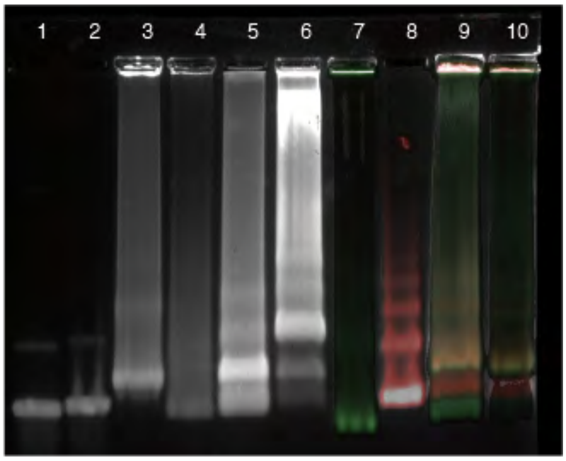

Supplementary Figure 21 I Agarose gel electrophoresis of origami subunits and rotaxanes.

1: 7249 nucleotide scaffold; 2: 7560 nucleotide scaffold; 3: PEG purified D2 subunits; 4: PEG purified R2; 5: R2D2 rotaxane sample after addition of release strands; 6: R2D2 rotaxane sample before addition of release strands; 7: PEG purified D1; 8: PEG purified R1; 9: R1D1 rotaxane sample after addition of release strands; 10: R1D1 rotaxane sample before addition of release strands; DNA was stained using SYBR Gold (white). Cy3 (green) and Cy5 (red) labels were detected using a Typhoon 9500 FLA laser scanner (GE Healthcare). Conditions: 2% Agarose, 100V, 3h, 20 mM $MgCl_2$, 1 x TAE.

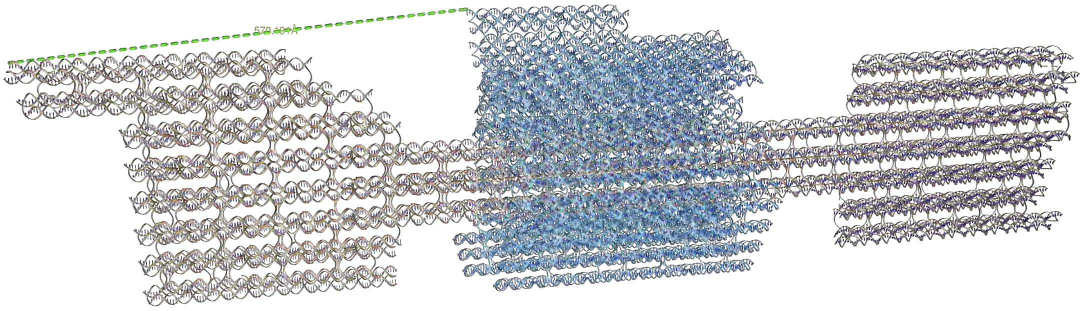

Supplementary Figure 22 I Molecular model of D1 and R1 at the attachment position generated with CanDo and Chimera.

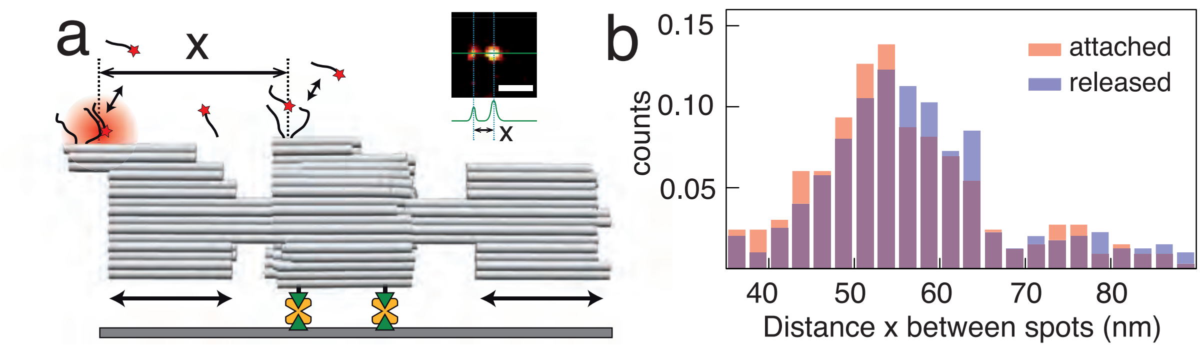

Supplementary Figure 23 I DNA-PAINT experiments. a, Schematic representation of surface immobilization and attachment of PAINT sequences for R1D1. **b,** Histogram of distances x measured in PAINT superresolution images showing the shift of the average ring position for mobile rings. Insert: exemplary point pair of a PAINT image. Scale bar: 100 nm.

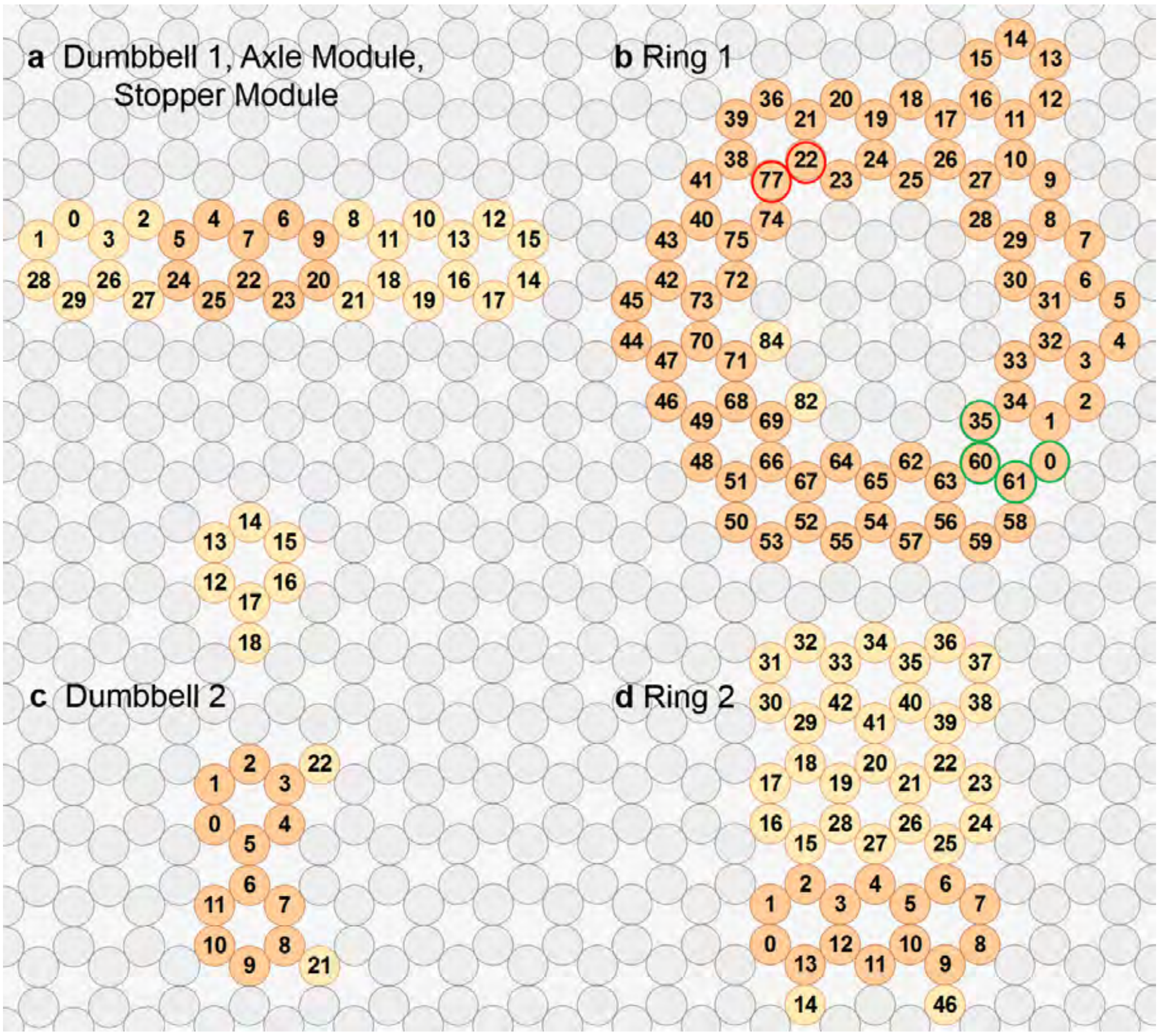

Supplementary Figure 24 I Helix allocation for the structures in caDNAno. a, D1. The axle module and the stopper module have the same helix allocation. Helices 2 and 27 are attachment positions. **b,** R1. Helices without connecting crossovers for the hinge (red) and the opening (green) are marked. Helices 84 and 82 represent the attachment positions. **c,** D2. The dark highlighted helices belong to the axle. At the ends there are no crossovers between helices 5 and 6 and both 6 helix bundles are bent apart in a 90° angle. Helices 21 and 22 are attachment positions. **d,** R2. The dark highlighted helices in ring 2 are bent in a full circle. Helices 14 and 46 are attachment positions.

.

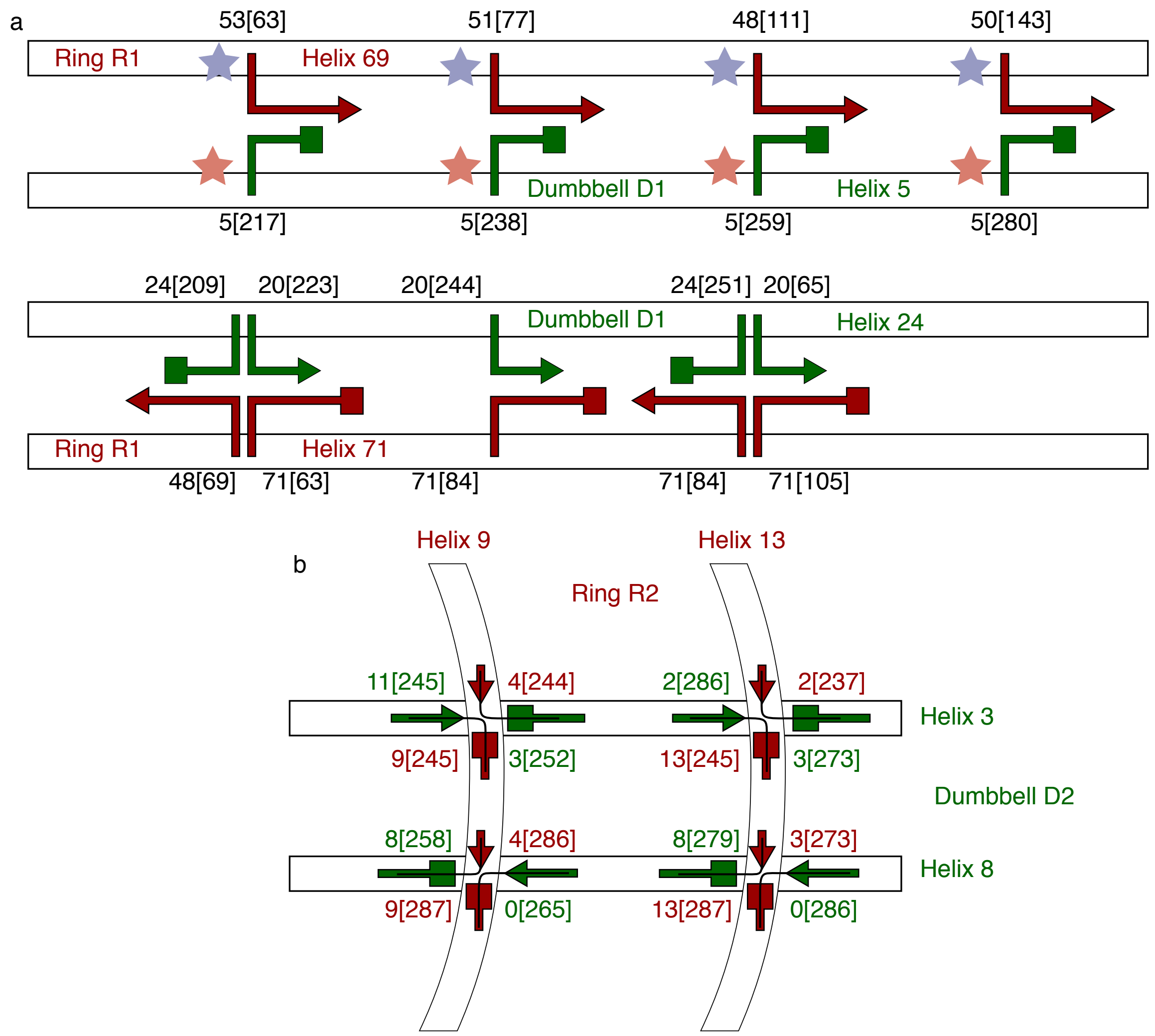

Supplementary Figure 25 I Schematic connections between rotaxane components.

a, R1D1 connections - the five connections to helix 71 of the R1 were used in all experiments, the other four connections were additionally used for AFM, FRET and PAINT experiments. Fluorescent dyes are shown as stars (blue: Cy5, red: Cy 3). **b,** R2D2 connections. The indicated staple ends are extended by complementary sequences connecting corresponding staples of the other subunit (indicated with black lines).

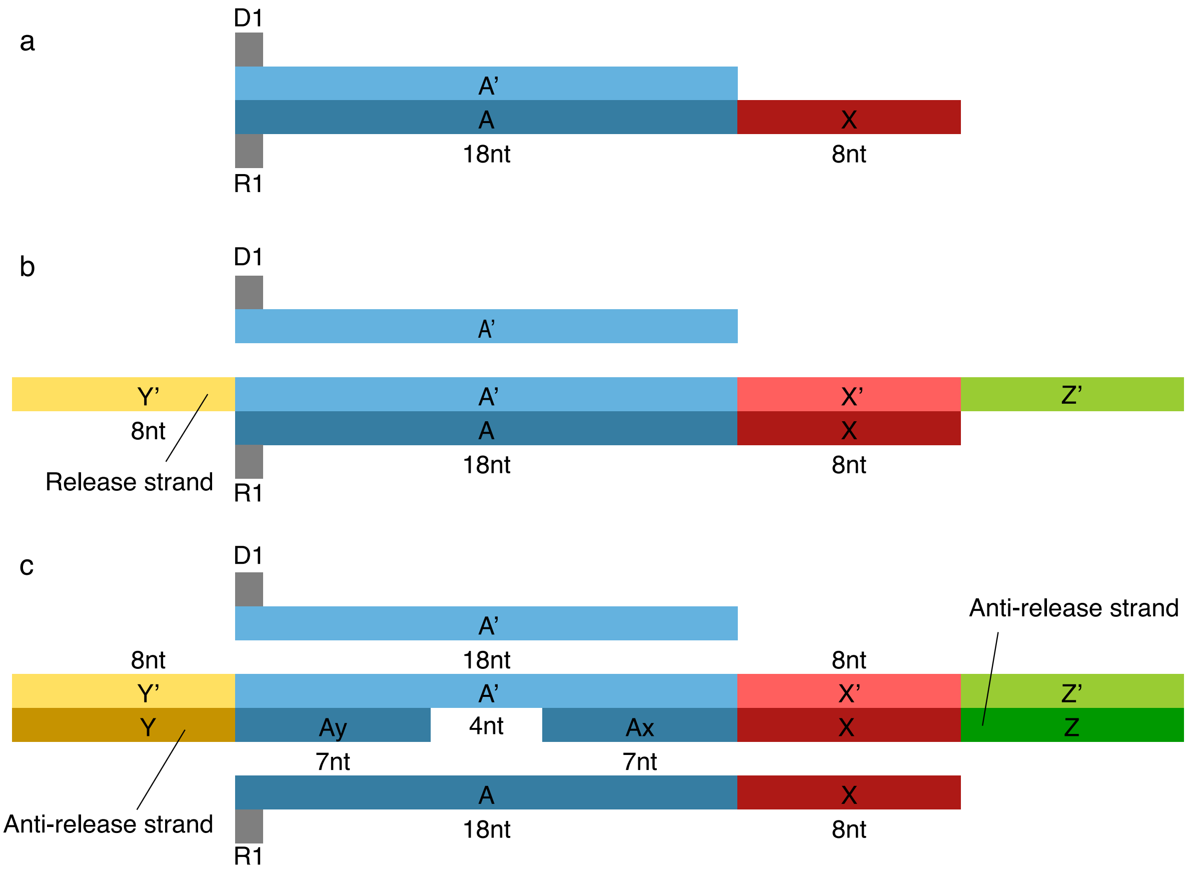

Supplementary Figure 26 | Release strand and anti-release strand design. a, In the attached state, staple extensions are hybridized. One extension carries an 8nt long toehold sequence **b,** A release strand can displace the connection. **c,** A pair of anti-release strands can again displace the release strand, allowing the extensions to hybridize back into their initial state.

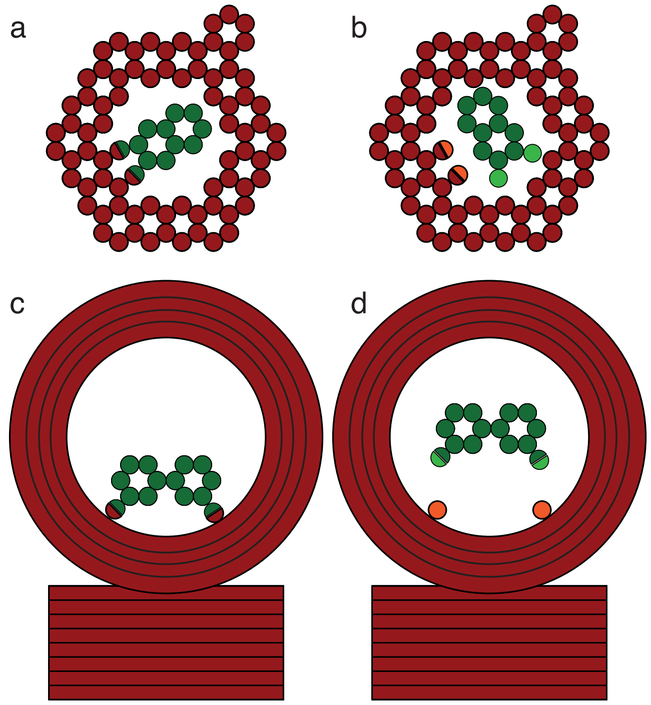

Supplementary Figure 27 | Cross section of the rotaxanes according to design. a, R1D1 before the addition of release strands. **b,** R1D1 after release. **c,** R2D2 before the addition of release strand. **d,** R2D2 after release. Single-stranded DNA is indicated by brighter circles (size according to the radius of gyration for 18nt single stranded DNA; Persistence length 1 nm, contour length 9 nm). Double stranded connections or duplexes of release strand and attachment extension are shown as two connected half circles.

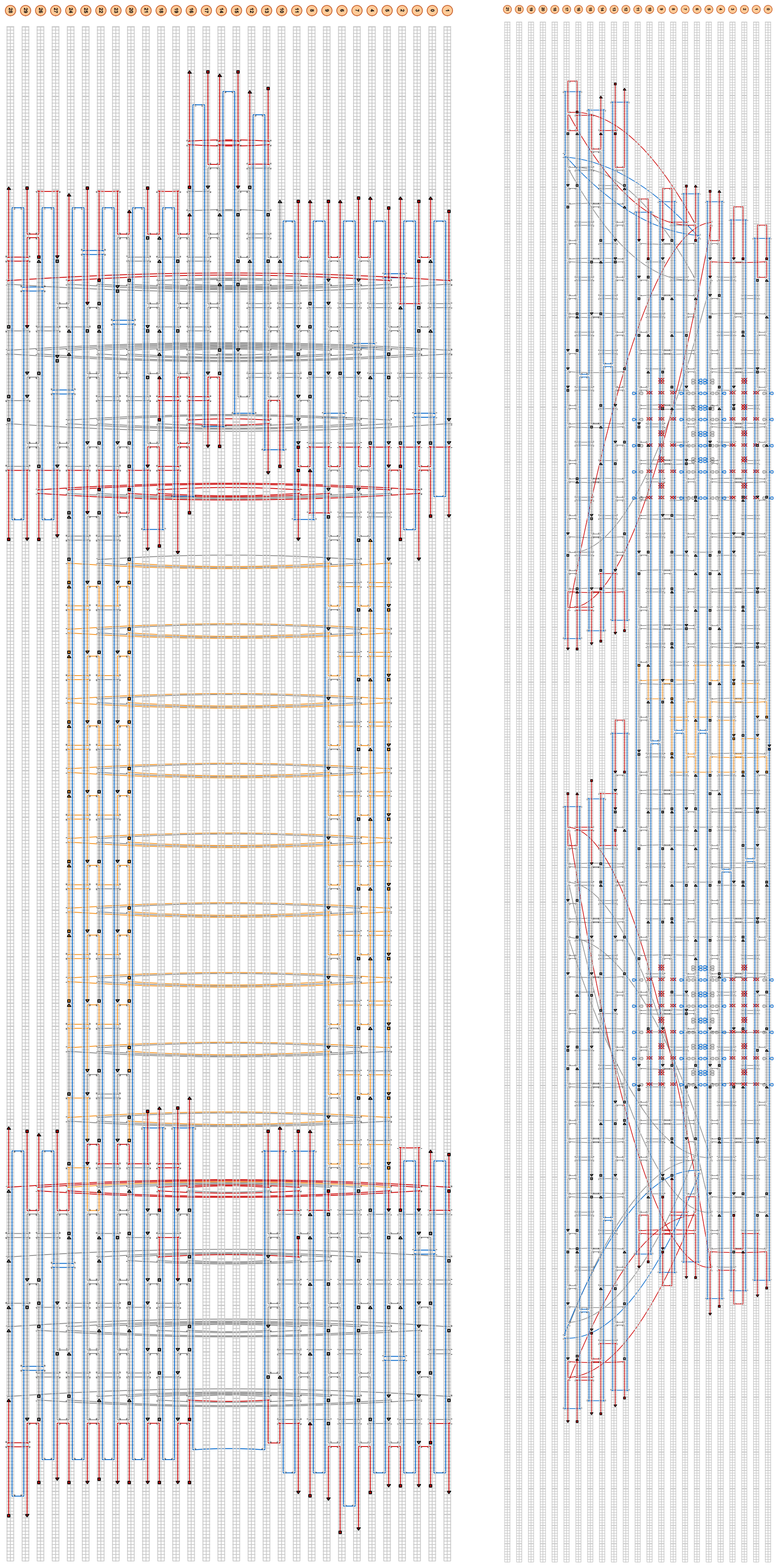

Supplementary Figure 28 lCaDNAno design map for dumbbell 1 (left) and dumbbell 2 (right)

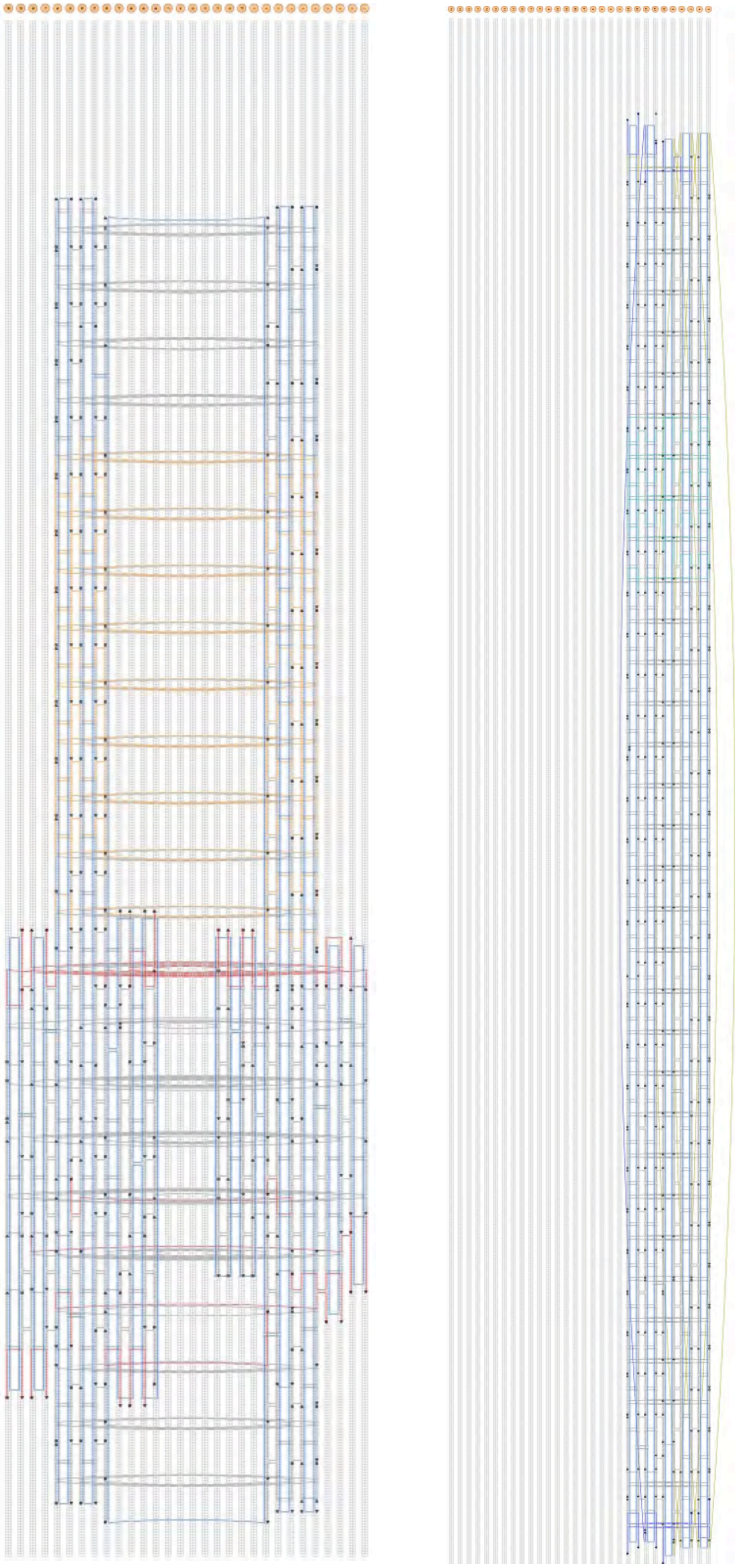

Supplementary Figure 29 lCaDNAno design map for the stopper module (left) and the axle module (right)

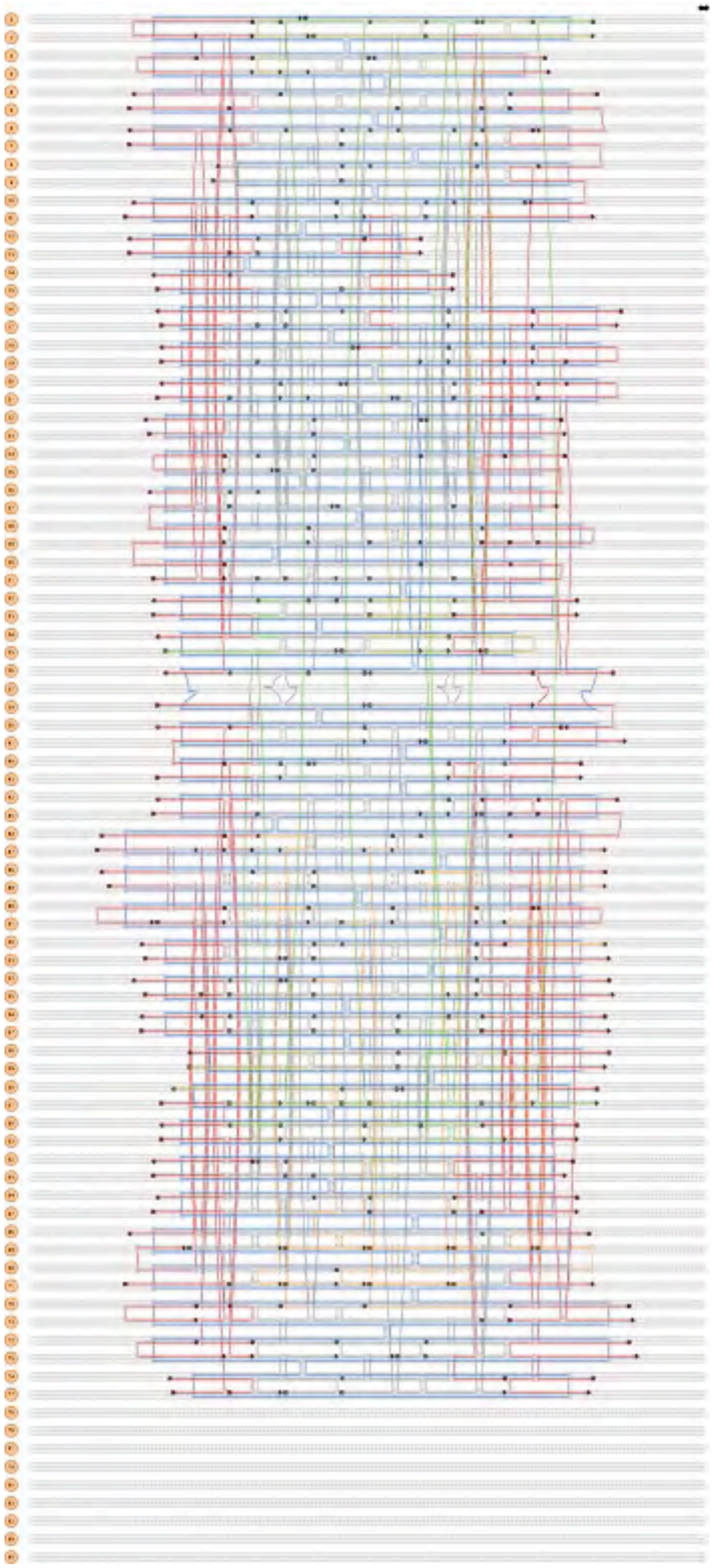

Supplementary Figure 30 lCaDNAno design map for ring 1

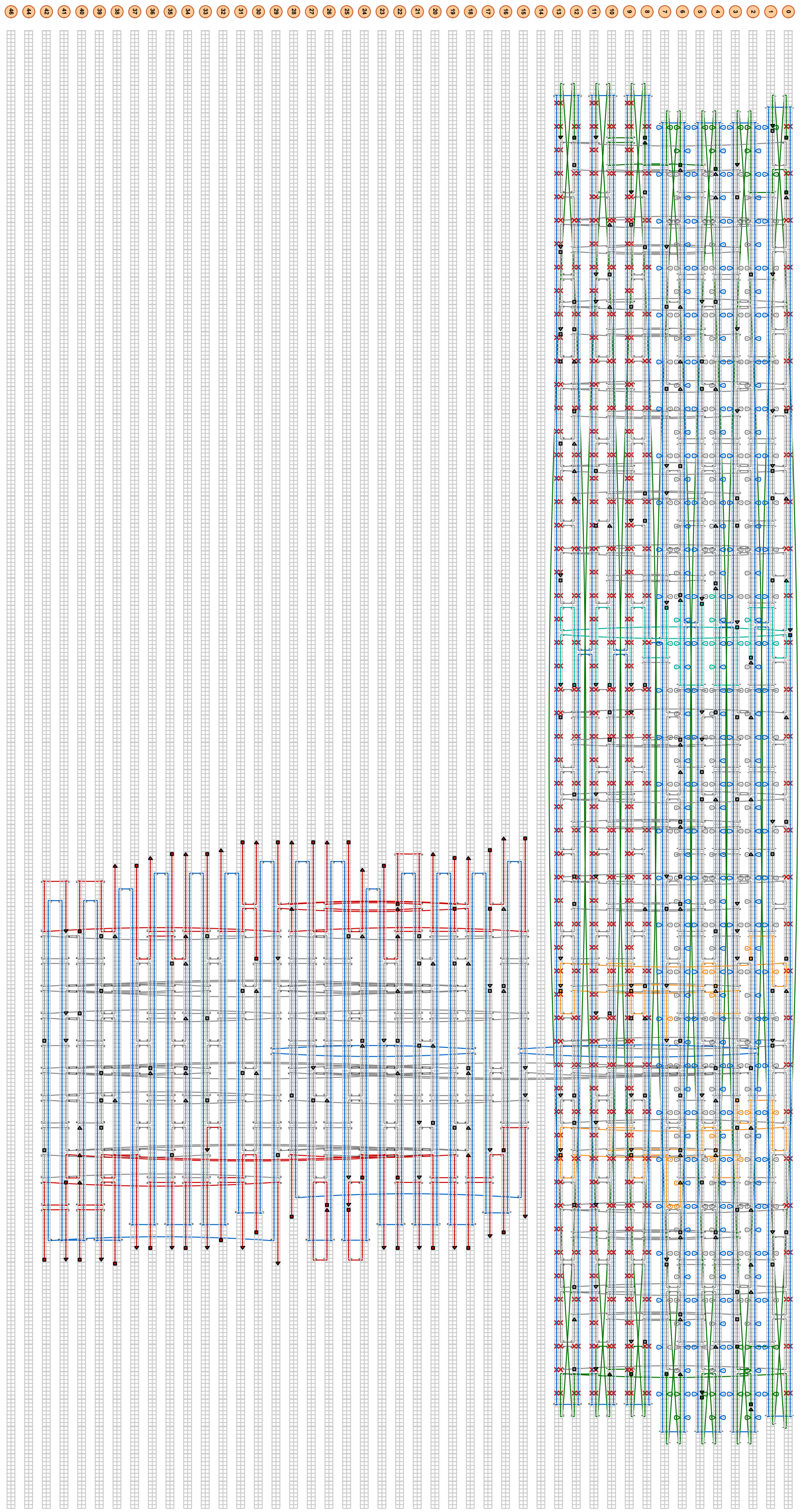

Supplementary Figure 31 lCaDNAno design map for ring 2

Structure	Length	ø Inside	ø Outside	# Helices	Orientation marker
Ring 1 (R1)	35 nm	15 nm	31 nm	76	21 x 5 nm<br>4 helices<br>(not visible)
Ring 2 (R2)	16 nm	28 nm	44 nm	14	33 x 16 x 15 nm<br>28 helices<br>(visible)

Structure	Dimensions	Axle	Stopper elements	Orientation marker
Dumbbell 1 (D1)	143 x 34 x 8 nm	67 x 11 x 8 nm<br>10 helices	26 helices<br>38 x 29 x 8 nm	31 nm x 8 nm<br>6 helices<br>(visible)
Dumbbell 2 (D2)	90 x 88 x 8 nm	60 x 15 x 8 nm<br>12 helices	12 helices<br>71 x 15 x 8 nm	17 nm x 8 nm<br>12 helices<br>(hardly visible)
Stopper Module	169 x 29 x 8 nm	94/15 x 11 x 8 nm<br>10 helices	30 helices<br>60 x 34 x 8 nm	none
Axle Module	246 x 11 x 8 nm	246 x 11 x 8 nm<br>10 helices	none	none

Supplementary Table 1 I Dimensions of the DNA origami subunits according to design.

ramp	Start temperature (°C)	End temperature (°C)
1	64	61
2	62	59
3	60	57
4	58	55
5	56	53
6	54	51
7	52	49
8	50	47

Supplementary Table 2 I Screened folding temperature ramps.

Supplementary Methods

Statistical analysis

Attachment yield. We estimated the yield of correctly assembled structures after rotaxane ring closure from TEM images. To this end the number of unbound structures were counted (D1: 17; R1: 120; D2: 66; R2: 10) as well as the number of fully assembled rotaxanes (R1D1: 96; R2D2: 96). The yield was defined as the ratio of correctly assembled structures and the total number of D1 or R2 particles (the subunit which was not present in excess).

Relative motion. After the addition of a four-fold excess of release strands, samples were incubated overnight at room temperature and stained at the same time as a control sample without release strands. The increase in ring mobility for R1D1 and R2D2 was demonstrated by comparing different states observable in TEM images before and after the addition of release strands and is shown in Fig. 2.

The ring position histogram for the translational motion of R1D1 (Fig. 2 B) shows the difference from the average ring position. For this, the distance between the edge of the stopper at the orientation marker side and the next edge of the ring was measured and the mean value of the bound state was subtracted. 222 particles were measured in the bound state and 197 after the addition of release strands. The rotational state (Fig. 2 E) of R1D1 was determined by counting AuNP functionalized structures (Fig. 2 C) in a clear cis- or trans conformation. There 143 Particles (125 cis, 18 trans) were counted in total for the sample before the addition of release strand and 109 Particles (69 cis, 40 trans) after release.

The longitudinal motion of R2D2 was analysed by counting the number of marker structures which were located at the stoppers or between the stoppers. Before the addition of release strands, only 4 blocks were observed at the stoppers, while a total of 115 blocks were found in the middle of the axle. After release, a larger number of blocks were observed at the stoppers (in total: 25), while 53 blocks were found between the stoppers. Rotational ring movement was characterized by counting the fraction of blocks appearing above or under the axle (before release: 77, after release: 40) or next to it (before release: 42 – after release: 68). As the attachment positions of ring R2 are underneath the block, the markers should appear on the axle for the bound state and more frequently on the side if the ring is released. This analysis can only provide an estimate for the minimal yield, as all of the states should occur for a free macrocycle. The fraction of distinguishable states observed in TEM images may be different from the states occupied in solution due to staining and drying artefacts.

DNA-PAINT measurements

The relative ring position before and after the addition of release strands of R1D1 was also determined using the DNA-PAINT super-resolution microscopy technique, which is particularly well suited for imaging of DNA origami structures. DNA-PAINT is based on the transient binding of short, fluorescently labeled oligonucleotides to complementary strands attached to the structure to be imaged. A DNA-PAINT experiment in total internal reflection fluorescence microscopy (TIRFM) yields a fluorescence video of binding and dissociation events whose intensities can be fitted with Gaussians for localization and super-resolution image reconstruction.

For DNA-PAINT experiments, both components of the R1D1 rotaxane were labeled with extended DNA-PAINT sequences according to the scheme shown in Fig. S23a. The ring was further modified with biotinylated staples. Following previously published protocols, a ≈ 20 µl flow chamber was built from a microscope slide, a glass cover slide (#1.5) and two stripes of double-sided adhesive tape as spacers between the slides. The chamber was coated with BSA-biotin and streptavidin and incubated with rotaxanes (~50 pM) for 5 min in order to allow for the immobilization of the biotinylated origami structures. Eventually the fluorescently labeled oligonucleotides (9 nt length, 20 nM concentration) were flushed in and the chamber was sealed for imaging.

DNA-PAINT imaging of the rotaxane structures was performed on an inverted microscope (Olympus IX71) using objective-type TIRF microscopy with a 100X oil-immersion objective and an Andor Ixon 897 emCCD camera (pixel size: 16 µm). Samples were imaged with a widened excitation laser (Toptica iBeam smart, λ=642 nm, typical laser power: 50 mW) and the camera set to 128 x 128 px^2 (4x binning), 5000 frames, 50 ms integration time, 250 EM gain. Super-resolution images were reconstructed and analysed using a custom MATLAB® program.

We used a molecular model of R1D1 (Figure S22) generated from CanDo simulations of both components to estimate the distance between the PAINT positions. For the attached state a distance of about 57 nm is found, while the minimum and maximum distances accessible in the released state are 47 nm and 79 nm, respectively (including both translation and rotation of the ring).

Point pairs of the super-resolved images representing ring and dumbbell were aligned, fitted by two Gaussians and the distance between the centres was measured. A histogram of the distances x before and after the release is displayed in

Fig. S23b. Only point pairs between 35 and 90 nm were analyzed as they were considered to represent the accessible range with an additional localization accuracy margin of 10 nm. The histograms were generated from 332 (bound) and 399 (released) particles. After the addition of the release strands the mean distance between point pairs (bound: 55.68 ± 0.58 nm; released: 57.33 ± 0.55 nm;) increased by 1.65 nm. In DNA-PAINT measurements, only a single state was observed, and not a two-state distribution as in TEM analysis. This may be explained by time-averaging between both states, as the motion of the rings presumably is faster than the acquisition time for a super-resolved image. Indeed, the average ring positions determined from averaging over the full histograms shown in Figure 2 of the main text shift by 1.7 nm upon release, which is comparable to the value found here.

Origami design

Avoiding base stacking interaction Passivating thymines (3-8 nt) were added to staples at the ends of helices in order to avoid unspecific base stacking interaction and aggregation. We omitted these poly-T sequences for helix ends which were later connected to other helix ends (polymerization and closing of R2). In these cases, single-stranded scaffold loops required for the addition of further staples acted as passivation.

Hinge. A flexible hinge was introduced into R1. Crossovers between helices 22 and 77 were therefore omitted (Fig. S23b). 3 thymines were inserted into crossovers between helices 39 and 36 for a higher flexibility. A similar hinge was designed for R2. By leaving out a set of staples in a ring segment, the ring could be separated into two loosely connected half circles. This version was not used in further experiments as the large single stranded regions interfered with the purification method. Even without

this hinge, the ring was flexible enough to provide an opening for the axle to enter (Fig. S10).

Closing staples. In the open state, staples of R1 connecting helices 35 with 60 and 0 with 61 were not added. These 7 staples were later used as closing staples. The closure of the ring was established via crossovers between parallel helices. In the case of the open ring R2 a connection between helix ends had to be established. Therefore a set of 14 closing strands was designed to bridge this gap.

Temporary connection. During the rotaxane assembly, a connection between the subunits had to be established. For both subunits, selected staples were extended with complementary 18 nucleotide long sequences. A toehold of 8 bases was added to one of the two sequences. In the case of R1D1 four connections were omitted and their positions were used for dye modifications. TEM images were recorded for samples with 5 connections otherwise 9 connections were used. For R2D2 8 connections arranged on the corners of a rectangle were used (Fig. S24).

In the course of the FRET spectroscopy experiments, anti-fuel strands were used to localize the ring again at its initial attachment position (Fig. S25). Two anti-fuel strands were necessary to displace one releasing fuel strand. A single long single anti-fuel strand would be complementary to the other connection staple and would therefore also act as a fuel strand. Fig. S26 shows a cross section of the rotaxane, displaying the crowded situation inside the macrocycle especially for R1D1.

Folding Temperature We screened the folding temperature for all structures. First, the mixture was heated for 15 minutes at 65 °C followed by a slow annealing ramp. For all structures we tested eight different 4°C ramps over 4 hours (temperatures in

supplementary table 2) and identified the best folding result by agarose gel electrophoreses and TEM imaging.

PEG purification. In order to provide a faster attachment of the two rotaxane subunits with high yields, high concentrations of the subunits without remaining excess staples was necessary. Therefore we used the PEG purification method where it was possible to increase the final concentration from 50 nM of structures to 200 nM. We also performed a buffer exchange to a sodium rich buffer to avoid aggregation at high concentrations of structures. This method resulted in a yield of nearly 100 % for R1, D1, D2 and the stopper module. The purification was less reliable for the axle module and almost unprofitable for R2 with hinge. Without the hinge the yield could be drastically increased for R2. The pellet of axis modules and R2 produced by centrifugation only dissolved after shaking at 37 °C. These hard to purify structures all contained extended regions of single stranded DNA presumably causing low purification yields. Filter purification of these structures resulted in even lower yields.

Gold nanoparticle (AuNP) functionalisation For attaching nanoparticles to origami structures, we coated the AuNPs with DNA and enable it to specifically bind to their attachment site. The DNA coating also prevents aggregation of the particles in the presence of divalent ions. In contrast to other studies, we did not use a poly T sequence, as this sequence was already used for passivation, but a periodic TC sequence. The sequence used for attachment was chosen orthogonal to all previously used sequences and without intramolecular interaction to avoid unspecific binding and aggregation. A periodic sequence was used as it enables a sliding

mechanism for the binding process. We chose three staple extensions per binding site for one nanoparticle, where each of the three connections was individually thermodynamically stable. The redundant binding sites limit the passivating effect of possible traces of unbound thiol strands left in the purified AuNP solution.
