# Supplementary material for: Long-range movement of large mechanically interlocked DNA nanostructures
Source: Nat Commun. 2016 Aug 5;7:12414. doi: 10.1038/ncomms12414 (PMC4980458; doi:10.1038/ncomms12414)
Supplement: Supplementary Data 1 — Contains a list of the DNA sequences used for the construction of the DNA origami objects [file ncomms12414-s2.docx]

# DNA sequences

Staple strand sequences for dumbbell 1, dumbbell 2, the stopper module and the axle module. Core staples are indicated by gray bars, passivation staples by red bars, connection staples by yellow bars, adaptors to fluorophores by green bars, adaptors to biotin by blue bars and adaptors for the PAINT sequence by purple bars. Release strands and anti-release strands are not highlighted with color. The yellow marked connection staples and ref_verb staples are used in TEM experiments. For all other experiments all ref_verb staples were replaced by connection staples.

Dumbbell 1

| Start | End | Oligoname | Sequence | Length |
| --- | --- | --- | --- | --- |
| 0[363] | 29[377] | 0[363] | ATTCACCGGCCAACAGAGATACTGGCTGACGTAACAAAGCTG | 42 |
| 22[146] | 9[139] | 22[146] | TTGTCGTTTCAGGTGTATCATATGCGTTCAACAGT | 35 |
| 9[378] | 3[384] | 9[378] | TCAATAAATAGGTCATATGTGAGGAGCACTTGCTGTGCAACAGAGGTGA | 49 |
| 1[392] | 3[391] | 1[392] | GTAAGAATTAAAAATACCGAAGGCGGTC | 28 |
| 5[98] | 2[84] | 5[98] | CGCCGCGACCATATCAGCCCTTCACCGCGTAACCAGGCAAGT | 42 |
| 22[230] | 9[223] | 22[230] | CACTGAGATACCAAGGTTTGAAATACCGGAGAATA | 35 |
| 9[413] | 19[419] | 9[413] | ACGGGTACAAGCCGTTTTTATAGTTGCTGGCATGAAGGAAACCGAGGAA | 49 |
| 25[91] | 7[104] | 25[91] | GGAATTGCGAATAAGATTTTGATTATTT | 28 |
| 9[420] | 3[426] | 9[420] | TTAAACCATTAAGAAATTAATAACAGTTTCAATATAATCTAACGCTGAG | 49 |
| 24[363] | 28[350] | 24[363] | CAGGACGTTAAGAATGGTTTACAAATCAACCTTCATCAAGAG | 42 |
| 22[335] | 9[328] | 22[335] | TTAGTACCAATTTCTAACTATATGTAAAAGTCCTG | 35 |
| 4[356] | 20[350] | 4[356] | CCGTCAATAGATAATTACCTTCGTACTCCAGTTAATGCCCCCAATTAGA | 49 |
| 15[70] | 18[63] | 15[70] | GATTGACGGAACGCCATCAAAAAACAGGGGAGCAATAGGTAATTAAATG | 49 |
| 11[98] | 17[104] | 11[98] | TCCCCGGTGGGTAACGCCAGGCGGGCCTAGGCTATTAAATTGTAAACGT | 49 |
| 15[77] | 11[90] | 15[77] | CGTAATGGGATAGGGACGACGATCGGTGGTTTTCCTCGACTC | 42 |
| 9[357] | 3[363] | 9[357] | TTACGAGTTTAACCGGAAACAATTAGAGAGAAGAAGGAAATAATTATTT | 49 |
| 16[377] | 8[378] | 16[377] | GATAACCGCAGCCTTTACAGAAGTTACAGGAGGTTCGCGCCC | 42 |
| 7[259] | 23[272] | 7[259] | TCATTTCGGATAGCCATACATGGCTTTT | 28 |
| 27[100] | 5[125] | 27[100] | GGTGTCTGGATTAGACTGGATAGCGTGGTAATAACAGGGC | 40 |
| 8[111] | 11[118] | 8[111] | CGAGCCGGTCGTGCCAGCTGTATAAAGCTTCCACACAACATAGCTCGAA | 49 |
| 19[70] | 12[70] | 19[70] | AGATTCAAAAGGGTTTGCCTGAAGGGCGACAGTATCGGCCTC | 42 |
| 7[322] | 23[335] | 7[322] | CATTTAACGCCACCGAGTAACAGTGCCC | 28 |
| 26[412] | 5[419] | 26[412] | ATCCGCGAAGTACAACGGAGACCAACCTCCCTCAA | 35 |
| 21[63] | 23[78] | 21[63] | ACATCCAAATTCTATTTAGCTATATTTTCA | 30 |
| 20[363] | 22[371] | 20[363] | ATTTGGGTGCCTATAATAGGTCGGCTACAGAGGCTTTGAGGATAAGTAT | 49 |
| 15[56] | 10[52] | 15[56] | GGGAACAAACGGCGAGGAAGAGCGCAACTAAAACGACGGCTTTTTT | 46 |
| 13[56] | 17[48] | 13[56] | TCAGGCTTCGCACTCCAGCCATCTCCGTCGCGTCTGGCCTTCTGATAAT | 49 |
| 27[399] | 3[405] | 27[399] | CGAAACAACGACGAGAAACACAGTATTAACACCGC | 35 |
| 8[90] | 22[91] | 8[90] | AAAGCCTCGCTTTCGGAGAGGCAACAGCTGATTAACTAAACA | 42 |
| 7[392] | 21[398] | 7[392] | AACCTTGAATTTATCAAAATCTCGGCTGTGAATTATGGTTTACCAGCGC | 49 |
| 22[314] | 9[307] | 22[314] | CCGCCACAACAAAAGCAAATCCAATCGCCCTGTTT | 35 |
| 13[406] | 19[405] | 13[406] | AAGCGCAAAAGTCAGAGGGTATACCAGA | 28 |
| 7[413] | 21[419] | 7[413] | CGCTATTCGCTGAGAAGAGTCTCCAAGAACGGAAAAAAAGGGCGACATT | 49 |
| 22[188] | 9[181] | 22[188] | TTCCACATACATCGGAATAAACACCGGACAACATG | 35 |
| 3[84] | 29[104] | 3[84] | CAAAATCGGAACAATATCACGATGGCCCAGCAAACGACTATTATAGTCA | 49 |
| 9[392] | 19[398] | 9[392] | TCTTTCCGTAGGAATCATTACTTGAAGCTACGCAGTAGCCGAACAAAGT | 49 |
| 19[91] | 15[97] | 19[91] | GCCGGAGACAGTCATCTACAACTTCGCTTGCCAGTTTGAGGGTCACGTT | 49 |
| 7[371] | 21[377] | 7[371] | AATCAATTGAGAGACTACCTTCATGTAGTTGAGCCACAATCAATAGAAA | 49 |
| 22[293] | 9[286] | 22[293] | CCCTCAGTGATGAAAGAACGCGAGAAAAGTTCAGC | 35 |
| 18[377] | 13[370] | 18[377] | GCAAACGACTTGCGAAATAAACAGCCATGAAAATA | 35 |
| 22[167] | 9[160] | 22[167] | TTAGCGTATATACATTACTAGAAAAAGCAATCGCC | 35 |
| 26[104] | 24[91] | 26[104] | CGAGAATAATCAAAGGCGCTAGGGCGCTCCACACCTATAACA | 42 |
| 7[217] | 23[230] | 7[217] | GCTTTGATTTCGTCAATGGAAAGCGCAG | 28 |
| 7[301] | 23[314] | 7[301] | TCAAGAACCTCAGATTTTAACGGGGTCA | 28 |
| 5[105] | 3[125] | 5[105] | CTTAATGAGGAGCGAGAATAGCCCGATAAAGGGAG | 35 |
| 28[118] | 29[111] | 28[118] | TTCAAAGAGCGGATTGCATCATGCTTTAAACAGTTCAGAAAAGAAGCAA | 49 |
| 21[91] | 18[98] | 21[91] | AAGAATTAGCAAAACGCAAGG | 21 |
| 8[83] | 18[70] | 8[83] | GGGGTGCCTAATGATGCATGCATATATT | 28 |
| 22[251] | 9[244] | 22[251] | ATAGGAACAGAGGCATCTTCTGACCTAAAAGGTAA | 35 |
| 20[328] | 25[321] | 20[328] | ACCATTACCATTAGGTGCCTTCTCAGAACACCCTC | 35 |
| 27[70] | 7[76] | 27[70] | CAACATGTTTTAAACCCAATTGCTGCGCCTGGCCCTGAGAGAACCAGTG | 49 |
| 26[377] | 27[384] | 26[377] | AATTGGGCTTGAGACTGGCTCATTTGACCCCCAGC | 35 |
| 20[76] | 10[70] | 20[76] | TGGCATCATAAATCATACAGGAACCCTCCTGCAGGCAGTCACGACGTTG | 49 |
| 16[356] | 8[357] | 16[356] | TAAGCCCTTAACGTCAAAAATATTATTTTAGCGAACAGATAT | 42 |
| 20[160] | 24[147] | 20[160] | AAAATCACCGGAACACAGGAGTAAAGTTAGGAGCCCTTTTGC | 42 |
| 7[196] | 23[209] | 7[196] | CAATAACCAACGCCATAAATCCTCATTA | 28 |
| 7[280] | 23[293] | 7[280] | AAGAAGAAGCCACCCAGGAGTGTACTGG | 28 |
| 29[385] | 29[419] | 29[385] | AGTGAATAAGGCTTGCCCTCTGCTCCATGTTACTT | 35 |
| 7[350] | 21[356] | 7[350] | TTTTAATTCCGGCTTAGGTTGTATCCCAGCCAGCAACGGAATAAGTTTA | 49 |
| 16[370] | 21[363] | 16[370] | CACAAGAATTGAGTGAAGCCCTACATACTTTTGTC | 35 |
| 23[79] | 9[104] | 23[79] | TTTGGGGCGCAGAGCCACCACCCTCACAGAACCGAAACCT | 40 |
| 22[125] | 4[105] | 22[125] | GTAAATGAATAAAGTTCTTACCAGCATTAATGAATGCACGTAAGAACCT | 49 |
| 28[111] | 0[91] | 28[111] | CGAACCAGACCGGAACTACGTTGTTGTTCCAGTTT | 35 |
| 14[104] | 11[111] | 14[104] | GTTAAATGGTGTAGATGGGCGGTGCATCATTACGCATTAAGTGTACCGA | 49 |
| 24[160] | 25[167] | 24[160] | CGAGAGGTTTAATTGTATCGG | 21 |
| 26[391] | 5[412] | 26[391] | CAGAACGGATTATACCAAGCGAAAAGAACCAGAACAATCAAATATCAAA | 49 |
| 12[48] | 14[56] | 12[48] | GCTTTCCGCGCCATTCGCCATAATCGATCAGAAAAGCCCCAAAATAATT | 49 |
| 23[364] | 8[364] | 23[364] | TTCGGAACCTATTACACCGACAAACCAAAATAGCAAGCAAAT | 42 |
| 18[398] | 13[391] | 18[398] | TCCTTATCTTAAATGAGCCTAATTTGCCGAGAATA | 35 |
| 24[321] | 4[301] | 24[321] | ACGGAACAACATTACCGTTGTTTTACAAACAATTC | 35 |
| 6[104] | 9[83] | 6[104] | CGGCCAACGCGCGGCAGTCGGGCCACCCTCGAGCTGAAAAGGCACTGCC | 49 |
| 5[364] | 22[364] | 5[364] | CTCAAACTATCGGCCTAACAACTAATAGGTACATAAGCCCGG | 42 |
| 0[384] | 28[385] | 0[384] | CGAACCATCTGACCTGAAAGCTGTACAG | 28 |
| 20[384] | 24[364] | 20[384] | TCACCGTTTCTGAAGTTGATACTAAAGAAAACACTCATCTTATACCAGT | 49 |
| 9[147] | 5[160] | 9[147] | AATTGAGCTGTTTATTAACGTATCCTGAGAGCACGTATAACG | 42 |
| 16[391] | 21[384] | 16[391] | GCTAATATCAGAGAAAGCAGATATGTTAATTCATA | 35 |
| 22[90] | 26[70] | 22[90] | ACTTTCAAACTAAAGTTGATTTATGCAAAATCAAAAATCAGGATGGCTT | 49 |
| 1[371] | 2[364] | 1[371] | GAACCCTCCAGCAGAAGGCAGACATTGATAAAACAGGAAAAACGCTCAT | 49 |
| 26[384] | 28[371] | 26[384] | AGTAGTACTCATTCACCAGGCGCATAGG | 28 |
| 24[412] | 22[413] | 24[412] | AAAACGAATTAAACGGGTAAATGCTCAG | 28 |
| 1[413] | 3[412] | 1[413] | AATATTTGATAGCCCTAAAACCTGCAAC | 28 |
| 4[125] | 23[125] | 4[125] | ATAATGGAAGGGTTAAACAGAAATTTTCCACCAGAACCACCA | 42 |
| 2[125] | 24[98] | 2[125] | GGGAAGAAAGCGAACGCCGCTGTAAAATGTAGTTTCATTCCA | 42 |
| 18[419] | 10[406] | 18[419] | AAGAACTATTTTGCACGCTAACGAGCGT | 28 |
| 22[83] | 9[76] | 22[83] | ACAGTTTAGACGGGCGGTTTGCGTATTGTTGCGCT | 35 |
| 5[385] | 22[392] | 5[385] | GTAATATTACACTACTTTTTCATGAGGAGTGCCGT | 35 |
| 16[398] | 8[399] | 16[398] | ATTGAGCACATAAAAACAGGGCTTTCCACAAGATTTTTCATC | 42 |
| 7[154] | 23[167] | 7[154] | CAGATGAAACGATCGTTGAGGCAGGTCA | 28 |
| 26[132] | 1[125] | 26[132] | CCTCAAAAAAAGATGTTTTAATTCGAGCATCAAGT | 35 |
| 20[125] | 11[125] | 20[125] | CTCCCTCAGAGCCGATAAAGCCTCAGAGCGGGAGATTCGTAA | 42 |
| 21[105] | 25[125] | 21[105] | TTAAGCACCACCCTGAGCCGCTGTATGGTAATTTTTTCACGTTGAAAAT | 49 |
| 0[405] | 28[392] | 0[405] | ATCGCCATACGTGGCACAGACTGAAAGAGGACAGATGAACGG | 42 |
| 13[70] | 14[77] | 13[70] | TGTTGGGAGAGTCTAAGATTGTATAAGCACCAATA | 35 |
| 28[90] | 0[70] | 28[90] | TCCAACAGGTCAGGAACCGTCGAGTCCACTATTAA | 35 |
| 18[405] | 24[399] | 18[405] | TTAAGACCAAAGACTTATTCAAGAGGCTCGGATAAAGTTTCCAAGAGGC | 49 |
| 9[399] | 4[385] | 9[399] | TTATCATAATAGTGCTTCTGTAAGGTTATCTAAAATATCTTT | 42 |
| 24[342] | 4[322] | 24[342] | CTACGTTAATAAAAGATTAGTGAGGATTTAGAAGT | 35 |
| 4[419] | 20[413] | 4[419] | GAAAGGAATTGAGGAAATCGTTACCAGGGAGACTCCTCAAGAAATATTG | 49 |
| 20[132] | 27[132] | 20[132] | GAACCGCCCAGAGCGACGTTACTCCAAACAGAGGGCCAATAC | 42 |
| 7[238] | 23[251] | 7[238] | AATCGCGCCCATGTATTTACCGTTCCAG | 28 |
| 22[272] | 9[265] | 22[272] | TTTTCAGAATTACCAAATATATTTTAGTGACGACG | 35 |
| 14[97] | 21[90] | 14[97] | CAGCTCATTTTTTAAAATATTCAGGTCAGAGAAAGTTTTTAGCAAGGCA | 49 |
| 3[91] | 27[99] | 3[91] | CCTTATAGACCATACTAAAGTAC | 23 |
| 7[385] | 20[392] | 7[385] | AGTGAATCGAGAGGACATGAAAGTATTATTAAAGG | 35 |
| 7[175] | 23[188] | 7[175] | TACCTTTGACAGCCGGCCTTGATATTCA | 28 |
| 0[125] | 1[118] | 0[125] | TCGGAACCCGATAGGGTTGAGGAACCATCACCCAA | 35 |
| 26[419] | 2[385] | 26[419] | TGTCGAAAGTGCCAAGCATCACCTTGCTGAACCTATTACCGCCAGCCAT | 49 |
| 24[146] | 7[139] | 24[146] | AAAAGAACTTTGACTTGTTTGGATTATAGTAGATT | 35 |
| 8[104] | 18[91] | 8[104] | GAAGCATAAAGTGTTAGAGGAATAAAAA | 28 |
| 22[209] | 9[202] | 22[209] | CAAACTAGGATTCGGATAAATAAGGCGTGCATTTT | 35 |
| 24[188] | 4[175] | 24[188] | TACCAGAAGCGGGATCCTGATTATCAGA | 28 |
| 20[230] | 5[237] | 20[230] | ACTGTAGTAAAGTACCGACAAATTTAATGTTACAAATTTTGCTACGCCA | 49 |
| 20[293] | 5[300] | 20[293] | TGAAACCTAATGCAGAACGCGAAGACAAACAAACAGACAACTATCACGC | 49 |
| 20[286] | 24[273] | 20[286] | ATCGATAGCAGCACGATGATAACCCTCACTGAGGCATTTAGG | 42 |
| 20[272] | 5[279] | 20[272] | CGTAATCACAATAAACAACATCTTTTTCTGAGCAATGCCCGAACCGAGT | 49 |
| 5[196] | 25[209] | 5[196] | AGGAGGCTCATAACCTTAAACAGCTTGA | 28 |
| 20[181] | 24[168] | 20[181] | GTTTGCCATCTTTTGACGATTCTCATAGTTTATCAAAAAACC | 42 |
| 24[167] | 4[154] | 24[167] | AAAATAGTGCTTTCAATTCATCAATATA | 28 |
| 20[167] | 5[174] | 20[167] | CATAATCATATTTAACAACGCATCATAAGTAACAGTGATGGCCTCGTTA | 49 |
| 5[322] | 25[335] | 5[322] | CTTCTTTCGAACTAAGCAGCGAAAGACA | 28 |
| 20[209] | 5[216] | 20[209] | CATTTTCCGAGCCAGTAATAAACCGTGTCCTGATTACCAGAAAGGGATT | 49 |
| 5[175] | 25[188] | 5[175] | GAATCAGCGACGATGCTTGCTTTCGAGG | 28 |
| 5[301] | 25[314] | 5[301] | AAATTAATTACAGGTTTTGCGGGATCGT | 28 |
| 24[230] | 4[217] | 24[230] | ATTACGAGGAACGGGGAACAAAGAAACC | 28 |
| 24[272] | 4[259] | 24[272] | AATACCATGAGGCCACGTTATTAATTTT | 28 |
| 20[335] | 5[342] | 20[335] | CAGTAGCAACAAGAAAAATAAGGTTATAATTTGAATACATTTAATAACA | 49 |
| 5[343] | 22[350] | 5[343] | TCACTTGGAAAAATAACGAGGGTAGCAAGTATCAC | 35 |
| 20[202] | 24[189] | 20[202] | GGTCATAGCCCCCTCAAACAATGTAGCATGAATTTCCTCGTT | 42 |
| 20[251] | 5[258] | 20[251] | CAAGTTTAGTAATTCTGTCCATAATTTCGAATTATAAAAGTTGTTTTTA | 49 |
| 20[188] | 5[195] | 20[188] | TATTAGCTAATTTAGGCAGAGTAAATAAGGAGAAAATCATATGCTAAAC | 49 |
| 20[307] | 24[294] | 20[307] | GGAAACGTCACCAATAATAAGACCGCCAAGGCCGCTAGAAAG | 42 |
| 20[314] | 5[321] | 20[314] | CAAGGCCATCAACAATAGATATGCTGATTTAATTAATTAGACAGCAATA | 49 |
| 24[293] | 4[280] | 24[293] | ATTCATCTCTGTCCCGTATTAAATCCTT | 28 |
| 0[426] | 1[441] | pass0[426] | GCGAACTTTGAATGGCTATTAGTTTTTTT | 29 |
| 20[438] | 23[438] | pass20[438] | TTTTTTTGGAGGGAAGGTAGAAGGATTAGGATTTTTTT | 38 |
| 25[48] | 25[83] | pass25[48] | TTTTTTGACCATTAGATACAGAGAATAGAAAGGAAC | 36 |
| 2[132] | 1[147] | pass2[132] | AAAGGAACCCCCGACACTAAATTTTTGGGGTCGAGGTTTTTTT | 43 |
| 1[339] | 28[331] | pass1[339] | TTATAAAAGGGTAATCTTGACAATTTTTTT | 30 |
| 9[350] | 8[332] | pass9[350] | TCCTAATAGAAGGCTTATCCGGTATTTTTTTT | 32 |
| 5[54] | 24[50] | pass5[54] | TTTTGGTCCACGCTGGTTTGCACCTGCGAACGAGTAGATTTAGTTTTTTT | 50 |
| 18[156] | 11[154] | pass18[156] | TTTTTAACATTATGACCGTTTCCTGTTTTTTT | 32 |
| 2[439] | 5[439] | pass2[439] | TTTTCAAATGAAACTGGTCAGTTTTT | 26 |
| 2[154] | 25[146] | pass2[154] | TTTAGCCGGCGAACGTGGCGAGATGGTTGGTTTTGCAAAAAGGCTCCAAA | 50 |
| 1[350] | 0[338] | pass1[350] | ACATTCTAGTCACACGACCAGTATTT | 26 |
| 7[51] | 4[51] | pass7[51] | TTTTTTTGTTTTTCTTTTCGTTGCAGCAAGCTTTTTTT | 38 |
| 9[52] | 6[52] | pass9[52] | TTTTTTACATTAATTGCGGGCGCCAGGGTGTTTTTT | 36 |
| 11[52] | 8[52] | pass11[52] | TTTTTTCAGTGCCAAGCTGTGAGCTAACTCTTTTTT | 36 |
| 11[332] | 19[356] | pass11[332] | TTTTTTCTAAGAACGCGATGGCAACATAGCTATCTTACC | 39 |
| 11[364] | 19[377] | pass11[364] | CCTCCCGTAGAAAATTTTTAAGAAAAGT | 28 |
| 26[154] | 3[160] | pass26[154] | TTTTTTATATTCATTCTTGACGGGGAATTTTTTTTT | 36 |
| 2[356] | 26[333] | pass2[356] | CCTACATTTTGACGCTTTTTTTTTCAATCGTCTCTTTAATCATTGTTTTT | 50 |
| 0[439] | 3[439] | pass0[439] | TTTTCTTTAATGCAGCCAGCAGTTTT | 26 |
| 29[332] | 3[356] | pass29[332] | TTTTTTGAACCGGATATTCATTACCATTTCAAGAAATGG | 39 |
| 28[448] | 29[448] | pass28[448] | TTTTTTCAATCATAAGGGAACCACGAGGCGCAGACGGTTTTTTT | 44 |
| 21[48] | 16[56] | pass21[48] | TTTTTTGTAGCATTACAATGCCTGTTTTTTTTTTAGTAATGTGACAAGAG | 50 |
| 18[356] | 10[331] | pass18[356] | ATAAAGGGGCGTTTATCCCAATCCAAATAAGAATTTTTTT | 40 |
| 16[438] | 19[438] | pass16[438] | TTTTTTTTTTACTGAACACACGCAATAATAATTTTTTT | 38 |
| 11[133] | 19[158] | pass11[133] | CATAGCTCTGTAATTCTAGCTGATTTTTTTTTTTTTTTTT | 40 |
| 22[139] | 8[133] | pass22[139] | CTTTCCACGCCGCCAGCATTGCAGAGCCAGGGCTTGAAATTGTTATCCG | 49 |
| 22[437] | 25[438] | pass22[437] | TTTTTTTTAGCGGGGTTTATACGTAATGCCTTTTTTT | 37 |
| 21[326] | 25[342] | pass21[326] | TTTTTCGCAAAGACACCAAATCACGTATAAAAGGAGGTGCATCGG | 45 |
| 22[76] | 20[55] | pass22[76] | CAGCGGAGTTTTCGCAAATGGTCTTTTTTTTTTAATAACCTGCTAATAGT | 50 |
| 0[147] | 27[153] | pass0[147] | TTTTTTGCCGTAAAGTTTAGAGGAATCCCTGCGGAATCGTCATAATTTTT | 50 |
| 1[55] | 29[90] | pass1[55] | TTTCCAACGTCAAAGGGCGAAAATTAGAGCATTTTTGCGGTCTTTACCCT | 50 |
| 14[126] | 18[105] | pass14[126] | TTTTTTTTCGCATTAAATTTTTTAATATTTGAGAGAAATCACCATTTCAA | 50 |
| 11[126] | 5[132] | pass11[126] | TCATGGTCTCACAACAACGCTATACAAAAAATTGCCTTCTGAGCGTACT | 49 |
| 3[52] | 0[51] | pass3[52] | TTTTTTCTGTTTGATGGTAGAACGTGGACTTTTTTTT | 37 |
| 26[69] | 28[48] | pass26[69] | AGAGCTTAAGAGGTAGTACCTTTAATTGCTTTTTTT | 36 |
| 19[325] | 18[325] | pass19[325] | TTTTTTAATGAAATAGCAATATAAAAGAAATTTTTT | 36 |
| 3[70] | 2[51] | pass3[70] | GGTTCCGAAATCGGGTAGCGGTCCCCAGCAGGCGAAAATCTTTTTTT | 47 |
| 17[13] | 15[48] | pass17[13] | TTTTTTTTTTCAATCATATGTACCCCGGTCTGTAGCACAACCCGTCGGAT | 50 |
| 28[154] | 29[154] | pass28[154] | TTTTTTAGACTTCAAATATCGCTAAGAGGAAGCCCGAATTTTTT | 44 |
| 16[146] | 21[132] | pass16[146] | TTTTTTAAATTAATGCCGGAGCAACCGTACTTTTGCATAAAG | 42 |
| 4[441] | 7[452] | pass4[441] | TTTTTTTGGCAAATCTTTCCCTTAGAATCCTTGATTTTTTT | 41 |
| 29[48] | 27[69] | pass29[48] | TTTTTTCCTTTTGATAATTGCTGATTTTTTTTTTATATAATGCTGTAGCT | 50 |
| 6[453] | 9[443] | pass6[453] | TTTTTTTTAAACATAGCGATAGCTTAGAAGTACCGCTTTTTTTT | 44 |
| 26[438] | 28[413] | pass26[438] | TTTTTTTCCTGATAAATTGAGCCGGAGAACTGACCAACTT | 40 |
| 24[438] | 27[437] | pass24[438] | TTTTTTTACTACGAAGGCATTTGTATCATCGTTTTTT | 37 |
| 20[139] | 21[157] | pass20[139] | ACCACCGCTAAATCGGTTGTACCAAATTTTTT | 32 |
| 16[419] | 11[441] | pass16[419] | CCTGAACTTAGACGGGAGAATCTTACCAACCCAGCTACAATTTTTTTTTT | 50 |
| 18[438] | 21[438] | pass18[438] | TTTTTTTCGGAATACCCAACAACCGATTGAGTTTTTTT | 38 |
| 8[442] | 8[420] | pass8[442] | TTTTTTTACTCATCGAGAACAAG | 23 |
| 10[132] | 17[126] | pass10[132] | TTTTTGATGTGCTGCAAGGCGCAGCTGGCTATTTTTTGTTAAAATTTTTT | 50 |
| 13[332] | 16[322] | pass13[332] | TTTTTTACGATTTTTTGTAATAATAAGAGCAAGAAACTTTTTTTTT | 46 |
| 27[332] | 5[356] | pass27[332] | TTTTTTTGAATTACCTTATGCGATTTTGGGAACCTGAGT | 39 |
| 18[118] | 13[134] | pass18[118] | AGCCTTTATCAATATGATATTAGGGTAGCGAAAGGGGTTTTTTT | 44 |
| 20[265] | 24[252] | ref_verb20[265] | AGTAGCGACAGAATTAAGCGTAAGCCCACATAACCCTAATGC | 42 |
| 24[251] | 4[238] | ref_verb24[251] | AGATACAGAGAAGTTGAGTAACATTATC | 28 |
| 20[223] | 24[210] | ref_verb20[223] | CGCGTTTTCATCGGAAGCCAGACCAGTATACCGATTAAGAGC | 42 |
| 24[209] | 4[196] | ref_verb24[209] | AACACTACGATTAAGGAGCGGAATTATC | 28 |
| 20[244] | 24[231] | ref_verb20[244] | GCCTTTAGCGTCAGTCTCTGAACCGTAATGACAACAAAAGGA | 42 |
| 5[259] | 25[272] | ref_verb5[259] | TAATCAGCATTCAAGATATATTCGGTCG | 28 |
| 5[280] | 25[293] | ref_verb5[280] | AAAAGAGAGTTGAGTTGCAGGGAGTTAA | 28 |
| 5[238] | 25[251] | ref_verb5[238] | GAATCCTTAACGCCAACCATCGCCCACG | 28 |
| 5[217] | 25[230] | ref_verb5[217] | TTAGACAGGCATAGAGTTGCGCCGACAA | 28 |
| 5[259] | 25[272] | hant_verb5[259] | CGCCGACGTGATAGAATGTTTTTAATCAGCATTCAAGATATATTCGGTCG | 50 |
| 20[265] | 24[252] | hant_verb5[280] | GCAATCCAGTGTTGGTCATTTTAAAAGAGAGTTGAGTTGCAGGGAGTTAA | 50 |
| 24[251] | 4[238] | hant_verb5[238] | TTGGGAACTCGTGTCTTTTTTTGAATCCTTAACGCCAACCATCGCCCACG | 50 |
| 20[223] | 24[210] | hant_verb5[217] | TGATGTCTGCAGTCGCTGTTTTTTAGACAGGCATAGAGTTGCGCCGACAA | 50 |
| 5[280] | 25[293] | hant_verb20[265] | AGTAGCGACAGAATTAAGCGTAAGCCCACATAACCCTAATGCTTTTCCACACAGTTGCATCGTA | 64 |
| 5[238] | 25[251] | hant_verb24[251] | AGCCGTCGTGGCGAGCCCTTTTAGATACAGAGAAGTTGAGTAACATTATC | 50 |
| 5[217] | 25[230] | hant_verb20[223] | CGCGTTTTCATCGGAAGCCAGACCAGTATACCGATTAAGAGCTTTTAATTCGTCTCCGATCAGC | 64 |
| 24[209] | 4[196] | hant_verb24[209] | GAACTTGGCCTCTAATTCTTTTAACACTACGATTAAGGAGCGGAATTATC | 50 |
| 20[244] | 24[231] | hant_verb20[244] | GCCTTTAGCGTCAGTCTCTGAACCGTAATGACAACAAAAGGATTTTATAGTACAGCTTCAAGCA | 64 |
| 20[230] | 5[237] | fluoro20[230] | ACTGTAGTAAAGTACCGACAAATTTAATGTTACAAATTTTGCTACGCCA-Cyanine3 | 49 |
| 20[272] | 5[279] | fluoro20[272] | CGTAATCACAATAAACAACATCTTTTTCTGAGCAATGCCCGAACCGAGT-Cyanine3 | 49 |
| 20[209] | 5[216] | fluoro20[209] | CATTTTCCGAGCCAGTAATAAACCGTGTCCTGATTACCAGAAAGGGATT-Cyanine3 | 49 |
| 20[251] | 5[258] | fluoro20[251] | CAAGTTTAGTAATTCTGTCCATAATTTCGAATTATAAAAGTTGTTTTTA-Cyanine3 | 49 |
| 16[48] | 12[19] | paint16[48] | GAACGGTAATCGTAAGGCAAAGGCACCGCTTCTGGTGTTTTTTTTTATACATCTA | 55 |
| 15[13] | 14[14] | paint15[13] | TTTTTTTAAATGTGAGCGAGTACAGCTTTCATCAACATTTTTTTTATACATCTA | 54 |
| 13[18] | 16[13] | paint13[18] | TTTTTTTTCCGGAAACCAAACTAGCATGTTTTTTTTTTTTTATACATCTA | 50 |
| 16[48] | 12[19] | Thiol16[48] | GAACGGTAATCGTAAGGCAAAGGCACCGCTTCTGGTGTTTTTTTTTGAGAGAGAGAGAGAGAGAGA | 55 |
| 15[13] | 14[14] | Thiol15[13] | TTTTTTTAAATGTGAGCGAGTACAGCTTTCATCAACATTTTTTTTGAGAGAGAGAGAGAGAGAGA | 54 |
| 13[18] | 16[13] | Thiol13[18] | TTTTTTTTCCGGAAACCAAACTAGCATGTTTTTTTTTTTTTGAGAGAGAGAGAGAGAGAGA | 50 |

Dumbbell 2

| Start | End | Oligoname | Sequence | Length |
| --- | --- | --- | --- | --- |
| 8[440] | 17[510] | 8[440] | GCCACTACTCATTCAGTGAATTGGTTTAATCCTGTGCCTTTTCAACTTT | 49 |
| 6[398] | 9[384] | 6[398] | ACCCGTCGGATTCTCCGTTAATCTTGCTAAAACACTCATTTTTG | 44 |
| 13[91] | 15[111] | 13[91] | CCAGCTACGCCCAACGCTGAGAAGAGTCTGAGTTAACTGAACACCCTGA | 49 |
| 8[426] | 6[406] | 8[426] | ACCAACCCAGCATCTTCAGCGGAGTGAGACAGCCCAACATTAAATGTGA | 49 |
| 1[392] | 5[384] | 1[392] | GCGGGGACGAGCCGGAAATCATCCGCTTCTAATCTATCGCCAGCTG | 46 |
| 11[154] | 7[167] | 11[154] | AAGAGAAGTTCCAGCACCGGAACTAGCACACATATAA | 37 |
| 1[343] | 8[343] | 1[343] | GTCGGGATTCCAGTACTGTTGGTGTAGAGGTGTACCGCGAAA | 42 |
| 1[427] | 15[363] | 1[427] | GGTGGTTGCAGGCGTTTCCCAATCAGAAAGATCTAATTAGCAAAATTAA | 49 |
| 0[370] | 3[384] | 0[370] | CCCGAGATCGGGCCTCTTCGCTATTATTACGCTCCGCATTTCACATA | 47 |
| 3[147] | 5[160] | 3[147] | ACATCGTCACCCGTTAGAATCAGAGGAAGAACACGTAAAA | 40 |
| 16[349] | 14[329] | 16[349] | CAAAGGCTATCAGGTACCCCGAAAATCAGGTCTTTCAAACTCTATTTTC | 49 |
| 17[42] | 11[97] | 17[42] | AAGTTACCACTTAGTATTAATTAATTTTAGTTAATGCCCCCT | 42 |
| 4[398] | 0[406] | 4[398] | AAATAACCTTCTCCGAACTCACATAGAGGCGGTTTGCTGGTGGT | 44 |
| 13[301] | 13[293] | 13[301] | CGAGCTTAGCAAAGCGGATTGCATCAAAATCGCGT | 35 |
| 14[111] | 17[125] | 14[111] | TTTGCCAATCCTGAATCTTACTCATCGTAATTTATCAAAATC | 42 |
| 5[252] | 2[245] | 5[252] | CTGATTGTTTGGGGTCGAGGTAGTGAGGCGCCAGC | 35 |
| 16[335] | 13[349] | 16[335] | TCATTGCATCATACAGGCAAGTTAGCTACAACAGGTCAGGAT | 42 |
| 14[328] | 12[308] | 14[328] | ATTTGGGCCAATAACTGAGAGTCTGGAGAACTAGCCTATTATAGTCAGA | 49 |
| 2[181] | 0[161] | 2[181] | ATTAAACCACACTCGTATTAAATCCTGAAATTGCGTGGCGAGAAAGGAAG | 50 |
| 11[407] | 7[426] | 11[407] | CCACAGAATAGAATCAGCAGCGAAAGATAAAACGCAAATCAACGTAAC | 48 |
| 0[328] | 8[315] | 0[328] | CACTATTATTCGCCCCGTGCATTTGAAAGATTTGTATCATCG | 42 |
| 12[363] | 15[349] | 12[363] | AACGAGAATGACCATAGAGAGGGTCAATAACCTGTGCAAAGA | 42 |
| 8[202] | 6[182] | 8[202] | CCATTTGGGAATTACCGCCTCCCAGAATGGAAAGCTCAGTACAAATTAA | 49 |
| 2[447] | 17[335] | 2[447] | TGTTTCCTCACCAGTGAGACGGGTCCACAAACGACTCATATG | 42 |
| 9[147] | 7[139] | 9[147] | AAAATTAAGCGTCATACGAGGCTGAAGTACATAAATCAATATAAACGTAG | 50 |
| 12[412] | 14[420] | 12[412] | GTCATAAGTTAATATTTTGTTTCAATATATGACCCTGTAATACATATAA | 49 |
| 15[441] | 14[448] | 15[441] | GCCTTTACGGTGTCATGTTTTAAATATGCAGCAGACTAATACTAAAGTA | 49 |
| 17[399] | 15[391] | 17[399] | TGTAAACATATTCATTGAATCTTGCGGAAACGAGTAGATTTAAAGCTAA | 49 |
| 5[329] | 1[342] | 5[329] | ATTCAGGCTGCCATCTCGTCGGTGGGCATGAGCTACTTTCCA | 42 |
| 2[118] | 0[98] | 2[118] | GACATTCATTAGTCAACAAAGAAACCACGGATTATCGCTGCGCGTAACC | 49 |
| 14[321] | 4[455] | 14[321] | GCGCGAGAACCAGACCGGAAGACCCTGAATGTCAAGGCCAGTATTCATG | 49 |
| 4[328] | 1[314] | 4[328] | CTGTAAGTTGGCAAAAGCGCCAAAGAACGTGGACTTTGATTA | 42 |
| 1[455] | 5[447] | 1[455] | GGCAACAATCATGGTCATAGCCGTGGTGCGCACGACTTAAGTCGTTGTA | 49 |
| 8[251] | 10[259] | 8[251] | TGACGGAACAAAAGGGCGACAGCGCAGAACTCAGGAGGTTTAATTGACA | 49 |
| 10[97] | 6[105] | 10[97] | ACGGGGTCGGCATTTTCGGTCCGTAATCATGATTAAGACTCCCTGTAAA | 49 |
| 4[139] | 2[119] | 4[139] | AGTAACATTATCATGAAGGGTCGCTGGCAAGTGTATGACGAGTAAAAGG | 49 |
| 17[70] | 13[90] | 17[70] | TAGCGATAGCTTAGAGCAAGAGCGCATTAGACGGGAGCCATATTTGCAC | 49 |
| 17[203] | 15[216] | 17[203] | CAATCGCTTCTGACACGCTCAACAGTAG | 28 |
| 16[391] | 13[377] | 16[391] | ATAAATTAATGCCGTGTATAAAATGCTTTAAACAGCCTTTTG | 42 |
| 8[300] | 6[287] | 8[300] | TCGAAATGCCGACATGCTTTCGAGGTGACCACCCTATCGGCC | 42 |
| 8[370] | 6[350] | 8[370] | CCCCCAGCGATTAGGAGTACGTTGAAAATCTCGGAACCCCACGTTG | 46 |
| 9[343] | 6[357] | 9[343] | GCTGAGGCTTGCAGTACCAAGAGACCAGGCGCATAGGGATAGGT | 44 |
| 13[483] | 8[427] | 13[483] | ACGAGGCATAGTAAGCGAGAGGCGTCTGAGCCAGCAAAGCTGCGAAGGC | 49 |
| 15[189] | 5[104] | 15[189] | CCAGTATGGCATTTGATAAGTCCTGAACATCGGCTGCAAATCATTGTTT | 49 |
| 11[279] | 7[300] | 11[279] | CAGAACCGATTTCTTATACCGATAGTTGCCCGCGACAGACGGTCAATCAT | 50 |
| 5[203] | 1[216] | 5[203] | TATACAGAGTATTATGAGGCGGTCAGTAATACCTAGGTACGC | 42 |
| 9[315] | 6[308] | 9[315] | CGCCCACAATTGTATCGGTTTTCAGAGCGTTTGAG | 35 |
| 8[188] | 11[202] | 8[188] | GAGCCAGCCACGGAATAAGTTGAAAACACAGGCGGATAAGTG | 42 |
| 1[217] | 8[217] | 1[217] | CAGAATCACCCTAAACCTTTTCAAAAGAAAATTCATATCACC | 42 |
| 5[224] | 1[237] | 5[224] | ACATCGGTAATACACGCCTGCAACAGTGACAGGAAGTGTTTT | 42 |
| 0[384] | 4[364] | 0[384] | AAATCAAAAGAATAGTTAATGAAAGTGTAAAGGCTGAGCCCTGGA | 45 |
| 9[105] | 17[62] | 9[105] | ATAGCCCGGTAATAAGTTTTAGCCTATTTCGTCGCAATCCTT | 42 |
| 14[83] | 12[63] | 14[83] | TTATTTATCCCAATCAGGGAAAACAATGAAATAGCGAAAACAAAGGCTT | 49 |
| 12[510] | 14[483] | 12[510] | TAAAAACCAAAATAGAGCAACTAGAAAGATTCATCAGTTGAG | 42 |
| 2[349] | 0[329] | 2[349] | TAATGAGCGAATATCCGACAGTGCGGCCCTGCGCATTGGAACAAGAGTC | 49 |
| 0[181] | 3[195] | 0[181] | CGGCGAACGTAGATTTTCAGGTTTTCGACACAGCAGAAGATAAA | 44 |
| 6[426] | 9[440] | 6[426] | TTTCATCTCATAGTTAGCGTACAACAGTGGAACGAGGGTAGC | 42 |
| 9[301] | 6[315] | 9[301] | ATGACAACAACCATCCTGATACCGAACTGACCAACTCTGCCA | 42 |
| 5[112] | 1[132] | 5[112] | ACTTCTGAATAATGTTTGCGGTTTAATGCGCGAACCCAGTAACACGTAT | 49 |
| 11[105] | 8[119] | 11[105] | TCGGAACCTATTATGTGTACTCCTTATTAGCGTTTTGAAACC | 42 |
| 15[371] | 17[391] | 15[371] | AGCCTCAGAGCATAGTTTGACATAAGAGGTCATTTCCCCTCAGCAAATA | 49 |
| 2[272] | 4[252] | 2[272] | TAATATCCAGAACAAATGAAAGCACTAACAACTAA | 35 |
| 2[195] | 7[202] | 2[195] | ATCGTCTAAAGGGATTTTAGATTAGAGCAGATGAAACATCAATATTTTG | 49 |
| 11[161] | 8[147] | 11[161] | GGATTAGGCATTTGAATTACCTTTTTTGGTGGCACATTACCATTAGC | 47 |
| 7[119] | 10[133] | 7[119] | GCAGTATGTTAGCATGTGAGTCATGAAAGTATTAAATGGCTT | 42 |
| 2[223] | 4[210] | 2[223] | CATGGAATTAACACTTTGAGGATTTAGA | 28 |
| 11[357] | 7[377] | 11[357] | ATGTACCGTAACACTTTTTTCTAAAGGCCGCCTTTGAGACCTTCA | 45 |
| 9[133] | 11[153] | 9[133] | TTTCATAATCAAGGCCAAAATACATACATAAAAATGGAAACGACTCCTC | 49 |
| 1[161] | 4[147] | 1[161] | GCGGGATTGGCAGATCCATTAAACGTTATTAATTTTA | 37 |
| 2[244] | 4[231] | 2[244] | CATTGCACCACGCTGAGCCGTCAATAGA | 28 |
| 8[139] | 10[119] | 8[139] | GGAAACGTCACCAAGCCATCTTTGATGATACAGGA | 35 |
| 7[231] | 9[251] | 7[231] | TACCAGCTTCAATTCCGGAATAGGTGTAGTCAGACCACCCTCAGAGCCG | 49 |
| 13[420] | 15[440] | 13[420] | AATATAATGCTGTAAGCGTCCGCATTAAATTTTTGCCGGAGAGGGAGAA | 49 |
| 9[259] | 11[278] | 9[259] | AACCACCACCAGAGCCGCTTGAAACAGCCGCCAGCGTACCGCCACCCT | 48 |
| 16[216] | 13[209] | 16[216] | TTTCATCAAGACAAGAAACCAATCAATAAAGAAAA | 35 |
| 11[119] | 8[105] | 11[119] | TCTGAAAGAATAACCTTGCTTTTATTACATCGATAGCAGCAC | 42 |
| 4[363] | 2[350] | 4[363] | GTGACTCCGATCGGTGAGGGTTGAGTGTTGAACCTGTGGGTGCC | 44 |
| 15[91] | 13[83] | 15[91] | AGAATTAAGCCCAATAATAAGATTAAGATAGCAAGCAAATCATTGCTAT | 49 |
| 12[132] | 16[112] | 12[132] | AGCCGTTTTTATTTCAACGCTTTTCCAGAGCCTAAACAAAGTTAACCCA | 49 |
| 12[419] | 16[399] | 12[419] | CGGAATCATTGCTGCAGTTGATTCCCAAAAACATTGATATTCAACCGTT | 49 |
| 10[202] | 6[210] | 10[202] | ATTAAAGCCTCAGAGCCGCCAACTTGAGTCACAATCAATAGAAGATGAT | 49 |
| 15[448] | 13[440] | 15[448] | TTTCAACAAGGGTGAGAAAGGTTAAATCTGTTTAGACTGGATGCTCAAC | 49 |
| 5[147] | 3[139] | 5[147] | AATTATTTGAGCGAAAGGAGCGGGCTTTCCTAGTCACACGATGATAGC | 48 |
| 11[210] | 7[230] | 11[210] | GAGGGTTGATATAATTCACAAAACCGCCACCCTCAGGTGAATTATGGTT | 49 |
| 16[62] | 12[70] | 16[62] | AATAGCTGAATAACATAAAAACCAAATATAAATCAAGATTAGGATATAG | 49 |
| 5[287] | 1[300] | 5[287] | CACCGCTAGGAAGGGAACCTCAAATATCGAAGAACGTAGCAA | 42 |
| 4[202] | 2[182] | 4[202] | GACTTTACAAACAATAACGTCTTGACGGGGAAAGCGCCGATTGAAATGG | 49 |
| 1[301] | 8[301] | 1[301] | TACTTCTCCAACGTCCGGAAAGGGACGAAAGGGAAAATTGTG | 42 |
| 10[384] | 7[370] | 10[384] | TGCGAATAATAATGAGTTTCGCGGCGGATTGACCGTAATGGCTGGCT | 47 |
| 1[98] | 5[90] | 1[98] | GGGCGCGCAGAGATAGAACCCAGACAATAGCGGAATTATCATTCAATAT | 49 |
| 3[469] | 0[455] | 3[469] | AAAGACGCTAAGTGGTTGTGAGCCAAGCGAGTTGCAGCAAGC | 42 |
| 7[329] | 10[343] | 7[329] | GAGGACAGATGAACTGGGCGCTAGCAAGCCCAATACAAAAAA | 42 |
| 17[140] | 13[160] | 17[140] | CTACCTTTAAACACATTACTAGAAAAAGAAAGGTAACATGTTCAGCTAA | 49 |
| 12[489] | 16[469] | 12[489] | GCTTTTGCAAAAGAAGGAATTATTTAGGAATACCATTTTAGAAATGTGT | 49 |
| 17[469] | 15[499] | 17[469] | GAACGCCATCAAAACCTGAGTACCCTCATATATTTTACGTTGGGA | 45 |
| 16[111] | 14[91] | 16[111] | CAAGAATAATAGTGAGGAATCATTACCGCAATTTTGTTACAAAATAAAC | 49 |
| 16[174] | 14[154] | 16[174] | GTGTGATGGTTATAAACGGGTATTAAACTGCAGAAATAAAGTACCGACA | 49 |
| 3[406] | 0[392] | 3[406] | TGACCTCGACAATGTCCCGCCAGCAAGGCGATTCCGAAATCGGCAAAA | 48 |
| 14[377] | 17[363] | 14[377] | CATTAGATACATTTGCAATAATAGCTATTTTTGAGAAGCCCC | 42 |
| 2[314] | 4[294] | 2[314] | CACTTGCCTGAGTAAAACCCTGTTGAAAGGAATTG | 35 |
| 8[398] | 11[406] | 8[398] | AATACAACAAGAACCGGATATTGCGAGTAACATGTAGCATT | 41 |
| 7[168] | 9[188] | 7[168] | AAGAAACGAACAATTTATTAGCGGGGTTTTGCGCAGTCCACCACCGGAA | 49 |
| 11[315] | 8[329] | 11[315] | CACCACCCTCATTTAGCCTTTGCATAACCGATATACAACGGA | 42 |
| 2[384] | 5[398] | 2[384] | AGCATAATCGGCCAACGCTCCCTTATGCGAAAGGGGGATGTGCT | 44 |
| 4[426] | 2[406] | 4[426] | ATTGTCACCAGGGTAAAATCCTGTTTGAGTATTGGCACAATTCCACACA | 49 |
| 8[237] | 6[224] | 8[237] | CATTAAAGAGCCACGATTGGCCTTGATAGTATAGCACCTGAG | 42 |
| 4[104] | 0[112] | 4[104] | CAGAAGGATTTTTGAATGGCTTGGCCAATACTATGGTTGCTTGCGGTCA | 49 |
| 16[461] | 12[469] | 16[461] | GATTCAAGCAAGGATAAAAATCATTCAATACATAACGCCAAAAGTTTTG | 49 |
| 12[160] | 14[168] | 12[160] | CAAGTACTCCGGCTTAGGTTGAAATAAGAGTATCATATGCGTAGAGAAT | 49 |
| 8[342] | 6[329] | 8[342] | CAAAGTATTCGGTCAAGGCTCCAAAAGGTCAGGGAATCGTAA | 42 |
| 5[308] | 3[328] | 5[308] | CCAGGCAATCAACACAATCAATATCTGGTCAGCAA | 35 |
| 7[406] | 11[398] | 7[406] | CATTACCAAAGAGGCAAAAGACCCAGGAACAACTAAACAACGCC | 44 |
| 14[209] | 17[195] | 14[209] | GTAATTTAGGCAGAAAAGCCACTAAATTTAATGGTTGCTGAT | 42 |
| 12[195] | 16[175] | 12[195] | GTCTTTCCTTATCAAACAATATCGAGCCAGTAATATATACAAACCGACC | 49 |
| 8[216] | 6[203] | 8[216] | GTCACCGCCCTCAGACAAATAAATCCTCCCGTCGAGAAACAA | 42 |
| 13[168] | 15[188] | 13[168] | CGCGCCTGTTTATCTTCCAAGTAACTATATGTAAATTGAAATATTCTTA | 49 |
| 1[238] | 8[238] | 1[238] | TATAATCGCCGTAAAATAACGTATTCATGCCAAAGAATTATT | 42 |
| 5[266] | 1[272] | 5[266] | TACCACCAGCCAGCTCAATCACCCAAATAGTCTGT | 35 |
| 17[392] | 13[412] | 17[392] | TTTAAATCTAGCTGATCGGTTGTACCAATTCTGCGTGGCTTAGAGCTTA | 49 |
| 9[441] | 10[448] | 9[441] | AACGGCTTGCTAAACAACTTTACGATCTAAAGTTTTGTCGTCGGGATTT | 49 |
| 12[349] | 3[447] | 12[349] | TAAATCAGTTGATAGTCACGAGTCCTTACAGGGCTTAAGCTA | 42 |
| 2[328] | 2[329] | 2[328] | TTAATTGCGAACATGTAATTTGCGCTCACTGCCCGACTCACA | 42 |
| 7[378] | 9[398] | 7[378] | TCAAGAGGGGAACAAATCACCAGTACAAACTAGGAATCGGGATCGTC | 47 |
| 14[146] | 17[139] | 14[146] | AAGTAATTCTGTCCAGGCGTCAACGAACGACGACAACAAGCATGAGAGA | 49 |
| 16[468] | 17[461] | 16[468] | AGGTAAACCAATAGCCAGAGGGGGTAATAGTAAAAAGCTCATTTTTTAA | 49 |
| 1[133] | 3[146] | 1[133] | AACGTGCGCTAGGGTAGAACCTACCATATCAAAAAGTTTGCCTAAA | 46 |
| 0[447] | 4[427] | 0[447] | GCTGGTTTGCCCCATTTCTTTTGTGTGAAATTGTTGAGTAAAGTGCTGA | 49 |
| 5[406] | 1[426] | 5[406] | TAAGTTGGGTAACGACCTTATCTGGTTGGTGTAATATCCGCTGCGCCAG | 49 |
| 5[350] | 1[370] | 5[350] | GGAAGGGTATGATAAGGGGCCTTGAATCGCCTGCGTGCCAGCTGCA | 46 |
| 14[440] | 12[420] | 14[440] | TGGAAGTTTCATTCCTTTTGCCAGTCAAATCACCAAAAATTCAATACTG | 49 |
| 14[363] | 16[371] | 14[363] | CGCAAATTACCTTTAATTGCTTTCAGAAAAAAACAGGAAGATGAGAGGG | 49 |
| 12[62] | 16[42] | 12[62] | ATCCGGTGAAGCCTAGAAACGATTTTTTACAGAGAATCTTACCGAAGCC | 49 |
| 5[161] | 1[181] | 5[161] | CAGAAATAAATTGCCCGAAAATACCGAACGTTTACAGCTAAACAGGAG | 48 |
| 16[509] | 17[489] | 16[509] | TCATTATACCAGTCAGGAAATGCAATGATAATTC | 34 |
| 5[294] | 2[287] | 5[294] | TCTGGTGCAAAGGGCGAAAAAAACCGTTTCAAACT | 35 |
| 8[83] | 9[90] | 8[83] | CAAGTTTGGAATACCCAAAAGAACTGGCAGTAGCGACAGAATTTTTCAT | 49 |
| 6[300] | 10[301] | 6[300] | CGACAGTCAGAACCGCCACCCATCAGCT | 28 |
| 15[154] | 13[146] | 15[154] | CCTGTTTGCGTTAAATAAGAATTTAACCCGCACTCATCGAGAATAAACA | 49 |
| 5[231] | 2[224] | 5[231] | GAGAAACAGCACTAAATCGGACTGAGAAAAACGCT | 35 |
| 5[210] | 3[202] | 5[210] | TAACAGTAGGGAGCCCCCGATCAGGAACCATTTTGACGCTCAACAGAGG | 49 |
| 6[181] | 10[161] | 6[181] | TTACATTTCAAAGACACAAAATCACCAGCAGAGCTCTGAATTTACC | 46 |
| 16[125] | 17[132] | 16[125] | AGAGAGACAGAGGGTAATTGAGCTCATACGGAAGCTAATATCATAGGTC | 49 |
| 10[473] | 17[534] | pass10[473] | TTTAATGAATTTTCTGTATTTTCCAGAATTGGGGTGAATTACCTTTTTTT | 50 |
| 9[448] | 7[479] | pass9[448] | ACAGAGGCTTTGAGTAAACGGGCCCTGACGAGAAACACCATTTTTT | 46 |
| 14[216] | 13[233] | pass14[216] | CCAACATATAATATCCCATCCTAATTTTTTT | 31 |
| 6[83] | 14[42] | pass6[83] | CCGAAGGCCGAACACTTTTTAAAATAGCAGCCTTTGTTTAAC | 42 |
| 5[64] | 17[239] | pass5[64] | TTTTAGATGATGGGAGAAAACTTTTTTTT | 29 |
| 14[531] | 16[510] | pass14[531] | TTTTTTAACGGAACAACATTACGTTAATGAACTGGC | 36 |
| 12[232] | 4[64] | pass12[232] | TTTTTACGAGCATGTAAGAACGCCAATTCACATATTCCTGATTATCTTTT | 50 |
| 15[500] | 13[528] | pass15[500] | AGAAAAATCTATTACAGGACTATCATAACCCTCGTTTTTTTTT | 43 |
| 16[239] | 15[237] | pass16[239] | TTTTTCAAATATATTTTTGAGAATCTTTTT | 30 |
| 13[42] | 12[25] | pass13[42] | TTGCGGGAGGTTTTATTCTAAGAACGCGAGGCGTTTTAGCTTTTT | 45 |
| 13[308] | 13[300] | pass13[308] | CAAAGCGCTGAAAAGGTGGCATCAATTTTTTAATT | 35 |
| 15[289] | 16[294] | pass15[289] | TTTTTTTTCTACTAATAGTGAGAATCGATTTTT | 33 |
| 6[479] | 11[475] | pass6[479] | TTTTTTGAACGAGTAGTAACGTTAGTATTTTT | 32 |
| 16[34] | 6[62] | pass16[34] | AGAAAAGTATTTTTTTTAGCAGATAGAAACCGAGGAAATTT | 41 |
| 14[236] | 16[217] | pass14[236] | TTTTGCCATATTTAACAACGGGCTTAATAGTTAA | 34 |
| 12[286] | 13[286] | pass12[286] | AAGATTAAGAGGAAGCTTTTTTTTTTCCGAAAGACTTCAAAT | 42 |
| 7[62] | 9[83] | pass7[62] | TTTCGCAATAATAACGCCTTTAGCTTTTTTTTTTGTCAGACTGTAGCGCG | 50 |
| 10[90] | 11[83] | pass10[90] | CAGTGCCTTGAGTAACAGTTTTTTTTTTTGCCCGTATAAAC | 41 |
| 16[534] | 15[531] | pass16[534] | TTTTTATGCGATTTTAAAAAACGAACTTTTT | 31 |
| 17[294] | 5[493] | pass17[294] | TTTTTTGAACGGTAGGAGAAGCCAGGTTTTTT | 32 |
| 4[490] | 2[469] | pass4[490] | TTTGTGGATGTTCTTGAGGATCCCTTTTTTTTTTCGGGTACCGAGCTCGA | 50 |
| 9[469] | 9[468] | pass9[469] | GACTTTTTCTTTTTTTTTTATGAGGAAGTTTCCATGACTAAA | 42 |
| 0[483] | 15[321] | pass0[483] | TTTCTGGCCCTGAGATTTCTCAATCGTAACAAACAAAGTAGCATTAACAT | 50 |
| 12[525] | 8[441] | pass12[525] | TTTACCAGACGACGAAATCATTCTTGAGAAAGGCTTGTAAAATACGTAAT | 50 |
| 0[90] | 5[97] | pass0[90] | CCGCCGCGCTTTTTTTTTTTTAATGCGCCGCTACAACCACACAATCCTG | 49 |
| 13[23] | 14[28] | pass13[23] | TTTTTTTGAACCTCCCGACGTCAAAAATTTTTT | 33 |
| 3[455] | 1[486] | pass3[455] | CTTGTTACCTCGATATTCGTAGCTGATTGCCCTTCACCGCTTTTTT | 46 |
| 2[90] | 3[90] | pass2[90] | TTCTGACCTGAAAGCGTTTTTTTTTTTAAGAATACGTGGCAC | 42 |
| 0[286] | 8[280] | hant_verb0[286] | CCGTCTATTTCCGGTCAGGAAGAGGCGCCTGCTCCTTTTTTACTTGTTGCGTTCCTAGCCGCTATAT | 67 |
| 11[245] | 3[251] | hant_verb11[245] | TCACCGTGGCGAATGATTCGCTAGATTAGAGAGCCTTTTTTTTGTCTCTTTGCCGACTAATGTGAAC | 67 |
| 0[265] | 8[259] | hant_verb0[265] | CAAGTTTCTTTGAACAAAATCTTCAACCGGGAAGGTTTTTTGCCTCGTGAGACCATTGCGAATACCA | 67 |
| 2[286] | 3[272] | hant_verb2[286] | ATCGGCCCCTTGCTTTATCTAAAATATCTTTAGGAAATCTAATTTTTTTACGCGAGATAAACTGCTAGGAAACC | 74 |
| 3[252] | 1[265] | hant_verb3[252] | GGATAAAACTGCCCTGGTGAGCGCCATTTTTTAGCAGCAATATTACCCACCGAGTAAAAG | 60 |
| 8[258] | 10[245] | hant_verb8[258] | TCTACTATTCGAGGCCGTTCGTTAATTTTTTTTAAATATCCACCAGGGAGGTTGAGGCAG | 60 |
| 3[273] | 1[286] | hant_verb3[273] | AACCACACCATAGCGATTTATCGGAGTTTTTTAGCATCATTGCTGGCCATCACGCAAATT | 60 |
| 8[279] | 6[266] | hant_verb8[279] | CGCCTCGGAATACGGTATGAGCAGGCTTTTTTATGTTACTTAGGGAGATTGAGCCGGAACGATCGCACTAGTTA | 74 |
|  |  | verdr0[286] | ATATAGCGGCTAGGAACGCAACAAGT | 26 |
|  |  | verdr11[245] | GTTCACATTAGTCGGCAAAGAGACAA | 26 |
|  |  | verdr0[265] | TGGTATTCGCAATGGTCTCACGAGGC | 26 |
|  |  | verdr2[286] | GGTTTCCTAGCAGTTTATCTCGCGTA | 26 |
|  |  | verdr3[252] | TGGCGCTCACCAGGGCAGTTTTATCC | 26 |
|  |  | verdr8[258] | ATTAACGAACGGCCTCGAATAGTAGA | 26 |
|  |  | verdr3[273] | CTCCGATAAATCGCTATGGTGTGGTT | 26 |
|  |  | verdr8[279] | GCCTGCTCATACCGTATTCCGAGGCG | 26 |

Stopper module

| Start | End | Oligoname | Sequence | Length |
| --- | --- | --- | --- | --- |
| 1[357] | 26[364] | 1[357] | GCATTGACAGGAGGGAGGTGCCCCCCGATTTAGAGAGGAGCGGGCGCTA | 49 |
| 1[413] | 26[392] | 1[413] | TAAATCCAAGTTTTAACGGGGCATGAAAGCTGGTTTGGCGAGAAAGGAA | 49 |
| 0[426] | 29[440] | 0[426] | TGGTAATTCATTAAAGCCAGAGACTCCATGATGGTGGTTCCG | 42 |
| 0[440] | 24[434] | 0[440] | ATACAGGAGTGTACAGACTCCGAGTTGCCGGCCAACGCGCGGGCCGGAA | 49 |
| 3[385] | 24[392] | 3[385] | TCGGAACGGGAAGATTTCCAGTCGGGAAAGCTAAC | 35 |
| 3[392] | 28[406] | 3[392] | CTATTATTCTGAAATCAGTGCTATTCACAAACAAAAAAACCG | 42 |
| 2[377] | 4[364] | 2[377] | AGAGCCACCACCGGTTCGGTCATAGCCCAAAAAAAAGGCTCC | 42 |
| 5[91] | 25[104] | 5[91] | TGGTTTAAACTATCCATTTTGACGCTCA | 28 |
| 5[133] | 25[146] | 5[133] | TTGAGGGTTTGATTCAGATTCACCAGTC | 28 |
| 5[154] | 25[167] | 5[154] | TTGACGGTAACCGTCAGTAATAAAAGGG | 28 |
| 5[343] | 7[356] | 5[343] | TAGCGCGGCTACAGCAGTATTAACACCGTCGTATTCTTTGAA | 42 |
| 5[378] | 1[391] | 5[378] | CCTTATTCGGAACCGCCTATTCCCGTATAGGTCAGACGATTG | 42 |
| 5[385] | 7[398] | 5[385] | AGCGTTTTCACATTCAGCAAATGAAAAATGAGGATAGGCTGG | 42 |
| 5[392] | 22[413] | 5[392] | GCCATCTTTCGCCACCCTCAGGTGCCTACATCACCTTGCTGAGCCGTCA | 49 |
| 5[413] | 23[419] | 5[413] | AACCGCCAAAGGAATTGCGAATCATCAAATAGATAGTGCATCTGCCAGT | 49 |
| 5[434] | 23[440] | 5[434] | CACCCTCTGAGAATAGAAAGGAGAACCGACTAATAGACGACGACAGTAT | 49 |
| 5[511] | 25[524] | 5[511] | TCACCAGGCTCGAAGTAAAACGACGGCC | 28 |
| 5[532] | 25[540] | 5[532] | CCTGTAGTAGAGGAAGCTTGCAT | 23 |
| 4[356] | 23[356] | 4[356] | GCCTTTAATTGTATTGACCAAAAATCCTAACAAACGGCGGAT | 42 |
| 4[405] | 22[385] | 4[405] | TAATAATTTTTTCACGTTGAAGGCGCATTTAGAAG | 35 |
| 7[70] | 23[83] | 7[70] | TTATTACCCTTTTATGCTTTGAATACCA | 28 |
| 7[91] | 23[104] | 7[91] | AACGTAGGATGAATAAATCGCGCAGAGG | 28 |
| 7[133] | 24[126] | 7[133] | ATGCCACACAGAAATAAAGAAACATTGGAGTAATA | 35 |
| 7[175] | 23[188] | 7[175] | GAATACAGAAGGGTTTGTAAACGTTAAT | 28 |
| 7[196] | 23[209] | 7[196] | TTTGACCGGATTATTAAAATTCGCATTA | 28 |
| 7[217] | 23[230] | 7[217] | CAAGCGCTCAATATGTTAAATCAGCTCA | 28 |
| 7[238] | 23[251] | 7[238] | CGGAGATTTATCAGACCAATAGGAACGC | 28 |
| 7[259] | 23[272] | 7[259] | GATAAATGAATTATAATAATTCGCGTCT | 28 |
| 7[280] | 23[293] | 7[280] | CGACCTGAAGAAACCTGTAGCCAGCTTT | 28 |
| 7[301] | 23[314] | 7[301] | GCCGGAAACATTATATTAAATGTGAGCG | 28 |
| 7[322] | 23[335] | 7[322] | TCAATCATTAATTTACCCGTCGGATTCT | 28 |
| 7[357] | 3[377] | 7[357] | AGAGGACAAAAGGACGGCATTAACCGCCCCTCAGAGCCACATGCCCCCT | 49 |
| 7[364] | 25[384] | 7[364] | AGATGAAAACAATTAATGGGATAGGTCACGTTGGTTATTAGAGAGCCAG | 49 |
| 7[371] | 21[370] | 7[371] | CGGTGTATAAGAAACGATTTTCTAAATTGCCTGAGAACCCTC | 42 |
| 7[413] | 7[405] | 7[413] | GAGTAATCAGTTACAAAATAAACAGCCACTGACCT | 35 |
| 7[476] | 5[496] | 7[476] | GTGAATAAGGCTTGATTTTCTAACCCATGTACCGT | 35 |
| 7[490] | 23[503] | 7[490] | CCCTGACGCTGCAATGCCGGAAACCAGG | 28 |
| 7[511] | 23[524] | 7[511] | CGAGTAGCAGCTGGCCATTCGCCATTCA | 28 |
| 6[356] | 21[356] | 6[356] | ACGTCAAAAATGAATAGTTAAAGATTCACGCAAGGATAAAAA | 42 |
| 6[398] | 20[371] | 6[398] | TATTATTTATCCCAACCGACCTAAAATGTTTAGAAATGCAAT | 42 |
| 6[503] | 20[469] | 6[503] | ACCAGTCAGGACGTTGGGACCCAGCTACAATTTTATAGTTGCCATAACC | 49 |
| 9[112] | 24[105] | 9[112] | GCTTCTGATGAAACAAACATCCGAATTACAGGTTTATCGTCTAGTAGAA | 49 |
| 9[119] | 5[132] | 9[119] | TAAATCGTCGCTATCAGATAGATACGTAGGACTAACAACCGA | 42 |
| 9[133] | 20[119] | 9[133] | TAATTAATGATAATCACAAAAGAAGATG | 28 |
| 9[371] | 11[377] | 9[371] | TAATGGTAATCATAATTACTAGCCAACGCTTCGGC | 35 |
| 9[420] | 3[426] | 9[420] | TTAGCGAAATTTGCCTTGACAAACAACTACCCTCAATCACCGGAGGCTG | 49 |
| 9[441] | 3[447] | 9[441] | GGGAGGTACGAGCGATTACCCAGCGGAGAGAGCCAAGTATAGAAGGATT | 49 |
| 11[378] | 4[378] | 11[378] | TGTCTTTACACCGGTTGAAATATCCAAACAGACCAAATCTCC | 42 |
| 11[385] | 13[405] | 11[385] | CCTTATCCATAAAGATTAACATCCAATAGAACGAGTATAAAGTACCGAC | 49 |
| 10[398] | 19[384] | 10[398] | ACGAGCATGTAGAAAAGAGAATAGATTTAGTTTGATAGTAGC | 42 |
| 10[405] | 19[419] | 10[405] | CTAATTTGAACGGGTATTAAATTAAGCACAGGCAAGGCAAAG | 42 |
| 13[350] | 6[357] | 13[350] | TGTAATTTAGGCAGGGCTTAAGTATAAAGAAAAAGTTCTGACTTGTTTA | 49 |
| 13[364] | 21[363] | 13[364] | AGGCATTATACATTTCGCAAATGGCATCCATTATGTTTTTAG | 42 |
| 13[427] | 16[434] | 13[427] | CAGACGATTTCATTCCATATAAACCAGACCGGAAGCAAACTCCTGGAAG | 49 |
| 13[434] | 8[441] | 13[434] | CGACAATAAACAACCAACAATAAGCCGTCCAATAG | 35 |
| 16[377] | 10[364] | 16[377] | CCATTAGTTCGAGCCAGTAATACCAATCAATAACAACAGTAG | 42 |
| 16[412] | 23[405] | 16[412] | ATTCCCAATTCTGCAATCATAATAAAGCTGCGGAAAGAGGGGCGTAACC | 49 |
| 16[419] | 8[420] | 16[419] | ACAGTTGAAAGTAATTCTGTCCAAGAAACCGCACTATAGAAG | 42 |
| 16[482] | 18[462] | 16[482] | TGTAGCTCAACATGTTTTAAATACCTTTCCCGAAA | 35 |
| 16[503] | 20[483] | 16[503] | CTTAATTGCTGAATATAATGCATAAGAGATCAAAACAGGTCTAAGAGCA | 49 |
| 19[441] | 25[447] | 19[441] | CAACAGGCGTTTTATTTAAACAAAACCACGGCCTCTAGGAGCATCAATA | 49 |
| 18[384] | 19[377] | 18[384] | CTAAATCGGTTGTACCAAAAAAATTCTACTAATAG | 35 |
| 18[433] | 13[412] | 18[433] | CTTCAAAGCGAATTAGCAAAACCAAGTAAATAATATCCCATCAAAAGGT | 49 |
| 21[371] | 11[398] | 21[371] | ATATATTTTACTGGATAGCGTCCAATACCTCAGAGATTCCAA | 42 |
| 21[462] | 27[468] | 21[462] | GAATGACCTCGTTTCCAGCCAGAAGGTTAAATCAATCCGCTCGGCGCCA | 49 |
| 21[469] | 19[482] | 21[469] | CATAAATCAAAAATAGATTAAGAGGAAGAATTGCTCCTTTTG | 42 |
| 20[83] | 5[90] | 20[83] | TTTAACAAGTACATAAATCAACAATAATGTTAGCAATATAAAATTCATA | 49 |
| 20[97] | 24[84] | 20[97] | CAAAATTAATTACAAGTTACAATACAGTATACCTAGGCCTTG | 42 |
| 20[104] | 9[97] | 20[104] | AAGAAAAGAGTGAATAACCTTCAGAAGGAAACCGAGGAAACGTATATGT | 49 |
| 20[146] | 5[153] | 20[146] | CCCCGGTTTTTCCCTTAGAATGCCCTTTGCACCAACAACGGCGTAAATA | 49 |
| 20[160] | 24[147] | 20[160] | GTCAATCATATGTAAGGAAGATATTTGCACACGACTGTAGCA | 42 |
| 20[328] | 25[321] | 20[328] | GCCGGAGACAGTCAAGTAACATAAAAGTGCAGAAG | 35 |
| 20[363] | 26[357] | 20[363] | TAATGTGTGACCGTCGACAACCCTGCAAGCGCTTAGCTGCGCGGGCGCT | 49 |
| 20[412] | 22[420] | 20[412] | TTTTGCCTCGTCATAAATATTCATTGAATTTTGCATTGAGGGGATTAGA | 49 |
| 20[419] | 8[385] | 20[419] | AAAGAAGAGGCGTTGCTTATCCGGTATTCTAGGCGTTAAATAAGAATAA | 49 |
| 20[433] | 10[427] | 20[433] | GAGAGGCTCCCCCTCAAATGCATTCGAGAACAAGCAGATAAGTCCTGAA | 49 |
| 20[440] | 11[433] | 20[440] | AAATAGCGACTTGCCAAGCAAATCAGATCATCGAG | 35 |
| 20[503] | 5[510] | 20[503] | TTACGAGGTTAATAAAACGAATCATTATACCAGAATTTCCAGAGTTTCG | 49 |
| 20[517] | 22[504] | 20[517] | AACGCCAAAAGGAACAAAGCGCGAAAGG | 28 |
| 20[524] | 5[531] | 20[524] | GATACATAACAACATTATTACTATGCGAGGCTTGAACGATCTTACAACG | 49 |
| 20[538] | 24[525] | 20[538] | ATTCAACTAATGCAGGCTGCGCTTCGCTAGTGCCATCCCCGG | 42 |
| 23[112] | 23[146] | 23[112] | TTCATTTCAATTACCTGAGGAAAAGCCCCAAAAAC | 35 |
| 23[385] | 9[412] | 23[385] | GTAGATGGGCGCATGTAATAGGTGTGATAAATAAAGAACGCG | 42 |
| 23[483] | 20[476] | 23[483] | CTTCTGGGGCGATTAAGTTGGAATTGAGGCTTTCCGGCACCGACACTAT | 49 |
| 22[83] | 20[68] | 22[83] | AACAGTAGCAGTATAACGGAATACCCAATGGAAACATTTCATTT | 44 |
| 22[104] | 22[112] | 22[104] | AACGTCAAAAATACATTTTCCATTAAACAGATTTT | 35 |
| 22[125] | 6[112] | 22[125] | ATTGCGTGGGTAAACCGAACAAAGTTAC | 28 |
| 22[146] | 6[133] | 22[146] | ACGTAAATACGAAGTTAAGAAAAGTAAG | 28 |
| 22[167] | 9[160] | 22[167] | TACCATACGAAAGAGCTATCTTACCGAACCTTGAA | 35 |
| 22[188] | 9[181] | 22[188] | AATAATGCTAAAACAGAAACAATGAAATTTAGATT | 35 |
| 22[209] | 9[202] | 22[209] | ATTGTTTCCCAGCGTTAAGCCCAATAATAGTCAAT | 35 |
| 22[230] | 9[223] | 22[230] | CAATTCAGAAACAAAGATAACCCACAAGAATCATA | 35 |
| 22[251] | 9[244] | 22[251] | TTCCTGATTGTATCAATTGAGCGCTAATCCTTTTT | 35 |
| 22[272] | 9[265] | 22[272] | AGGAGCGTGTGTCGCCCTGAACAAAGTCGTTGGGT | 35 |
| 22[293] | 9[286] | 22[293] | CGGAACACTCCATGGACGGGAGAATTAATAAATGC | 35 |
| 22[314] | 9[307] | 22[314] | TTGAGTACGAGGCGTAAAAACAGGGAAGTCGCAAG | 35 |
| 22[335] | 9[328] | 22[335] | AACGTTATAAGGGAGCCTTTACAGAGAGAAAACTT | 35 |
| 22[405] | 24[413] | 22[405] | ATACATTTCTAAAGATGAGTGACCTGTCGTGCCAGCTGCATTGCCTGGG | 49 |
| 22[454] | 6[462] | 22[454] | ATATCTTAGGAAGATCGCACTACCAGACCCTTAAATCAAGATTCCTGAA | 49 |
| 22[524] | 9[517] | 22[524] | ATTACGCTAAATTGTTTTAAGAACTGGCCTAACGG | 35 |
| 22[541] | 9[538] | 22[541] | GTGTAATTTCATTGTGAATTACCTAGGTAGA | 31 |
| 25[420] | 28[413] | 25[420] | ACCTCAAGTGTAAAAATGAATAGCAAGCTCCTGTTACGTCAAAGGGCGA | 49 |
| 25[427] | 9[433] | 25[427] | ATATCAAACCCTCAACTAACAGATATTCTCTTTCCAGAGCCTACCTCCC | 49 |
| 25[504] | 4[490] | 25[504] | CGACGTTTTCGTAATCATGGTAACACTGACGTTAGTAAATGA | 42 |
| 24[83] | 4[70] | 24[83] | CTGGTAAATAGAAAAGAAACGCAAAGAC | 28 |
| 24[104] | 25[125] | 24[104] | GAACTCACCAGCGCCAAAGACAAAAGGGTTGCCTGGAAATGGATTATTT | 49 |
| 24[125] | 4[91] | 24[125] | ACATCACCGACATTAGACTTTTTCATGAGGAAGACATAAAGGTGGCAAC | 49 |
| 24[146] | 4[133] | 24[146] | ATACTTCAGGGAAGTACAGAGGCTTTGA | 28 |
| 24[321] | 4[301] | 24[321] | GACGAGCACGTATAGCGACAGTTTCTTAAACAGCT | 35 |
| 24[342] | 4[322] | 24[342] | GGCGCGTACTATGGTTAGCGTTCAGCTTGCTTTCG | 35 |
| 24[384] | 22[371] | 24[384] | AATTGCGTTGCGACACCCGCCCAGTGCCACGCTGACTTTACA | 42 |
| 24[447] | 1[440] | 24[447] | CATACGAGGAGAGGGCCTGGCAAATCGGTATTAAAGAACGTGATGGAAA | 49 |
| 24[454] | 6[448] | 24[454] | CACACAATCTGGTCAGTTGGCATCTAAACGTAACATCTTACCAACGCTA | 49 |
| 24[489] | 7[475] | 24[489] | GTTTCCTGTGTGAACAATAGGGTATGGGATTTTGCTCATTCA | 42 |
| 24[496] | 7[503] | 24[496] | CATAGCTGGTTTTCCCAGTCAGGGATGTGAGAAAC | 35 |
| 24[524] | 4[511] | 24[524] | GTACCGATACAAACAAAGTTTTGTCGTC | 28 |
| 27[364] | 29[384] | 27[364] | GTAACCACCCTCACTGCCCGCAAGCGAACTTGACG | 35 |
| 27[448] | 7[454] | 27[448] | CGGTTTGCGTATTGACAATTCCATTTTCCTTTCAACAGTTTCAAATCAA | 49 |
| 26[419] | 2[385] | 26[419] | GGTCCACGTATTAATACTCAGGAGGTTTAGTACTCATAATCAAAATCAC | 49 |
| 26[440] | 24[427] | 26[440] | CCTGAGATCAAGAGCCCGGAATAGGTGTGAACCGCGCATAAA | 42 |
| 26[468] | 28[448] | 26[468] | AACAGCTAATCAAACAGTTTGGAACAAGAGTCCAC | 35 |
| 29[448] | 5[454] | 29[448] | CAAAATCCCTTATAGATTGCCGCGGGGTAGAGGGTTGATATACCACCCT | 49 |
| 28[384] | 0[357] | 28[384] | AGTTTTTTGGGGTCTTGAGGCAAACAGTTACACCCTCAGAGC | 42 |
| 28[391] | 29[419] | 28[391] | CAAATCAGGGAAAGCCGGCGAACGTGCCCCAGCAGGCGAAAA | 42 |
| 28[405] | 0[385] | 28[405] | TCTATCACATCACCGCCTTGACTTGAGTAACAGTG | 35 |
| 5[175] | 25[188] | 5[175] | AGGTGAAGAGTCTGGGCCAACAGAGATA | 28 |
| 5[196] | 25[209] | 5[196] | GACTTGACAGTGAGTCTGACCTGAAAGC | 28 |
| 5[301] | 25[314] | 5[301] | ATCAGTAACGTGCTCGAACGAACCACCA | 28 |
| 5[322] | 25[335] | 5[322] | TTTGCCTTTGCTTTATAAAACAGAGGTG | 28 |
| 7[154] | 23[167] | 7[154] | CCTAAAATCAAAATTTGTATAAGCAAAT | 28 |
| 20[167] | 5[174] | 20[167] | CTAGCATAACATAGCGATAGCAGCAATAGGCAAAAACAGCATTCATTAA | 49 |
| 20[181] | 24[168] | 20[181] | CGGTAATCGTAAAAATTTAAATAGAACCACATTCTTCCATCA | 42 |
| 20[188] | 5[195] | 20[188] | CGATGAAAAGACGCTGAGAAGAAGAGCAACTCATCCGTCACCCGTCACC | 49 |
| 20[202] | 24[189] | 20[202] | GCAAACAAGAGAATATTTTGTACTTCTGGAACCCTGCCACCG | 42 |
| 20[286] | 24[273] | 20[286] | TCTAGCTGATAAATGGCCTTCCACCAGAAGCCCTAGGAGCTA | 42 |
| 20[293] | 5[300] | 20[293] | CAACCGTTGATGCAAATCCAACGCATTATTACTTATGATACCCACCGTA | 49 |
| 20[307] | 24[294] | 20[307] | ATCAATATGATATTCATCAACCATTTTGAAAATACTTCCTCG | 42 |
| 20[314] | 5[321] | 20[314] | AATCACCACAAAGAACGCGAGAATAACACAGACGGAGGTGAAAATCAAG | 49 |
| 20[335] | 5[342] | 20[335] | GAGAAAGTTTCAAATATATTTAATAGCAACCGAACCGGTTTACAGACTG | 49 |
| 24[167] | 4[154] | 24[167] | CGCAAATAAATTATCGGAACGAGGGTAG | 28 |
| 24[188] | 4[175] | 24[188] | AGTAAAATTATCACCTCAGCAGCGAAAG | 28 |
| 24[230] | 4[217] | 24[230] | CCAGAATGCAAAATGGCTTGCAGGGAGT | 28 |
| 24[272] | 4[259] | 24[272] | AACAGGAACGTCACAACAACCATCGCCC | 28 |
| 24[293] | 4[280] | 24[293] | TTAGAATATAGCAGGATAGTTGCGCCGA | 28 |
| 5[217] | 25[230] | 5[217] | TGATGTCTGCAGTCGCTGTTTAGAGCCACCTGAGATACGTGGCACAGAC | 49 |
| 5[238] | 25[251] | 5[238] | TTGGGAACTCGTGTCTTTTTTAGCACCAACAGGAATTGAATGGCTATTA | 49 |
| 5[259] | 25[272] | 5[259] | CGCCGACGTGATAGAATGTTTGCCGGAAGGCCGATATGCGCGAACTGAT | 49 |
| 5[280] | 25[293] | 5[280] | GCAATCCAGTGTTGGTCATTTACCATCGCAGAGCGAAACATCGCCATTA | 49 |
| 1[338] | 0[338] | passiv1[338] | TTTACCAGAGCCGCCGCCACGCCACCAGAACCACCTTT | 38 |
| 0[447] | 1[469] | passiv0[447] | TTTGATGGCGCAGTCTCTGAATTTACCGTTCCATTT | 36 |
| 0[469] | 26[448] | passiv0[469] | TTTGTAAGCGTCATACATGGCTAGGATTACTTCACC | 36 |
| 2[356] | 3[356] | passiv2[356] | TCCCTCAGAGCCGCCATTTTTTCCCTCAGAACCGCCAC | 38 |
| 2[480] | 25[489] | passiv2[480] | TTTCGGATAAGTGCAAGCCATTGTTACAGTTGAAAGGGTAACGCCAG | 47 |
| 7[462] | 3[480] | passiv7[462] | AAGCTGCTAAACAAAGGGATAGCCGTCGTTTGCTCAGTACCAGGTTT | 47 |
| 9[448] | 11[466] | passiv9[448] | TTTGAAGGACGATAAGTTCAGAAAACGAGACTTCATTCATCGTATTT | 47 |
| 9[476] | 21[510] | passiv9[476] | TATTTTGCAAGAAAAATCTACGCATAGTTTACCCTGACTATTATAGTTT | 49 |
| 8[466] | 18[448] | passiv8[466] | TTTGGAATCATTACCGCGCTTTTATTAATATCG | 33 |
| 11[335] | 16[350] | passiv11[335] | TTTGTTATACAAATTAATACTTGCGAGCTGAAAAGGTGGTCAA | 43 |
| 10[466] | 16[441] | passiv10[466] | TTTGAACGCGCCTGTTTATATGTTCACTAAAGTACGGTGT | 40 |
| 19[448] | 13[466] | passiv19[448] | TCAGGATTAGAGAGTATGCAAGCTAATGCATTT | 33 |
| 18[512] | 19[512] | passiv18[512] | TTTTCAGAAGCAAAGCGGATTGCGTCATTTTTGCGGATGGCTTTTT | 46 |
| 21[328] | 25[342] | passiv21[328] | TTTCCTTTATTTCAAAAAGGGTCCGTGGGTTGCCCGAGGCGGT | 43 |
| 20[356] | 8[335] | passiv20[356] | TAGGTAATTTCATCCCTGTTTAGTATCATATGCTTT | 36 |
| 26[356] | 5[356] | passiv26[356] | GGCAAGTGTTTTTTTAGCGGTCACATGCGCCTTTTCAT | 38 |
| 26[488] | 27[488] | passiv26[488] | TTTTCACCAGTGAGACGGGCGGGTGGTTTTTCTTTTTTTT | 40 |
| 29[357] | 29[356] | passiv29[357] | AAGGGAGCGTAAAGCACTAAATCTTTTTTTTGGAACCCTA | 40 |
| 28[488] | 29[488] | passiv28[488] | TTTTGGGTTGAGTGTTGTTCAGAATAGCCCGAGATATTTT | 40 |
| 20[223] | 24[210] | refverb20[223] | GTCATTGCCTGAGAAATTTTTAATCCTGGTAAGAAAGTGTTT | 42 |
| 20[244] | 24[231] | refverb20[244] | GAGATCTACAAAGGTTTTTTAATGATGGAATATTTCGGTACG | 42 |
| 20[265] | 24[252] | refverb20[265] | GGAGAGGGTAGCTACATCAAACATCATAGTCTTTATAAAGGG | 42 |
| 24[209] | 4[196] | refverb24[209] | TTATAATGCCATTTCGCTTTTGCGGGAT | 28 |
| 24[251] | 4[238] | refverb24[251] | ATTTTAGTTACCATACCGATATATTCGG | 28 |
| 5[217] | 25[230] | refverb5[217] | AGAGCCACCTGAGATACGTGGCACAGAC | 28 |
| 5[238] | 25[251] | refverb5[238] | AGCACCAACAGGAATTGAATGGCTATTA | 28 |
| 5[259] | 25[272] | refverb5[259] | GCCGGAAGGCCGATATGCGCGAACTGAT | 28 |
| 5[280] | 25[293] | refverb5[280] | ACCATCGCAGAGCGAAACATCGCCATTA | 28 |
| 5[217] | 25[230] | verb5[217] | TGATGTCTGCAGTCGCTGTTTAGAGCCACCTGAGATACGTGGCACAGAC | 49 |
| 5[238] | 25[251] | verb5[238] | TTGGGAACTCGTGTCTTTTTTAGCACCAACAGGAATTGAATGGCTATTA | 49 |
| 5[259] | 25[272] | verb5[259] | CGCCGACGTGATAGAATGTTTGCCGGAAGGCCGATATGCGCGAACTGAT | 49 |
| 5[280] | 25[293] | verb5[280] | GCAATCCAGTGTTGGTCATTTACCATCGCAGAGCGAAACATCGCCATTA | 49 |
| 20[223] | 24[210] | verb20[223] | GTCATTGCCTGAGAAATTTTTAATCCTGGTAAGAAAGTGTTTTTTAATTCGTCTCCGATCAGC | 63 |
| 20[244] | 24[231] | verb20[244] | GAGATCTACAAAGGTTTTTTAATGATGGAATATTTCGGTACGTTTATAGTACAGCTTCAAGCA | 63 |
| 20[265] | 24[252] | verb20[265] | GGAGAGGGTAGCTACATCAAACATCATAGTCTTTATAAAGGGTTTCCACACAGTTGCATCGTA | 63 |
| 24[209] | 4[196] | verb24[209] | GAACTTGGCCTCTAATTCTTTTTATAATGCCATTTCGCTTTTGCGGGAT | 49 |
| 24[251] | 4[238] | verb24[251] | AGCCGTCGTGGCGAGCCCTTTATTTTAGTTACCATACCGATATATTCGG | 49 |
| 20[209] | 5[216] | fluoro20[209] | GTCTGGAAGTGAATTTATCAAAATTGAGATTATACTAAAGGCGGGAATT-Cyanine3 | 58 |
| 20[230] | 5[237] | fluoro20[230] | CTATCAGGGTCTGAGAGACTAATCAGAGAGTACAATCGCTGACACCAGT-Cyanine3 | 58 |
| 20[251] | 5[258] | fluoro20[251] | TTTTTGAAACCTCCGGCTTAGAGAGGGTATCGCCTACGCATATAGCAAG-Cyanine3 | 58 |
| 20[272] | 5[279] | fluoro20[272] | TAATGCCTATATAACTATATGCTGAACAAAATCCGCAATGACCAATGAA-Cyanine3 | 58 |
| 13[335] | 16[328] | T_paint13[335] | TTTACAACGCCAACATAACCTGTTTAGCTATATTTTTTTATACATCTA | 48 |
| 19[328] | 18[328] | T_paint19[328] | TTTTTCATTTGGGGCTTGCGGGAGAAGTTTTTATACATCTA | 41 |
| 18[356] | 10[335] | T_paint18[356] | ACCCTGTCTTACCATTGAGAATCGCCATATTTATTTTTATACATCTA | 47 |
| 5[546] | 25[83] | poly5[546] | CAGACGTTTATTTTGTCACAATCATATCCAGAAACGCTCATGGAA | 45 |
| 7[532] | 23[61] | poly7[532] | GATGGTTCGGGCCTCAACTGTTGGGAAGGGAACGGATT | 38 |
| 7[546] | 4[546] | poly7[546] | AACTTGATTAAGACTCCACCACGGAATAAAGCCC | 34 |
| 9[547] | 6[546] | poly9[547] | TCAGTTGACTTTTTTAAAAGAACTGGCATTAATC | 34 |
| 20[67] | 9[546] | poly20[67] | GAATTACGATTTAGGAATACCACAAGATTCA | 31 |
| 23[62] | 22[542] | poly23[62] | CGCCTGATCATCGGGAGAAACAATCGATCG | 30 |
| 25[541] | 24[61] | poly25[541] | GCCTGCAGCCATTGCAACAGGAAAACAATATT | 32 |
| 24[60] | 4[532] | poly24[60] | ACCGCCAGGTCGACTCCATTCCATCATAGTTAGCGTA | 37 |
| 95[777] | 25[83] | zu10hb95[777] | TTCAAGTTTATTTTGTCACAATCATATCCAGAAACGCTCATGGAA | 45 |
| 97[763] | 23[61] | zu10hb97[763] | TCAATATGTAATCAATCCCCGGGTACCGAGAACGGATT | 38 |
| 97[777] | 94[777] | zu10hb97[777] | AACCGGATTAAGACTCCACCACGGAATAAAGCGA | 34 |
| 99[778] | 96[777] | zu10hb99[778] | TATCGGCCCTTTTTTAAAAGAACTGGCATTTCTA | 34 |
| 115[772] | 24[61] | zu10hb115[772] | TGTAAAGGCCATTGCAACAGGAAAACAATATT | 32 |
| 20[67] | 99[777] | zu10hb20[67] | GAATTACTCAGGAAGATCGCACTGACGACAG | 31 |
| 23[62] | 112[773] | zu10hb23[62] | CGCCTGATCATCGGGAGAAACAATCTCGAA | 30 |
| 24[60] | 94[763] | zu10hb24[60] | ACCGCCACCTGGGGTGTTCGAGCACCAGACCGGAAGC | 37 |
| 110[67] | 9[546] | zu10hb110[67] | ACGCAATGATTTAGGAATACCACAAGATTCA | 31 |
| 113[62] | 22[542] | zu10hb113[62] | ATCATAGGAGAGTCAATAGTGAATCGATCG | 30 |
| 114[60] | 4[532] | zu10hb114[60] | GGAGGCCGGTCGACTCCATTCCATCATAGTTAGCGTA | 37 |
| 5[546] | 115[83] | zu10hb5[546] | CAGACAACTTTGAAAGAGGACAGAGAGCGGGGACAGGAACGGTAC | 45 |
| 7[532] | 113[61] | zu10hb7[532] | GATGGTTCGGGCCTCAACTGTTGGGAAGGGTTATCAAA | 38 |
| 7[546] | 4[546] | zu10hb7[546] | AACTTTACTCAGGAGGTACCGAACTGACCAGCCC | 34 |
| 9[547] | 6[546] | zu10hb9[547] | TCAGTTGAAATAACGGAGGTGTATCACCGTAATC | 34 |
| 25[541] | 114[61] | zu10hb25[541] | GCCTGCAGATTAAAGGGATTTTAAGCTAAACA | 32 |

Axle module

| Start | End | Oligoname | Sequence | Length |
| --- | --- | --- | --- | --- |
| 22[230] | 9[223] | 22[230]9[223] | TTACATTGACAGCCCAGTTAATGCCCCCCATATGG | 35 |
| 24[629] | 4[616] | 24[629]4[616] | TTTTCTTCCTCAAAGTACGGTGTCTGGA | 28 |
| 7[658] | 23[671] | 7[658]23[671] | GGATAAACAATTCGTGCTGCAAGGCGAT | 28 |
| 7[91] | 23[104] | 7[91]23[104] | CCGCCACAGCGATACCTCCGGCTTAGGT | 28 |
| 20[181] | 24[168] | 20[181]24[168] | AAGCCCAATAATAAAACTTTTAATATATAATACTTCGTAACC | 42 |
| 7[112] | 23[125] | 7[112]23[125] | CCCTCAGCCTTAGATATAACTATATGTA | 28 |
| 7[742] | 23[755] | 7[742]23[755] | CCGGAGATCCTGTGCCTGCAGGTCGACT | 28 |
| 20[545] | 9[559] | 20[545]9[559] | TATTCTAAAAATCACCTAAATTTTTGTT | 28 |
| 20[601] | 24[588] | 20[601]24[588] | TAGGAATCATTACCAACCAATTTTTGCGGCCAGCATGCCCTT | 42 |
| 9[742] | 23[741] | 9[742]23[741] | AACCGTGCTTCTGGTGCCGGATTGCATG | 28 |
| 5[175] | 25[188] | 5[175]25[188] | CTGCTCACGCTGCGCTTTGATTAGTAAT | 28 |
| 7[133] | 23[146] | 7[133]23[146] | TTTTCAGCGTCGCTATGCAAATCCAATC | 28 |
| 20[629] | 5[636] | 20[629]5[636] | AACAAGCTAGCCAGCTTTCATGGTTGATTGCGGGAAACTAAATGCTTTA | 49 |
| 24[741] | 25[741] | 24[741]25[741] | TGCGTTGCGCTCACTGCCCGCGCACTAACACCGCTCACAATT | 42 |
| 7[154] | 23[167] | 7[154]23[167] | ATAGGAAAATAACCAAAGAACGCGAGAA | 28 |
| 20[566] | 5[573] | 20[566]5[573] | AGATATAAAATCAGCTCATTTCGTTAATTAAAGCCAGTAGATTTTAGAC | 49 |
| 20[118] | 24[105] | 20[118]24[105] | AAGAAAAGTAAGCATGGGTTAATCCTTGTTTATAACGAGCAC | 42 |
| 22[587] | 9[580] | 22[587]9[580] | GAACAAAATAAAGCATTTAAATTGTAAATTTAACC | 35 |
| 20[335] | 5[342] | 20[335]5[342] | GTTTAACTTGAGCCATTTGGGTCTCTGAGTGAGAACGGCTACTACGTTA | 49 |
| 24[461] | 4[448] | 24[461]4[448] | ATAGGGTGCATAGTAACCATCGCCCACG | 28 |
| 22[314] | 9[307] | 22[314]9[307] | CAAAATCCTAAACACATACATGGCTTTTTTAAAGG | 35 |
| 7[217] | 23[230] | 7[217]23[230] | TTCCACATAACAATATGGTTTGAAATAC | 28 |
| 24[650] | 7[643] | 24[650]7[643] | CGTATTGCAGAAAATGTTTTAAATATGCGAAGCCT | 35 |
| 20[370] | 24[357] | 20[370]24[357] | TTATTTATCCCAATGGGCTTATCGGGAGACGACCAACTACGT | 42 |
| 20[685] | 24[672] | 20[685]24[672] | GGCGATCGGTGCGGTAAGTTGTTAGACTATCAACACGGCCAA | 42 |
| 20[671] | 5[678] | 20[671]5[678] | GCCTCTTCGTCGGATTCTCCGGAACGGTAGAACCCTTGCTGAAATCAGG | 49 |
| 7[196] | 23[209] | 7[196]23[209] | CAAACTACTTTTTTTCATCTTCTGACCT | 28 |
| 24[188] | 4[175] | 24[188]4[175] | GCGGTCATTCAGTGCAAGCGCGAAACAA | 28 |
| 24[671] | 4[658] | 24[671]4[658] | CGCGCGGAATCAAAATATAATGCTGTAG | 28 |
| 20[482] | 9[475] | 20[482]9[475] | ATCAAGACGCGTTTTCATCGGCCCTCAGAGCCACCACCCTCAACTGTAG | 49 |
| 5[721] | 4[721] | 5[721]4[721] | TCAAAAAGATTAAGACCTTTAATTGCTC | 28 |
| 20[461] | 5[468] | 20[461]5[468] | GCACCCAGCCTTTAGCGTCAGGAGCCGCATTTCTTTGACAACAAGAGCA | 49 |
| 5[469] | 25[482] | 5[469]25[482] | ACACTATAAGAATAGCGCGAACTGATAG | 28 |
| 7[238] | 23[251] | 7[238]23[251] | TTAGCGTGAAAACAGTGATAAATAAGGC | 28 |
| 22[83] | 20[68] | 22[83]20[68] | TTAAGACCGCCACCTATAGCCCGGAATAATACCCAACCGAGGAA | 44 |
| 24[755] | 4[742] | 24[755]4[742] | TAACTCAACTTCAAAACAGGTCAGGATT | 28 |
| 22[713] | 9[706] | 22[713]9[706] | CGTCAATAATGTGTTTGCCTGAGAGTCTCCGTAAT | 35 |
| 24[587] | 4[574] | 24[587]4[574] | CACCGCCCGTCCAACAATTCTGCGAACG | 28 |
| 5[595] | 25[608] | 5[595]25[608] | GAATCGTAGCTGATGCAAATGAAAAATC | 28 |
| 24[293] | 4[280] | 24[293]4[280] | AAAGGGATTTTAAGATTAAACGGGTAAA | 28 |
| 5[364] | 25[377] | 5[364]25[377] | ACATTATATGGCCCGTAATAAAAGGGAC | 28 |
| 5[511] | 25[524] | 5[511]25[524] | AAATAGCTTGATGGAACGAACCACCAGC | 28 |
| 5[385] | 25[398] | 5[385]25[398] | TTCATCAGGCGAAACCAACAGAGATAGA | 28 |
| 20[734] | 5[741] | 20[734]5[741] | AACCAGGGATGGGCGCATCGTTGAGAGAAGAAAGGAGAGAGTAGGAAGC | 49 |
| 20[167] | 5[174] | 20[167]5[174] | GAGCAAGAAGAAACGCAAAGAAGAGGCTACCGTAAAGTACAAAACAAAG | 49 |
| 5[448] | 25[461] | 5[448]25[461] | TTACGAGTGAGTGTGAATGGCTATTAGT | 28 |
| 22[356] | 9[349] | 22[356]9[349] | AAACAATGAACAACAATGGAAAGCGCAGAATTAGA | 35 |
| 20[769] | 24[756] | 20[769]24[756] | CCAGCCAGCTTTCCCTAGAGGTGGTCATAGCCGGAAGTGAGC | 42 |
| 7[448] | 23[461] | 7[448]23[461] | TGCTTTCAAAATTACGACAAAAGGTAAA | 28 |
| 22[608] | 9[601] | 22[608]9[601] | GAGTAACCAAAAACAGGAAGATTGTATACAAAAAT | 35 |
| 20[139] | 24[126] | 20[139]24[126] | TATCTTACCGAAGCAATGCTGATTAATTGAGTAAACGCGTAC | 42 |
| 20[433] | 24[420] | 20[433]24[420] | TTACCAACGCTAACGAGCCAGAAGAAATAAGAATATCCACTA | 42 |
| 20[524] | 5[531] | 20[524]5[531] | TTTAGCGGTTTGCCATCTTTTCCGGAACTAAATCAAATAACCTTTTGCA | 49 |
| 7[175] | 23[188] | 7[175]23[188] | CACTGAGATAAATCTCAAATATATTTTA | 28 |
| 22[377] | 9[370] | 22[377]9[370] | CAGTACCATAATAAATAAATCCTCATTACAGTAGC | 35 |
| 24[125] | 4[112] | 24[125]4[112] | TATGGTTTCAAGAGCGACCTGCTCCATG | 28 |
| 24[713] | 4[700] | 24[713]4[700] | TCGGGAACAAAGCGTAAGAGGTCATTTT | 28 |
| 24[314] | 4[301] | 24[314]4[301] | AAAGCACACCAGTCCTTTTTCATGAGGA | 28 |
| 5[91] | 25[104] | 5[91]25[104] | ACCAGGCGTGCTTTTCCTGAGAAGTGTT | 28 |
| 22[503] | 9[496] | 22[503]9[496] | TGTTTGGTACTAATCCTCAGAACCGCCACATTTTC | 35 |
| 24[356] | 4[343] | 24[356]4[343] | GAACCATGAACTAAAACGAGGGTAGCAA | 28 |
| 20[587] | 5[594] | 20[587]5[594] | GCGCCCAAATAGGAACGCCATAGCAAATTAAATCGTGATTCCTACTGCG | 49 |
| 7[322] | 23[335] | 7[322]23[335] | ACAGTTTTGAATACAATTCTTACCAGTA | 28 |
| 22[209] | 9[202] | 22[209]9[202] | GAATTACCAACGCCTTCGGAACCTATTATGTCACA | 35 |
| 20[188] | 5[195] | 20[188]5[195] | TTGAGTTGAATAAGTTTATTTTTCTGAAACCAGTAATTATACAATAAGG | 49 |
| 24[566] | 4[553] | 24[566]4[553] | TTGCAGCTAAAATGTTAGTTTGACCATT | 28 |
| 24[272] | 4[259] | 24[272]4[259] | GCTTGACATTGTGAATGCCACTACGAAG | 28 |
| 9[644] | 5[657] | 9[644]5[657] | AAATGTGAGCGAGTCATGTCAAACGCAACTCAACACGAGAAT | 42 |
| 20[125] | 5[132] | 20[125]5[132] | CCTTTTTAGCAAACGTAGAAATTTTGCTACCCTCAAAATCCGTAATCTT | 49 |
| 20[398] | 5[405] | 20[398]5[405] | TTTGCCAGGAAACGTCACCAAGACGATTCAAAAAAAGGCCGCTTTAGGA | 49 |
| 24[377] | 4[364] | 24[377]4[364] | TATCACGTACAGGTAGCAGCGAAAGACA | 28 |
| 7[553] | 23[566] | 7[553]23[566] | GCAAAATATTATCAAAATAATATCCCAT | 28 |
| 24[335] | 4[322] | 24[335]4[322] | TTTTTGGAAAAATCAGAGGCTTTGAGGA | 28 |
| 5[427] | 25[440] | 5[427]25[440] | GATACATACAAGAGCGTGGCACAGACAA | 28 |
| 5[637] | 22[644] | 5[637]22[644] | AACAGTTGGCGCCAATCAAACCCTCAATGTATTAA | 35 |
| 5[196] | 25[209] | 5[196]25[209] | CTTGCCCCGCTGGCCTTGCCTGAGTAGA | 28 |
| 22[146] | 9[139] | 22[146]9[139] | TGTAAATGGATAGCTAGGATTAGCGGGGATACATA | 35 |
| 20[349] | 24[336] | 20[349]24[336] | AGAAACGATTTTTTTAAAGCCTCGCCTGATTGGCAATCAAGT | 42 |
| 20[356] | 5[363] | 20[356]5[363] | CCAAATAGCCAGCAAAATCACAAGCCAGTAAAGGAGCATCGGCGGAACA | 49 |
| 7[343] | 23[356] | 7[343]23[356] | TAGAAAGAACGGATAACGCTCAACAGTA | 28 |
| 22[524] | 9[517] | 22[524]9[517] | ATTCATCCATCCAACGCCTCCCTCAGAGTATTAGC | 35 |
| 22[692] | 9[685] | 22[692]9[685] | AGGATTTTTTTAAAACAAGAGAATCGATTGGGAAC | 35 |
| 24[104] | 4[91] | 24[104]4[91] | GTATAACGCATAGGGCCGGAACGAGGCG | 28 |
| 20[622] | 24[609] | 20[622]24[609] | AAGCCGTTTTTATTCTTTCCTAAAGTTTTAAAGCAGTGAGAC | 42 |
| 5[532] | 25[545] | 5[532]25[545] | AAAGAAGCCCAGCAAAAACAGAGGTGAG | 28 |
| 22[755] | 6[742] | 22[755]6[742] | AGCTGTTCAGTCAAAGGGTAGCTATTTT | 28 |
| 24[489] | 5[489] | 24[489]5[489] | CCCTTATAAATCAACATAACCCTCGTTT | 28 |
| 24[419] | 4[406] | 24[419]4[406] | TTAAAGAATTCAACTTGCAGGGAGTTAA | 28 |
| 20[496] | 25[489] | 20[496]25[489] | TTTTGAAGCCTTAACAATAAATTCTGAACCCTAAA | 35 |
| 7[385] | 23[398] | 7[385]23[398] | ACGTTGATGAATATCAACGCCAACATGT | 28 |
| 24[167] | 4[154] | 24[167]4[154] | ACCACACTCAACGTCGGAGATTTGTATC | 28 |
| 20[727] | 24[714] | 20[727]24[714] | CAAAGCGCCATTCGAAACGACTTAGAGCTTTAGGATTTCCAG | 42 |
| 5[700] | 25[713] | 5[700]25[713] | TCAGAAGACCTGTCTTATCTAAAATATC | 28 |
| 20[454] | 24[441] | 20[454]24[441] | GCTACAATTTTATCAAAGTACTTTGCACTATTTTTTGTTCCA | 42 |
| 5[553] | 22[560] | 5[553]22[560] | GTAATAGAAGCGGTGTATTAACACCGCCGAGCGGA | 35 |
| 7[406] | 23[419] | 7[406]23[419] | AAGGCTCATTTTCAGCAGAGGCATTTTC | 28 |
| 20[755] | 4[756] | 20[755]4[756] | GGCACCGCATCTGCCAGTTTGGCCGGAGATCACCAAAACTCC | 42 |
| 20[559] | 24[546] | 20[559]24[546] | GAAGGCTTATCCGGACAAGAATCATATTGCGGTCACCACGCT | 42 |
| 22[125] | 9[118] | 22[125]9[118] | AATTTTCAGCCACCCAGTACCAGGCGGAGTATGTT | 35 |
| 7[364] | 23[377] | 7[364]23[377] | ATTGCGATTTTACAATTGAGAATCGCCA | 28 |
| 7[511] | 23[524] | 7[511]23[524] | GCATTAAAATATAAAACGCGCCTGTTTA | 28 |
| 22[545] | 5[552] | 22[545]5[552] | CCTGATTAAGGCAAAGAATTAAGATACAAGAGGGG | 35 |
| 7[427] | 23[440] | 7[427]23[440] | AATTGTAAGAAATATAATAAGAGAATAT | 28 |
| 5[679] | 25[692] | 5[679]25[692] | TCTTTACAATGAATGTTGAAAGGAATTG | 28 |
| 22[251] | 9[244] | 22[251]9[244] | ACATCAAAACGATCGAGTAACAGTGCCCAGACAAA | 35 |
| 20[419] | 5[426] | 20[419]5[426] | GAGCGTCATCGATAGCAGCACACAGGAGAGCCTTTCTGAGGCTAATGCA | 49 |
| 20[83] | 5[90] | 20[83]5[90] | GAAGGAAAAAGAACTGGCATGATATAAGCTCAGAACAGACGGTGTACAG | 49 |
| 20[517] | 24[504] | 20[517]24[504] | AACCTCCCGACTTGAATGCAGTCCTGATAATACCGTGGTTCC | 42 |
| 20[160] | 24[147] | 20[160]24[147] | AAACAATGAAATAGGCAAGACTTGCTTCACGCAAAGCTTAAT | 42 |
| 5[322] | 25[335] | 5[322]25[335] | TGGGAAGGGTCGAGAATGGATTATTTAC | 28 |
| 5[406] | 25[419] | 5[406]25[419] | ATACCACACGTGGATGACCTGAAAGCGT | 28 |
| 20[692] | 5[699] | 20[692]5[699] | TGGGAAGAAACGGCGGATTGAGGAGCAATGCAATGTGCGGATATTATAG | 49 |
| 22[272] | 9[265] | 22[272]9[265] | CAAAAGACTTTCCATTTTAACGGGGTCACCGATTG | 35 |
| 22[104] | 9[97] | 22[104]9[97] | AAAACATCCTCAGACGTCGAGAGGGTTGATTAAGA | 35 |
| 24[524] | 4[511] | 24[524]4[511] | ATCCTGTGAGAGGCTGTTTAGCTATATT | 28 |
| 24[608] | 4[595] | 24[608]4[595] | GGGCAACCATAAATTTCCATATAACAGT | 28 |
| 5[343] | 25[356] | 5[343]25[356] | ATAAAACCACCCAAGATTCACCAGTCAC | 28 |
| 5[756] | 25[771] | 5[756]25[771] | ATATCGCGTTTTAACCTAATGAGCATAAAG | 30 |
| 22[293] | 9[286] | 22[293]9[286] | TATTCATAATTTTCCAGGAGTGTACTGGAATATTG | 35 |
| 7[301] | 23[314] | 7[301]23[314] | GATTTTGGCGCAGATAGTATCATATGCG | 28 |
| 24[398] | 4[385] | 24[398]4[385] | GTCAAAGGTTGAGATTTTGCGGGATCGT | 28 |
| 5[301] | 25[314] | 5[301]25[314] | TCATTATTAAATCGTTTTGACGCTCAAT | 28 |
| 24[440] | 4[427] | 24[440]4[427] | GTTTGGAAACGCCAGATATATTCGGTCG | 28 |
| 20[104] | 5[111] | 20[104]5[111] | GATAGCCCTCCTTATTACGCATAAGTGCACCGCCATTACTTACTGGCTG | 49 |
| 20[580] | 24[567] | 20[580]24[567] | ATAGCAAGCAAATCCCTAATTCCAGAAGTGCAACAGAGAGAG | 42 |
| 7[259] | 23[272] | 7[259]23[272] | TTGTCGTAGATGATAAGAATAAACACCG | 28 |
| 20[608] | 5[615] | 20[608]5[615] | TTCATCGAATTCGCGTCTGGCCAAAAACATTATGAAGTTTCAATTCATT | 49 |
| 20[328] | 24[315] | 20[328]24[315] | GTCAAAAATGAAAATTATACACAAGTTACGTCTGAGTGCCGT | 42 |
| 20[307] | 24[294] | 20[307]24[294] | CTTTACAGAGAGAAGCCTGTTGGCGAATACCTACAGAACCCT | 42 |
| 22[335] | 9[328] | 22[335]9[328] | ATTGCTTCAGCGGAATTTACCGTTCCAGCACCGAC | 35 |
| 20[650] | 24[630] | 20[650]24[630] | CTGGCTACCGCACTCATCGAGCGGGTATGCCCGAACTCAAATGGGTGGT | 49 |
| 7[469] | 23[482] | 7[469]23[482] | AAACAGCAGGGTTATGTCCAGACGACGA | 28 |
| 22[482] | 23[503] | 22[482]23[503] | TAATGGATTGATACCGTGGCATCAATTCATTATACCAACATGTTCAGCT | 49 |
| 7[616] | 23[629] | 7[616]23[629] | CCCTGTAAATTTTATATCATTCCAAGAA | 28 |
| 5[616] | 25[629] | 5[616]25[629] | GAATCCCTTCACCATCACCTTGCTGAAC | 28 |
| 22[741] | 23[734] | 22[741]23[734] | TGAAATTGTTATACTAATAGAGGCCAGTGCCAAGC | 35 |
| 22[398] | 9[391] | 22[398]9[391] | CGTCAGAAAATCTCGGCCTTGATATTCACAAGGCC | 35 |
| 20[475] | 24[462] | 20[475]24[462] | TTAGTTGCTATTTTGTAATTCGAACCTACTTTAATGCCCGAG | 42 |
| 7[532] | 23[545] | 7[532]23[545] | TACAGGCATCAGATTAGATAAGTCCTGA | 28 |
| 20[202] | 24[189] | 20[202]24[189] | ATAACCCACAAGAAGTTAATTAATGGAAAACATCAAAGTGTA | 42 |
| 20[286] | 24[273] | 20[286]24[273] | AAAACAGGGAAGCGGAATCATACCTGAGAGGAAAAATTTAGA | 42 |
| 5[658] | 25[671] | 5[658]25[671] | GACCATAGGAGAGGTGGTCAGTTGGCAA | 28 |
| 20[538] | 24[525] | 20[538]24[525] | AGAACGCGAGGCGTTCAACAAGATGGCAAGAAGATGGCGAAA | 42 |
| 22[772] | 9[769] | 22[772]9[769] | TTCGATATTCGCTGATAAATTAATAGGGGAC | 31 |
| 24[692] | 4[679] | 24[692]4[679] | CTGCATTCCTGACTGGCTTAGAGCTTAA | 28 |
| 5[490] | 25[503] | 5[490]25[503] | ACCAGACGCAAAATACATCGCCATTAAA | 28 |
| 24[83] | 4[70] | 24[83]4[70] | AGAATCATGAACGGTCAATCATAAGGGA | 28 |
| 22[167] | 9[160] | 22[167]9[160] | GTGAGTGCCCATGTGAGACTCCTCAAGACATATAA | 35 |
| 7[560] | 9[538] | 7[560]9[538] | TAAGCAAATTTTGTTAAAATTCGCATGGAACCAGAGCCACCACATAATC | 49 |
| 7[280] | 23[293] | 7[280]23[293] | GTAAATGTTCAATTAATTACTAGAAAAA | 28 |
| 5[574] | 25[587] | 5[574]25[587] | TGGATAGTGGCCCTGTGCCACGCTGAGA | 28 |
| 7[644] | 9[643] | 7[644]9[643] | TTATTTCATCATATGTACCCCCAACATT | 28 |
| 24[503] | 4[469] | 24[503]4[469] | GAAATCGGACGATAGGGGCGCGAGCTGAAAAGGATAGTTGCGCCGACAA | 49 |
| 5[133] | 25[146] | 5[133]25[146] | GACAAGATACAGGGAGAGTCTGTCCATC | 28 |
| 7[595] | 23[608] | 7[595]23[608] | GTTGTACATTATCACAATAATCGGCTGT | 28 |
| 20[440] | 5[447] | 20[440]5[447] | CTGAATCAGTAGCGACAGAATCCAGAGCATCAGCTCATAACCAAAGGAA | 49 |
| 20[706] | 24[693] | 20[706]24[693] | GGCTGCGCAACTGTTTTCCCAACATTTGAGGAAGGGTGCCAG | 42 |
| 20[503] | 5[510] | 20[503]5[510] | CGGGAGGGGTCATAGCCCCCTCCGCCACAGTAGTATTCATTTAAAACCA | 49 |
| 7[70] | 23[83] | 7[70]23[83] | TTAGTACGCTGAGATCTGAGAGACTACC | 28 |
| 20[293] | 5[300] | 20[293]5[300] | TAACATAACGGAAATTATTCAGATGATATGTATGGAGTTTCCAACTGGC | 49 |
| 5[742] | 25[755] | 5[742]25[755] | CCGAAAGCATTAATCCACACAACATACG | 28 |
| 5[112] | 25[125] | 5[112]25[125] | ACCTTCAGCTTTGATCAGTGAGGCCACC | 28 |
| 22[629] | 9[622] | 22[629]9[622] | CGTTATTATACTTTAATCAGAAAAGCCCCTTCCTG | 35 |
| 24[545] | 4[532] | 24[545]4[532] | GGTTTGCTTTTGCCTTTCGCAAATGGTC | 28 |
| 20[713] | 5[720] | 20[713]5[720] | CCATTCAGGGATAGGTCACGTCAGGTCAAGGTAAACTTTTGAGATTGCA | 49 |
| 22[440] | 9[433] | 22[440]9[433] | GTAAAACTCGGTTTCGCCGCCAGCATTGCGTAATC | 35 |
| 22[671] | 20[651] | 22[671]20[651] | TTACAAAAATTTTTAATCGTAAAACTAGAACAACCCGCTATTACGCCAG | 49 |
| 20[146] | 5[153] | 20[146]5[153] | CAATAGCCATAAAGGTGGCAAGAAGGATAAGCCCAATCGCCTATTCATT | 49 |
| 20[412] | 24[399] | 20[412]24[399] | TTTCCAGAGCCTAAAATTTAGGGTTTAAACCCTTCCTCCAAC | 42 |
| 22[188] | 9[181] | 22[188]9[181] | ACAGTACTTTCGTCACATGAAAGTATTACACCACG | 35 |
| 20[377] | 5[384] | 20[377]5[384] | AGCCATAACCATTACCATTAGCAAACAATTTTTTCCACCCTCAGAAAGA | 49 |
| 5[154] | 25[167] | 5[154]25[167] | ACCCAAACCGCCGCTTAACCGTTGTAGC | 28 |
| 20[391] | 24[378] | 20[391]24[378] | GTTACAAAATAAACTATTTAAACAGTAAATTCTGGAACCGTC | 42 |
| 24[146] | 4[133] | 24[146]4[133] | GCGCCGCACCGGATGATAAATTGTGTCG | 28 |
| 7[679] | 23[692] | 7[679]23[692] | TCATATAAGAAGTAGGTAACGCCAGGGT | 28 |
| 22[461] | 9[454] | 22[461]9[454] | CCATATCGAGGTGACACCAGAACCACCACAAGTTT | 35 |
| 7[721] | 9[727] | 7[721]9[727] | GATTCAAAAGGGTGTCTACAAAGGCTATTGGTGTA | 35 |
| 20[314] | 5[321] | 20[314]5[321] | TAGCAGCTGAATTATCACCGTTAAGCGTACTTTCACTAAAGAAGGACGT | 49 |
| 22[419] | 9[412] | 22[419]9[412] | TGCGTAGCAAAAGGGTTGAGGCAGGTCATGAAACC | 35 |
| 7[700] | 23[713] | 7[700]23[713] | CCTGAGTAGATAATGTCACGACGTTGTA | 28 |
| 22[643] | 24[651] | 22[643]24[651] | ATCCTTTTAAACCAAGGAAAGGGGGATGACAACTCCAATATCCGGTTTG | 49 |
| 7[574] | 23[587] | 7[574]23[587] | TCAGAGCGAAACCATACGAGCATGTAGA | 28 |
| 20[97] | 24[84] | 20[97]24[84] | GAACAAAGTTACCATTTTTAAGCTTAGAGCCAGAACCTCGTT | 42 |
| 20[209] | 5[216] | 20[209] | CAGAGAGATCAATAGAAAATTTGCCTATTGTAGCAACTCATCAAACACC | 49 |
| 20[230] | 5[237] | 20[230] | AGGGTAATTTACCAGCGCCAAGTATAAACTCATAGGGCAAAAATTGGGC | 49 |
| 20[251] | 5[258] | 20[251] | GAACACCAGGGCGACATTCAAGTGCCTTTAAAGTTGCACCAATTTCAAC | 49 |
| 20[272] | 5[279] | 20[272] | CATTAGAAGGGAGGGAAGGTATAATAAGGACGTTAATACGTAATTACCT | 49 |
| 24[230] | 4[217] | 24[230] | AAGAAAGGTAGTAAGAATACACTAAAAC | 28 |
| 20[223] | 24[210] | refverb20[223] | TTGAGCGCTAATATAAATTTATTCATTTAGAACTCAGCGGGC | 42 |
| 20[244] | 24[231] | refverb20[244] | CTGAACAAAGTCAGCGACCGTAAATTAAGCTGGTAGGAAGGG | 42 |
| 20[265] | 24[252] | refverb20[265] | CGGGAGAATTAACTGTTAAATGAAACAAATTACCGGCCGGCG | 42 |
| 24[209] | 4[196] | refverb24[209] | GCTAGGGTGACGAGTTTGACCCCCAGCG | 28 |
| 24[251] | 4[238] | refverb24[251] | AACGTGGGGTTTAACCTAAAACGAAAGA | 28 |
| 5[217] | 25[230] | refverb5[217] | AGAACGACGAAAGGAAACTATCGGCCTT | 28 |
| 5[238] | 25[251] | refverb5[238] | TTGAGATCGAGAAAATATCCAGAACAAT | 28 |
| 5[259] | 25[272] | refverb5[259] | TTTAATCGGGGAAACCAGCCATTGCAAC | 28 |
| 5[280] | 25[293] | refverb5[280] | TATGCGAGCCCCCGACGCTCATGGAAAT | 28 |
| 5[217] | 25[230] | verb5[217] | TGATGTCTGCAGTCGCTGTTTTAGAACGACGAAAGGAAACTATCGGCCTT | 50 |
| 5[238] | 25[251] | verb5[238] | TTGGGAACTCGTGTCTTTTTTTTTGAGATCGAGAAAATATCCAGAACAAT | 50 |
| 5[259] | 25[272] | verb5[259] | CGCCGACGTGATAGAATGTTTTTTTAATCGGGGAAACCAGCCATTGCAAC | 50 |
| 5[280] | 25[293] | verb5[280] | GCAATCCAGTGTTGGTCATTTTTATGCGAGCCCCCGACGCTCATGGAAAT | 50 |
| 20[223] | 24[210] | verb20[223] | TTGAGCGCTAATATAAATTTATTCATTTAGAACTCAGCGGGCTTTTAATTCGTCTCCGATCAGC | 64 |
| 20[244] | 24[231] | verb20[244] | CTGAACAAAGTCAGCGACCGTAAATTAAGCTGGTAGGAAGGGTTTTATAGTACAGCTTCAAGCA | 64 |
| 20[265] | 24[252] | verb20[265] | CGGGAGAATTAACTGTTAAATGAAACAAATTACCGGCCGGCGTTTTCCACACAGTTGCATCGTA | 64 |
| 24[209] | 4[196] | verb24[209] | GAACTTGGCCTCTAATTCTTTTGCTAGGGTGACGAGTTTGACCCCCAGCG | 50 |
| 24[251] | 4[238] | verb24[251] | AGCCGTCGTGGCGAGCCCTTTTAACGTGGGGTTTAACCTAAAACGAAAGA | 50 |
| 24[60] | 4[763] | poly24[60] | GGAGGCCCCTGGGGTGTTCGAGCACCAGACCGGAAGC | 37 |
| 20[67] | 9[777] | poly20[67] | ACGCAATTCAGGAAGATCGCACTGACGACAG | 31 |
| 25[772] | 24[61] | poly25[772] | TGTAAAGGATTAAAGGGATTTTAAGCTAAACA | 32 |
| 9[778] | 6[777] | poly9[778] | TATCGGCCAATAACGGAGGTGTATCACCGTTCTA | 34 |
| 7[777] | 4[777] | poly7[777] | AACCGTACTCAGGAGGTACCGAACTGACCAGCGA | 34 |
| 23[62] | 22[773] | poly23[62] | ATCATAGGAGAGTCAATAGTGAATCTCGAA | 30 |
| 5[777] | 25[83] | poly5[777] | TTCAAAACTTTGAAAGAGGACAGAGAGCGGGGACAGGAACGGTAC | 45 |
| 7[763] | 23[61] | poly7[763] | TCAATATGTAATCAATCCCCGGGTACCGAGTTATCAAA | 38 |

Ring 1

| Start | End | Oligoname | Sequence | Length |
| --- | --- | --- | --- | --- |
| 31[105] | 8[84] | 31[105] | GCAAGACTTAGTTAATTTCATAATCATATAATGAGTGTAGCAATACTTC | 49 |
| 18[125] | 28[112] | 18[125] | GGGTAACGCCAGGGCTAAGTGAGTAATAATTTAGGGTATAAA | 42 |
| 73[112] | 66[112] | 73[112] | GATGAACACAAGAAAGCTGCTACACCAGATTTCAATAAGAAC | 42 |
| 55[70] | 50[77] | 55[70] | AATCACCGGGGTGTCTGGAAGATCAAGTCAGCACCTTATCACCGTCACC | 49 |
| 20[62] | 25[60] | 20[62] | ATATCAAACCCTCATGCCACGCCCGAACCATTTTGCGGAA | 40 |
| 72[76] | 43[62] | 72[76] | CAATGACAACAACCGAATTTCCGCATTAGACGGGATTTAACG | 42 |
| 52[62] | 54[49] | 52[62] | AGCGCGTGTACTCAGTCGAGAGGGTTGATTAGCGGCCTCCCT | 42 |
| 30[97] | 8[77] | 30[97] | CTTCTGACCTAAATTTAATGGTTAAATATTTGATT | 35 |
| 5[91] | 2[85] | 5[91] | GGGAGAGCGCCTGGCCGCTGCGTCACCTGAGAGAGATGGTGGTTCCGA | 48 |
| 57[49] | 52[63] | 57[49] | GAGCCACCACCCTCAGAGCCGCACCACCTAATCAAAGACTGT | 42 |
| 10[55] | 25[48] | 10[55] | GGAAAAACGCTCATATGATGGTTATCATACATTAT | 35 |
| 13[56] | 16[63] | 13[56] | CGAATATAGGGGCCGCAACTCTTTTTGAATTTTACATCTAGGCTATTAG | 49 |
| 40[69] | 71[76] | 40[69] | AAAATAATCAAAAATGAAAATAGGGAAGTTAAACATTTTCACAGTGAGA | 49 |
| 38[83] | 40[70] | 38[83] | CGAGTAACAACCGAGGTTTTGTGAATCTTACCAACCAGTTAC | 42 |
| 32[69] | 6[77] | 32[69] | GCAGAGGAAGAAAACAAGAGTCAATAGTAGTTACAATTCGCCACAGGAG | 49 |
| 25[61] | 21[62] | 25[61] | CAAAGAAACTATTAAAACTTTACGGAGCACTAACAACTTGAAAG | 44 |
| 10[123] | 6[112] | 10[123] | GTACCGAGCTCGCAATTCCAAGCCTGTTGCGTTTGTCGTG | 40 |
| 32[76] | 1[69] | 32[76] | AAATCGCTGGCAAGGCCCCCGATTTAGAATCGGAA | 35 |
| 59[91] | 55[111] | 59[91] | CCTCATTAAAAAAAGATTAAGACCAGACTTGATAAGCTCAACACAGTTG | 49 |
| 46[96] | 51[111] | 46[96] | AAAGTTGACCCTGAAATCGGCGGAAATTATTCTTAACATCC | 41 |
| 5[112] | 6[105] | 5[112] | GGGCGCCCAACAGCTGATTGCCCTTCACGCGGTTTGCGTATTCCAGCTG | 49 |
| 31[56] | 29[69] | 31[56] | CATCGGGTTTTAGACCGAGTAAAAGAGTACATCACTGATAAA | 42 |
| 67[105] | 51[90] | 67[105] | TGGGAAGTGGCTCATTATACCAGCCCTCAGAACCGATATTGA | 42 |
| 44[97] | 45[104] | 44[97] | ATATTCAGTAAGCAGATAGAATGCCTGATCACCATCAATATGGGTCATT | 49 |
| 69[39] | 47[41] | 69[39] | TAACACTGAGGTTAGTACAAACGTAAAAGAACTGGCATGAGCAAG | 45 |
| 65[70] | 63[83] | 65[70] | CCAGGGAATACCACACCAAAATAGAAACATTCTGCGAGAGGC | 42 |
| 75[98] | 41[118] | 75[98] | GGAGATTAAAGAATAGTTTCCATTAAACGCCAGCTTTAAATCAGCTCAT | 49 |
| 27[76] | 24[70] | 27[76] | TGGATTAATCGCCATAAAGGTAACATGTTTTACGAGCATGTGACAACTCG | 50 |
| 31[63] | 3[55] | 31[63] | AGAAACATACCGACCGGTAGATTTTCAGCCTTTTATTCATTTAAGGAGC | 49 |
| 36[83] | 22[70] | 36[83] | GCCCAATAGCAAGCGGAAGGTATCTTTA | 28 |
| 18[80] | 21[69] | 18[80] | CCAGTGAGAAGATAAAACCCAGCAGACCTCAAGAATTGA | 39 |
| 9[77] | 10[105] | 9[77] | GTAATATCCTCCTGTGTGAAATTGTTATCCGCTCAAATTCGT | 42 |
| 53[70] | 64[70] | 53[70] | GAAACCATCGATAGTTGCCTTGCCACCCGAGATTTAGCGGAT | 42 |
| 25[70] | 26[56] | 25[70] | GACGACAATAAACAAAGTAATTCTGTCCAGACCACCAGAAGG | 42 |
| 40[104] | 43[104] | 40[104] | GTTAAAATTCGCCCAGAGCCTTTTACAGAGAGAAAAGCCCCA | 42 |
| 45[56] | 46[70] | 45[56] | TGAGCAGGGTAGCTATTTTTGTAAATTACCGAAGCGAGGAAA | 42 |
| 24[97] | 17[104] | 24[97] | TCCTAATCAGCTAATGCAGAAATAAAGTGGGTGGA | 35 |
| 27[105] | 22[98] | 27[105] | ACATGTAAGAGAATCGCGCCTAAAATAATTCCTTAGCACTCATCGAGAA | 49 |
| 2[84] | 6[84] | 2[84] | AATCGGGATGTAGCGGCGTAACGCGGGAGCTGCGCGCCAACAA | 43 |
| 31[84] | 6[91] | 31[84] | TGATTGCTTGCAAATCCAATCCATTAATGAATCGG | 35 |
| 9[45] | 27[75] | 9[45] | TTTTTTAAGAACTCAAACTATCGGCCAGCCATAATCCTGATTGTT | 45 |
| 65[63] | 51[69] | 65[63] | CTCAGTAAAGTGCCGGAGGTTACCACCCCGATTGA | 35 |
| 21[49] | 24[56] | 21[49] | TCAACAGTAATAGATTTAGAAGTATTAGTCCTTTG | 35 |
| 35[77] | 35[76] | 35[77] | TGAATAAAAGACGCTGAGAAAATTAATTTCAATATATGTGAG | 42 |
| 40[118] | 45[111] | 40[118] | AACGTTATCGCCTGGCGCAGACGGTCAAGAGGACATGTACCCGCCTGAG | 49 |
| 28[69] | 11[76] | 28[69] | AATGGAAGGGTTAGTCAATATTGCAACAGATTATTTACATCTCCTGGTT | 49 |
| 75[91] | 74[77] | 75[91] | GTACAACGGAACCGAATAGTTGCGCCGAAAGCGCGATCTTTG | 42 |
| 15[77] | 12[56] | 15[77] | GCTCGCCCCTGCCATCTGTAATTGAATCAACTCTGACTGGCAGATTCAC | 49 |
| 36[69] | 39[83] | 36[69] | AAATCAGTTTGACTTGCGGCGTCGGATTCTC | 31 |
| 60[83] | 62[63] | 60[83] | TCCCCCTCAACGGGGTCAGTGACCTATTATGAAAG | 35 |
| 61[112] | 58[92] | 61[112] | AATCAAAAAGCAAAGCGGATTGCATCAGCCAGAA | 34 |
| 39[84] | 75[90] | 39[84] | CGTGGGAACAAACGACATTAAATGAGGAACACTAAAACACTCAAACAAA | 49 |
| 10[76] | 6[63] | 10[76] | TTACCGCCTTGCTGAGTAATACTGTCCAGCCGATTAAAGGGA | 42 |
| 71[49] | 52[49] | 71[49] | AACTTTCTCCAGACTTTCGTCCAGGGATTATCACCTTTCATC | 42 |
| 54[104] | 56[91] | 54[104] | ATGTTTTGATACATGTTTACCAGACGACAAAGAAGAAACTCC | 42 |
| 48[90] | 50[70] | 48[90] | TTGTACCAAAAGAAACGCAAAGGGAGGGGACTTGA | 35 |
| 40[62] | 36[49] | 40[62] | ACAGCCATTTATCCAAGCCTTACCTCCCTTTATATAGAAGGCTTA | 45 |
| 42[104] | 45[97] | 42[104] | CGGTTGATAATCAGAATAACATACAAAGGCTATCA | 35 |
| 77[63] | 75[76] | 77[63] | AGAGGCTTTGAGGAACCCCCAGCGTCACGGATCGATTATACC | 42 |
| 41[98] | 36[84] | 41[98] | ATTTTTGTTCATCAGCGGATTTTTCAGTATCGGAATCATTACCGC | 45 |
| 55[49] | 62[56] | 55[49] | TTGCCATCTTTTCAGGAACCGGGTTTTGTATTAAG | 35 |
| 27[49] | 8[46] | 27[49] | CAATTCAAACCTACTAAAGAAATTGCTGTTGCCTGAGTAGTTTTT | 45 |
| 21[105] | 77[118] | 21[105] | GATCGCAACGACGATTTGACCGTAATGGGATTCCTGTAGGGTAAA | 45 |
| 17[63] | 16[77] | 17[63] | CGAACCACCAGCCCAAGCTTTGCTTCTA | 28 |
| 67[84] | 54[91] | 67[84] | TCAGAACCGCCATCAGGACGTAGGTAGAAAGATTCTAATGCAAAATATG | 49 |
| 7[77] | 7[111] | 7[77] | TCACGCAAATTAACCGTTGAGCTAACTCACATTAA | 35 |
| 22[97] | 19[104] | 22[97] | CAAGCAAATAATCGGCTGTCTTATCCCAGGGCCTC | 35 |
| 70[83] | 72[98] | 70[83] | AATAATTGCTTGATACCGACTGACCAACTTTGAAATCATAAG | 42 |
| 20[77] | 19[97] | 20[77] | CTTGCTGACAAATGAAAAATCTAAGGCGATCGGTGC | 36 |
| 68[76] | 47[62] | 68[76] | GATCTAAAGTTTTGTAAAGGTCGCAATAATAACGGCAATAGC | 42 |
| 19[56] | 26[49] | 19[56] | CTGAGAGAGAGGTGAGGCGGTTTAAAAAAGCGGAA | 35 |
| 70[62] | 77[62] | 70[62] | ATCTCCATCGAGGTATCGCCCTTTTGCGCCTCAGCCGGCTAC | 42 |
| 16[104] | 11[83] | 16[104] | ATGTCCCGCCAAAAACAGGGCGTCATAGCTGTTAGAACAATAGGTGTAA | 49 |
| 44[90] | 41[83] | 44[90] | ACCGTTCTAGCTGAAGAGATCTAAAAACAGCAGCCAATTTGCGCTAACG | 49 |
| 77[77] | 41[97] | 77[77] | CTAAAGACTTTTTCATGTGAGAGCGTCTTTATTAA | 35 |
| 46[55] | 53[62] | 46[55] | AATACCCAGAAAATAGTTTATGGGCGACAGAGCCAAAGGCCGGAAACGT | 49 |
| 58[91] | 54[70] | 58[91] | TGGAAAGCAATAAATAACAGGTGAGTACCCAACTAAAGTACAACCAGAGC | 50 |
| 29[84] | 31[76] | 29[84] | AGAATAATTAATTGAGATACTTCTGAATTAAGGCGTTTGAAAATAACGG | 49 |
| 8[111] | 10[98] | 8[111] | GGGTGCCATTACTAGCCAACGCTCAACAAACGCCAAATCATG | 42 |
| 11[98] | 20[105] | 11[98] | TTAAGCTACGTGGTTATGACATGTTCTTTTTTCCCTTCGCTAATTCAGG | 49 |
| 18[104] | 29[97] | 18[104] | AGTCACGACGTTGTGAAGCCAACCGACAATTTAACGTAGGGCACACCGG | 49 |
| 17[56] | 14[49] | 17[56] | TACCGAATCTTTAAGACAATAGTCGGTGGGCCCTT | 35 |
| 49[70] | 47[83] | 49[70] | GGCAACATATAAAAACATTATACCAGAAGGAAACCCCTTTTT | 42 |
| 21[91] | 20[78] | 21[91] | GTAGGCCTCAGGAACTGCGCAACTGTTGGGAAGAGCATCAC | 41 |
| 50[111] | 53[111] | 50[111] | GTAGTAGCAATTAAAGGTGAAGTAATCAGTAACCTGTTTAGC | 42 |
| 45[112] | 51[118] | 45[112] | AGTCTGGAGTCAAAGTAATGTCATATATGAGAAGCAAAGCCTAATAAAT | 49 |
| 23[70] | 21[90] | 23[70] | AAACAATTCAGAAACCAATCAGCCGTTTTTAAAATTATCTATTTTCATC | 49 |
| 4[62] | 3[69] | 4[62] | CGCTTAAGCTTTCCTCGTTAGAATCAGACACCACACCCGCCGAGGGCGC | 49 |
| 34[104] | 32[84] | 34[104] | AACATAGCATAGGTGTAAATGCTGATTGAATACCA | 35 |
| 61[84] | 64[84] | 61[84] | AACAGTTCATTGAATTTTGCAGATAAAAATTCAACATCAGTT | 42 |
| 33[84] | 0[84] | 33[84] | GAATTTATCAAAATCGATAGCTAAATCACAACGTCAAAGGGC | 42 |
| 0[62] | 3[62] | 0[62] | ATGGCCCGCACTAAGCTTGACGGGCGCT | 28 |
| 61[49] | 58[35] | 61[49] | CAGGAGTGCGTCATACATGGCTTTTTTT | 28 |
| 61[70] | 63[62] | 61[70] | TTTTAAACTGAATTTACCGTTTGGCCTTGAGCCGCCGCCAGCTTTCGGA | 49 |
| 56[118] | 61[125] | 56[118] | AAAGCGAAGGAAGCCCGAAAGTAGTCAGAATCAGG | 35 |
| 0[125] | 1[140] | 0[125] | AGAGTCCTTGAGTGTTGTTCCAGTTTTTT | 29 |
| 1[98] | 32[105] | 1[98] | AAAGAATCTGTTTGTTGCAGCAAGCGGTACTATAT | 35 |
| 1[70] | 35[97] | 1[70] | CCCTAAAGGCAAAATCCCTTATTAGATTCCTTGCTTCTGTAA | 42 |
| 35[113] | 0[112] | 35[113] | TAATTTTCTTTTTTTTTTCCTTAGAATGATAGGGACTATTA | 41 |
| 6[125] | 8[126] | pass6[125] | GGAAACCGCGCTCACTGCCCGCTTTTTTTTTTTTTTTTTGGAAGCATA | 48 |
| 77[49] | 74[34] | pass77[49] | GTAGCAAAGCGAAAGACAGCATCTTTTTT | 29 |
| 74[118] | 42[112] | pass74[118] | AAACGAAAGAGGCATGTATCAATATTTTAAAACAGGAAGATTAATCATA | 49 |
| 57[112] | 63[136] | pass57[112] | GAGGTCATTTTTGCGAGCTTCTAAAATGTTTAGTTTTTT | 39 |
| 31[30] | 6[24] | pass31[30] | TTTATATACAGTCAGAATCCTGAGTTTTTT | 30 |
| 14[105] | 15[105] | pass14[105] | TTTTTTATACCGACAGTGCGGCCTGGAGTGACTCTATGTTTTTT | 44 |
| 29[112] | 3[129] | pass29[112] | GAAAAAGATATATTAAAGAACTTATATACCACGCTGGTTTTTTTTT | 46 |
| 39[31] | 38[49] | pass39[31] | TTTTTTACGCGAGGCGTTTTAGCGAAAATCAA | 32 |
| 44[48] | 44[17] | pass44[48] | GAGATAACCCACAAGAATTGAGTTAATTTTTT | 32 |
| 45[30] | 47[55] | pass45[30] | TTTTTTTAACAAAGTCAGAGGGTAATTATCAGAGAAATAG | 40 |
| 61[32] | 60[32] | pass61[32] | TTTTTTTTTTGATGATAGCCCGTATAAACTTTTT | 34 |
| 55[28] | 54[27] | pass55[28] | TTTTTGCCCCCTTAGCCACCCTCTTTTTT | 29 |
| 72[149] | 42[126] | pass72[149] | TTTTTGCTCCATGTTACTTAGCCGAGACCAGAAAACTA | 38 |
| 2[41] | 4[25] | pass2[41] | AACGTGGCGAGATTTTTTTTAAGGAAGGGAAGGCGCGTACTATGTTTTT | 49 |
| 63[32] | 62[30] | pass63[32] | TTTTTAGTTAATGCCCCGACTCCTCAAGATTTTTTT | 36 |
| 16[147] | 20[126] | pass16[147] | TTTTTTTAAGTGTCCTTAGTGCATTCATGTTAAGTTGAAAGGGCCAGGCA | 50 |
| 36[125] | 23[133] | pass36[125] | GCCAGTTTGAGGGGCTCCAGCAAGTACCTCATTCCAAGAACGGGTTTTTT | 50 |
| 32[136] | 30[119] | pass32[136] | TTTTTTGCTTAGGTTGGGGCGAGAAAATTTTTTTTTTCTTTTTCAA | 46 |
| 67[112] | 65[133] | pass67[112] | AAAAATCCATTTCGATTCCCAATTCTGCAATATAATTACGAGGCATATTT | 50 |
| 4[141] | 4[119] | pass4[141] | TTTTTTTTCACCAGTGAGACGGG | 23 |
| 72[41] | 75[48] | pass72[41] | TATTCGGTCGCTTTTTTTTTGAGGCTTGCAGGGAGTTAA | 39 |
| 2[128] | 35[112] | pass2[128] | TTTTTGCCCCAGCAGGCGAAAATCAGCCCGACCTTGAACTATTAAT | 46 |
| 15[31] | 11[55] | pass15[31] | TTTTTTCGTAAGAATACGTGGCACATGCGCGATGAAATG | 39 |
| 29[126] | 27[131] | pass29[126] | AGTATCATATGCTTTTTTGTTATACAAATTCTTACCACAGAGGCATTTTT | 50 |
| 28[48] | 10[25] | pass28[48] | CATATCAAAATTATTTTTTTTTTTGATTATCAGGGAAATACCTACTTTTT | 50 |
| 12[83] | 13[97] | pass12[83] | TTCTCCGGGCTGACGCATTTCACTTTTT | 28 |
| 40[137] | 39[125] | pass40[137] | TTTTTTTATTTAAATTGTATTTTTAACTGGCCTAGGTCAC | 40 |
| 22[132] | 19[118] | pass22[132] | TTTTTTATTAAACCCAGCTTTAAGCGCCATTCGCCTTACGCC | 42 |
| 24[48] | 22[28] | pass24[48] | GTTATTATTGAGGATTAGAGCCGTCAATAGTTTTT | 35 |
| 17[32] | 18[32] | pass17[32] | TTTTTAAAACATCGCCACAGTATTAACACTTTTT | 34 |
| 29[119] | 10[124] | pass29[119] | CCTGTTTAAGTGTAACACAACATACGAGCCTTTTTTTTAGGATCCCCGG | 49 |
| 2[55] | 34[31] | pass2[55] | GGGGAAAGCCGGCGCGAGGTGCATTTGAATTACTTTTTT | 39 |
| 60[136] | 60[112] | pass60[136] | TTTTTTACTGGATAGCGTCCAATAC | 25 |
| 5[24] | 31[48] | pass5[24] | TTTTTTGTTGCTTTGACGAGCACGTGGTACGCAACAGTA | 39 |
| 36[145] | 20[133] | pass36[145] | TTTTTCGTAACCGCGCTTCTGGTTTTTTTTTTTGCCGGAAA | 41 |
| 71[23] | 47[48] | pass71[23] | TTTTTTTGTATGGGATTTTGCTAAACGGCTCCAAAACAAT | 40 |
| 42[146] | 73[150] | pass42[146] | TTTTTGGTAATCGTGCGCATAGGCTGTTTTTT | 32 |
| 56[144] | 57[144] | pass56[144] | TTTTTTTGCGTTTTAATTCGGATGGCTTAGATTTTTTT | 38 |
| 7[24] | 6[49] | pass7[24] | TTTTTTAAGTGTTTTTATAATCAGTGAGGCCACAGGAAC | 39 |
| 57[27] | 54[42] | pass57[27] | TTTTTTAGAACCGCCACCCTCACAGAGCC | 29 |
| 72[55] | 42[31] | pass72[55] | ACGCATAACCGATATTATCAGCTGAACACCCTGTTTTTT | 39 |
| 12[97] | 18[81] | pass12[97] | TTTTTATAAATCATTGAGTAATAACCCCCTCAGGAAAAACGACGG | 45 |
| 24[133] | 19[125] | pass24[133] | TTTTTTTAAGTCCTGAGCTGGC | 22 |
| 70[41] | 73[41] | pass70[41] | AAAGGAGCCTTTTTTTTTTTTTTTTTAATTGTATCGGT | 38 |
| 50[118] | 67[136] | pass50[118] | ACTAATATATATTTTCATTTGTTAGATATACGTTAATAAATTTTTT | 46 |
| 30[48] | 29[48] | pass30[48] | GTTTAACGTCAGATGATTTTTTTTTTTTTTTTGCACGTAAAACAGAAA | 48 |
| 23[29] | 26[29] | pass23[29] | TTTTATAATACATATTTTAAAATTTTTTGTTTGAGTACATATTCCTTTTT | 50 |
| 19[32] | 20[32] | pass19[32] | TTTTTCGCCTGCAACAGATCAATATCTGGTTTTT | 34 |
| 48[125] | 71[140] | pass48[125] | AAGCAATCTTTATTACGAGAACATTCAGTGAATAAGGTTTTTT | 43 |
| 51[31] | 66[24] | pass51[31] | CCAGCGCCAAACCAATAGGAACCTTTTTT | 29 |
| 5[119] | 6[126] | pass5[119] | AGGGTGGTTTTTCTTTTTTTTTTTTTTTTTTTTTTCCAGTCG | 42 |
| 19[133] | 17[146] | pass19[133] | GGATGTGCTTTTTTTTTTTGCAAGGCGACGCACGACTTTTTT | 42 |
| 21[126] | 38[133] | pass21[126] | CCGGCACTGCATCTTTTGTTGGTGTAGATGGGCTTTTTTTTCAAAAATAA | 50 |
| 55[42] | 52[25] | pass55[42] | TTAGCGTGGCATTTTCGGTCATATTTTTTTT | 31 |
| 16[125] | 11[140] | pass16[125] | TGAATTGTCAACCTGCTTGTTACCTCGATAAAGACGGTTTTTT | 43 |
| 5[49] | 32[30] | pass5[49] | ATAACGTTGCGCCGCTACAGGAAAGCGACAATTACCTGAGTTTTTTT | 47 |
| 49[19] | 51[30] | pass49[19] | TTTTTTATTACGCAGTTAGAAAATTCATTTTTTTTTTTTTTTATGGTTTA | 50 |
| 40[48] | 75[55] | pass40[48] | TATCCCAATCCATTTTTTTTGCACCCAGCTACAATTATTATTAGGCCGC | 49 |
| 21[31] | 36[33] | pass21[31] | TTTTTTTCAGTTGGCAAATCCGGTATTCTATTTT | 34 |
| 0[55] | 1[41] | pass0[55] | ACTACGTGAACCATCACCCAAATCAATTTTTTTTTTGTTTTTTGGGGT | 48 |
| 66[139] | 48[126] | pass66[139] | TTTTTTTACCTTATGCAAGGCAAAGATTTTTTTTTTATTAGCAAAATT | 48 |
| 24[118] | 17[125] | pass24[118] | AACAAGAGTTTATCAACAATAGATTTTTTTTTTTTTCGAGCCGTTGTGA | 49 |
| 43[31] | 70[49] | pass43[31] | TTTTTTAATAAGAAACGATTTTTTGGAATTAACTTGCTTAAAAAAA | 46 |
| 38[132] | 41[148] | pass38[132] | TTCGCGTCCAATAGGAACGCCATTTTTTTT | 30 |
| 62[55] | 56[39] | pass62[55] | AGGCTGACTGCCTAATTGACAGGAGGTTTTT | 31 |
| 77[35] | 38[31] | pass77[35] | TTTTTGGAACGAGGGATTAGTTGCTATTTTTT | 32 |
| 62[135] | 64[105] | pass62[135] | TTTTTGTAAGAGCAACACTATCATAACCCTCAACGCCATTATTAC | 45 |
| 74[140] | 75[151] | pass74[140] | TTTTTTCGAAGGCACGTGTCGAAATCCGCGACCTTTTTTTT | 41 |
| 45[126] | 44[119] | pass45[126] | AAGAGAATCGATGAACTTTTTTTTTTTTTTTGGTGAGAAAGGCCGGAGAC | 50 |
| 67[30] | 51[48] | pass67[30] | TTTTTTTCCGGAATAGGTGAGCAAGCGACAAAA | 33 |
| 46[143] | 70[119] | pass46[143] | TTTTTTTAAAAATTTTTAGAACCCTGTAGGTAAATCTTG | 39 |
| 54[143] | 55[145] | pass54[143] | TTTTTTGCTTAATTGCTGGAACGAGTAGATTTTTTTTT | 38 |
| 47[16] | 46[17] | pass47[16] | TTTTTTTGCCCAATAATAAGATTAAGACTCCTTTTTT | 37 |
| 33[30] | 1[55] | pass33[30] | TTTTTTTCAAAAGAAGATGATGAAACACAATTTCCGTAAA | 40 |
| 33[105] | 33[136] | pass33[105] | CTGAGAGACTACCTTTTTAACCTCCGTTTTTT | 32 |
| 73[119] | 77[139] | pass73[119] | GGTGTACGAACGAGATAAATTCAACCTAATACGTAATGCCACTATTTTT | 49 |
| 65[30] | 64[31] | pass65[30] | TTTTTTTGAAGGATTAGGATATAAGTATAGCTTTTTT | 37 |
| 45[119] | 43[137] | pass45[119] | AGCAAACGCATGTCGTATAAGCAAATTTTTTTT | 33 |
| 48[48] | 69[38] | pass48[48] | TTTGTCACAATCAAATGTTAGAATGAATTTTCTTTTTTTTTCATGTACCG | 50 |
| 64[136] | 54[112] | pass64[136] | TTTTTTACGAACTAACGGAACAACAAAAGGAATGCTGTA | 39 |
| 70[149] | 47[143] | pass70[149] | TTTTTGCTGACCTTCATCAAGAGTAAGATTCAAAAGTTTTTT | 42 |
| 69[84] | 71[83] | ref_verb69[84] | TTCCACAATAGTTAGCGTAACATAGAAA | 28 |
| 69[63] | 52[70] | ref_verb69[63] | CAAACTACAACGCCCAGAGCCTAGTACCTAGCGTC | 35 |
| 69[105] | 46[97] | ref_verb69[105] | TGGTTTAAACGAGTTTTGCGGTTTAAATGCCCGAAC | 36 |
| 71[63] | 44[56] | ref_verb71[63] | TCAGCGGGTTGAAATATCTTAATGCCGGAGGCTAA | 35 |
| 71[84] | 71[104] | ref_verb71[84] | GGAACAACTAAACAAATCAAC | 21 |
| 48[69] | 71[62] | ref_verb48[69] | GACACCACGGAATAACATACATCGTCTTAACAGTT | 35 |
| 71[105] | 47[90] | ref_verb71[105] | GTAACAACCGGATATTCATTACCGGAATTGCGAATAAGAAAA | 42 |
| 48[111] | 69[104] | ref_verb48[111] | CAGAGCATAAAGCTTAATACTAGTAAATTGCCCTCGACAGGGCTTGAGA | 49 |
| 50[143] | 69[125] | ref_verb50[143] | TTTTTTAAAAGGTGGCATCAATTCTCATACAGGCGATTTCTTTAAT | 46 |
| 51[77] | 69[83] | ref_verb51[77] | AAGGTAACCACCCTTGTAGCA | 21 |
| 53[63] | 69[62] | ref_verb53[63] | CACCAATGCCATTTGGGAATTATTCAACTCATTTTACCAGTATCAGCGACTGCAGACATCAGATCAGAG | 69 |
| 48[111] | 69[104] | ring_verb48[111] | CAGAGCATAAAGCTTAATACTAGTAAATTGCCCTCGACAGGGCTTGAGATCATTCTATCACGTCGGCGACCACTAG | 76 |
| 50[143] | 69[125] | ring_verb50[143] | TTTTTTAAAAGGTGGCATCAATTCTCATACAGGCGATTTCTTTAATTTGACCAACACTGGATTGCTTTTCACT | 73 |
| 51[77] | 69[83] | ring_verb51[77] | AAGGTAACCACCCTTGTAGCATAAAGACACGAGTTCCCAAAACCAGGC | 48 |
| 53[63] | 69[62] | ring_verb_gr53[63] | CACCAATGCCATTTGGGAATTATTCAACTCATTTTACCAGTATCAGCGACTGCAGACATCAGATCAGAG | 69 |
| 71[63] | 44[56] | ring_verb71[63] | TATAGTGCGCTGATCGGAGACGAATTTTCAGCGGGTTGAAATATCTTAATGCCGGAGGCTAA | 62 |
| 71[84] | 71[104] | ring_verb71[84] | CAGGCTATTGCTTGAAGCTGTACTATTGGAACAACTAAACAAATCAACTGGGCTCGCCACGACGGCTAATCCTGG | 75 |
| 48[69] | 71[62] | ring_verb48[69] | GACACCACGGAATAACATACATCGTCTTAACAGTTTGAATTAGAGGCCAAGTTCGATCCCTA | 62 |
| 71[105] | 47[90] | ring_verb71[105] | CACCGACGTACGATGCAACTGTGTGGTGTAACAACCGGATATTCATTACCGGAATTGCGAATAAGAAAA | 69 |
| 69[84] | 71[83] | fluoro69[84] | Cyanine5-TTCCACAATAGTTAGCGTAACATAGAAA | 37 |
| 69[63] | 52[70] | fluoro69[63] | Cyanine5-CAAACTACAACGCCCAGAGCCTAGTACCTAGCGTC | 44 |
| 69[105] | 46[97] | fluoro69[105] | Cyanine5-TGGTTTAAACGAGTTTTGCGGTTTAAATGCCCGAAC | 45 |
| 69[126] | 49[143] | fluoro69[126] | Cyanine5-CATTGTGAATTTTTTTTTTTTCTTGCCCTGTCAACGCAAGGATTTTTT | 57 |
| 13[49] | 16[31] | paint13[49] | TAGAACACAGTCACACGACCAAATCGTCACTGATAGCCCTTTTTTTTTATACATCTA | 28 |
| 11[23] | 12[24] | paint11[23] | TTTTTTTATTTTGACGCTCGTAATAAAAGGGTTTTTTTTATACATCTA | 35 |
| 13[24] | 14[30] | paint13[24] | TTTTTTACATTCTGGCCAACAGAGACTGACCTGAAAGTTTTTTTTTATACATCTA | 36 |
| 13[49] | 16[31] | Thiol13[49] | TAGAACACAGTCACACGACCAAATCGTCACTGATAGCCCTTTTTTTTTGAGAGAGAGAGAGAGAGAGA | 28 |
| 11[23] | 12[24] | Thiol11[23] | TTTTTTTATTTTGACGCTCGTAATAAAAGGGTTTTTTTTGAGAGAGAGAGAGAGAGAGA | 35 |
| 13[24] | 14[30] | Thiol13[24] | TTTTTTACATTCTGGCCAACAGAGACTGACCTGAAAGTTTTTTTTTGAGAGAGAGAGAGAGAGAGA | 36 |
| 57[70] | 58[77] | biotin57[70] | biotin-CCACCAGAATTAGACAGGACCACCACCAGATATTCACAAACAGCAGTCT | 48 |
| 52[111] | 55[104] | biotin52[111] | biotin-CAAATGGTCAATAGCGACAGATTTCATTCCATATA | 57 |
| 58[141] | 59[143] | biotin58[141] | biotin-TTTTTTTTGACTATTAACTTCAAATATCTTTTTT | 48 |
| 53[28] | 50[27] | biotin53[28] | biotin-TTTTTCACCATTACCATTAGCGCAAAATCACCAGTAGTTTTTT | 55 |
| 52[144] | 53[144] | biotin52[144] | biotin-TTTTTTTTTAGTTTGACCAGGGCGCGAGCTGTTTTTTT | 56 |
| 59[39] | 61[62] | biotin59[39] | biotin-TTTTTTTGAGGCAGGTCAGACGATCCAGTAAGTACTGG | 42 |
| 61[77] | 61[69] | verschluss61[77] | TGCTTTAGAAAAACCGTCTATCAGGGCGTAATAAG | 34 |
| 60[97] | 0[98] | verschluss60[97] | AAATATTCAGAAAACGAGAATTGGACTC | 43 |
| 0[140] | 61[141] | verschluss0[140] | TTTTTTTTTGGAACATCTTTACCCTTTTTTT | 38 |
| 35[33] | 60[49] | verschluss35[33] | TTTTCTTTTTTAATGGAAACAGTTAACAGT | 45 |
| 57[91 | 35[104] | verschluss57[91] | TTTAATTGCTCCTTCGGAAGCTTTTGCCTCGTCATATCGTCG | 35 |
| 0[111] | 63[118] | verschluss0[111] | AAGAACGGACCATATGCGGAAAGAGGGGGTAATAG | 28 |
| 60[62] | 32[56] | verschluss60[62] | CCTTGAGACATAAAACATTTAAAACATCCGAATTA | 31 |
|  |  | antireleaseY9 | TGTGTGGATTGTTCG | 15 |
|  |  | antireleaseY8 | CGACCATTCAGCGAC | 15 |
|  |  | antireleaseY7 | GTACTATATCGTGAC | 15 |
|  |  | antireleaseY6 | CGATCTTTGAATTAG | 15 |
|  |  | antireleaseY5 | GTTATTGCAAAGACA | 15 |
|  |  | antireleaseY4 | ACGAATTACCGGAAC | 15 |
|  |  | antireleaseY3 | GCGGAAATTGACCAA | 15 |
|  |  | antireleaseY2 | CGCAAACTCATTCTA | 15 |
|  |  | antireleaseY1 | CGAGCTTTCAGCGAC | 15 |
|  |  | antireleaseX9 | CACCGACGTACGATG | 15 |
|  |  | antireleaseX8 | GACATCAGATCAGAG | 15 |
|  |  | antireleaseX7 | CAGGCTATTGCTTGA | 15 |
|  |  | antireleaseX6 | CAAGTTCGATCCCTA | 15 |
|  |  | antireleaseX5 | TTCCCAAAACCAGGC | 15 |
|  |  | antireleaseX4 | TATAGTGCGCTGATC | 15 |
|  |  | antireleaseX3 | GGATTGCTTTTCACT | 15 |
|  |  | antireleaseX2 | GTCGGCGACCACTAG | 15 |
|  |  | antireleaseX1 | GACATCAGATCAGAG | 15 |
|  |  | release9 | CGAACAATCCACACAGTTGCATCGTACGTCGGTG | 34 |
|  |  | release8 | CTCTGATCTGATGTCTGCAGTCGCTGAATGGTCG | 34 |
|  |  | release7 | GTCACGATATAGTACAGCTTCAAGCAATAGCCTG | 34 |
|  |  | release6 | TAGGGATCGAACTTGGCCTCTAATTCAAAGATCG | 34 |
|  |  | release5 | GCCTGGTTTTGGGAACTCGTGTCTTTGCAATAAC | 34 |
|  |  | release4 | GTTCCGGTAATTCGTCTCCGATCAGCGCACTATA | 34 |
|  |  | release3 | AGTGAAAAGCAATCCAGTGTTGGTCAATTTCCGC | 34 |
|  |  | release2 | CTAGTGGTCGCCGACGTGATAGAATGAGTTTGCG | 34 |
|  |  | release1 | CTCTGATCTGATGTCTGCAGTCGCTGAAAGCTCG | 34 |

Ring 2

| Start | End | Oligoname | Sequence | Length |
| --- | --- | --- | --- | --- |
| 11[112] | 7[118] | 11[112] | CCCTTAGAATCCGGCGAATCAGTATTCACCTTGCTGAACCTCATTAATT | 49 |
| 35[287] | 38[273] | 35[287] | TAATGTGAAAATTAAGCAATAACATCCATGCGAACGAGTAGA | 42 |
| 39[280] | 26[273] | 39[280] | CTAAAGTAACAAAGGAGTAATCTGCTCCATGTTACGAAACAATTCATGA | 49 |
| 12[216] | 2[196] | 12[216] | CCCATCCAAATGAGTCCACTATTCTTTCCAGTGTGTGAAATTGTTAT | 47 |
| 1[112] | 12[119] | 1[112] | GCGCCGCAAGTTTTGTGATAAATAAGTCAAAA | 32 |
| 36[265] | 41[258] | 36[265] | AAAGCTACTACTAATGACCATTAGATACTGTAGCTTAATTGCAAAGATT | 49 |
| 40[251] | 38[231] | 40[251] | TCCTTTTAAATTTTTGTACCAAAAACATGCTGAAAAAATGGTCAATAAC | 49 |
| 0[237] | 3[223] | 0[237] | GTTGTTCCAGTTTTGCCAGCTCGTAATCATGGTCATAGGATAGC | 44 |
| 5[91] | 7[111] | 5[91] | CCAGCAGAAGCAAATATACAGTAACAGATGGCACTTTGCCCGAACGTT | 48 |
| 6[111] | 1[111] | 6[111] | AAATATCAACAGAGGTAGATTCACAGAACAATGCCGATTACGCTTAAT | 48 |
| 13[77] | 10[70] | 13[77] | TAAATTCGGCTTACTCAAACGACATTCTGGCCAACACATTAAAAACAAAC | 50 |
| 3[301] | 6[308] | 3[301] | CCTCATAGACGACAAAGGCTTATCCGTCAAGATCTCTGAAAAACGTCA | 48 |
| 1[259] | 13[272] | 1[259] | AGGCGGTTAGAATACTTAATTGAGAA | 26 |
| 8[41] | 4[42] | 8[41] | GGTTAGAATAGATAATACATTTAAAATATCTATGCGCGAAGCGTAAGA | 48 |
| 6[223] | 11[216] | 6[223] | CATAGCCCTTATTTTAATAACGGAATACAATAAGTTACACGAGA | 44 |
| 35[238] | 24[231] | 35[238] | AGGATAAGATAAGAGCTGAATAAACACCCGGTGTATCATAAGGGAACCG | 49 |
| 3[322] | 6[329] | 3[322] | TAAAGTTTAAAGTATAAGAACGCGAGGGAGGTTAGCGTCACACCATTA | 48 |
| 5[189] | 1[202] | 5[189] | TCAGAGCCAGGGTTGACCACCCTCCCGCTCACTGCGCTCACTGCCCG | 47 |
| 8[118] | 12[105] | 8[118] | CTGATTATCAGATGTACCTTTTCAAAATTTTTCTGAG | 37 |
| 8[335] | 4[336] | 8[335] | AAGAAACATTATCACCGTCACCCACCAGTAGTACATGGCTAACGGGGT | 48 |
| 7[70] | 11[69] | 7[70] | TTACAAACATTGTTTGGATTATTTCAGATCAAGAAATAAC | 40 |
| 8[244] | 13[237] | 8[244] | GAAACCGAGGAAAGAAGCGTTCCAGATTTTTACTGAACAAAAGCC | 45 |
| 21[231] | 17[244] | 21[231] | AACTTTATTTAGGACCAAAAGCGGCCTCCCGCTTCTGGTGCC | 42 |
| 29[287] | 40[280] | 29[287] | CCAATACCCTCAAACGGTAATAAAGGCCGGAGACAGCCTGAGCCAACAG | 49 |
| 5[294] | 1[307] | 5[294] | GAATGGAACCCCTGCCTCAGACAGCAAGGAATTGTTCACCAGTGAGACG | 49 |
| 10[174] | 2[161] | 10[174] | AATAAGCCAAGATCGGCCTAGAGGCGAAAAACCGTAATGAGTAGCATAAA | 50 |
| 22[258] | 27[265] | 22[258] | CTTGCCCCCTTATGAGAAAGAAGTAAGAGCAACACTGATACCGGATCGT | 49 |
| 3[196] | 6[203] | 3[196] | AGAGCCATACGAGCTACCGCACTCATAAATAAGAACCACCCGTTTGCC | 48 |
| 33[252] | 30[245] | 33[252] | CCTGAGACAGGAAGCTCATTTTTTAACCAGTAACAACCCGTCGGATTCT | 49 |
| 4[230] | 8[224] | 4[230] | GCGGGGTTTTGCTCAGCGCCGCCTTTGCCACATAAAAACAGGCGCAAT | 48 |
| 6[144] | 10[126] | 6[144] | AGCAAATGAAAAATCATTATCCGGAATTATCATCGAAACCGCAGA | 45 |
| 3[280] | 6[287] | 3[280] | GCCTGTATTCAGCTAAGCAAATCAGATGCACCTAAAGCCAAGCAGCAC | 48 |
| 25[231] | 16[224] | 25[231] | CTAAAACCACTACGTTGCAGGAACAACCGCCAGTGTGGGTAACGCCAGG | 49 |
| 20[279] | 17[286] | 20[279] | CGGAACAACCCTCGTTTTGCCTCAGGCTGCGCAAC | 35 |
| 42[286] | 25[293] | 42[286] | TGCTTTACAAATATGAACTAATATACCAAGACTTTAGTACAACGGAGAT | 49 |
| 6[216] | 2[210] | 6[216] | CCTTATTAGACCAGAGCTACCAGGCGATTTTCAGGCTGTTTCC | 43 |
| 10[181] | 6[189] | 10[181] | AATCCAAAAAATGAAAATTAAGACTAACATATAAAAGAAAATAATCAA | 48 |
| 13[175] | 5[181] | 13[175] | AAAAGCCTGTTTAAACCAAAGAACCGTATAAGTATAGCCCGGTCAGAGCC | 50 |
| 10[251] | 7[244] | 10[251] | CTAACGAGGTCAGAGACTGTAGCGCGTTTTAAATTCAT | 38 |
| 21[259] | 8[252] | 21[259] | CGATTTTTCCATTAATTATACGTTTGCCTCAAAGACACGAACAAAGTTA | 49 |
| 12[97] | 6[91] | 12[97] | CTTTTTTCGCTATTAATTTTACCTGAGATAAAAACCCTCAATCAATAT | 48 |
| 3[42] | 13[55] | 3[42] | TTTGATTACCGAGTAAAGAAAGGAGCGGGCGCCGATTTATTTAGT | 45 |
| 8[125] | 4[126] | 8[125] | ATATTCTTAAAAGTTTGAGTAACTAAAGCATAACACCGCTCTGAAATG | 48 |
| 19[238] | 22[245] | 19[238] | GAATTACCAATGACGAGTTAAAGGCCGCAAAATACTGAATTATGACGAG | 49 |
| 0[307] | 4[308] | 0[307] | TGGTGGTATGTAATTTAGGCCAGACGTTAGCGTTATAAACA | 41 |
| 1[119] | 12[112] | 1[119] | TACAGGGCGGGAGCTAAACAGGAGATTACCGTCATAGG | 38 |
| 1[25] | 4[36] | 1[25] | AAAGGAAGGGGAAAGCCAACTTTTTCAAATGCAAAAATACTTCATACGTG | 50 |
| 1[140] | 3[151] | 1[140] | TTTGACGAGTGCTTTCCTCGTTAGGAAAAACGCTCATGGAAATA | 44 |
| 8[55] | 0[42] | 8[55] | TGAATAATGGAAGAAAGATACATTATCAATGCTGATATATGAGCTT | 46 |
| 6[328] | 11[321] | 6[328] | CCATTAGCAAAGGTGAAATGAAATAGCAAAGAGAGCCTTAAAGTATTC | 48 |
| 6[286] | 11[279] | 6[286] | CGTAATCAACCGATTAAGAAAAGTAAGCTGAACAAATTTTAATAGC | 46 |
| 12[300] | 2[280] | 12[300] | ATAAACCCAACTCCGAAATCGGCTTTTTCTTCGAATAATAATTTTTT | 47 |
| 1[210] | 3[230] | 1[210] | TCGGGAAACCTGTCGGGAACAATCTTACCAGTATAGAAAAAAGCCCAA | 48 |
| 8[272] | 4[273] | 8[272] | AGATAGCAAAGGGCGACATTCAGTAGCGACCAAATAAATTCTGAAA | 46 |
| 5[196] | 7[216] | 5[196] | GCCACCAACAGCCACTTTACAGAGAGCCAAAAACACCACGGAATAAGT | 48 |
| 3[119] | 9[125] | 3[119] | CCAGCCATGATTATTTACATTGGCGAGGCGGTTATTCATTTACATCGGGA | 50 |
| 5[84] | 1[97] | 5[84] | ACGAACCAAATAAAAGGTATCGGCCGAACGGTACGCGCGTAACCACCACA | 50 |
| 13[105] | 3[97] | 13[105] | GACCGTTTGGGGTCGAGGTCCCGCCGAAGGGATTTTAGACAGTTGCTGGT | 50 |
| 2[62] | 11[62] | 2[62] | ATAATCAGTGAGGCCAGTAATAAATGTAAATATATGTGAGTG | 42 |
| 24[258] | 15[265] | 24[258] | GCGCAGACCCAGCGAACGGGTTTTTGCGGATAGTTCTGCAGGTCAAGGC | 49 |
| 33[231] | 18[238] | 33[231] | ACAAAGGTATTTAAATTAAATTTTTGTTCCGTGGGGCATCGTACAGTAT | 49 |
| 27[273] | 18[266] | 27[273] | AGCAGCGTTTCTTAGGAGCCTCGCCAGCTGGCGAATCGCCATAGAGGGG | 49 |
| 8[167] | 5[174] | 8[167] | GTATGTTGAAAATACATACATAGCCACCACCCGCCACCC | 39 |
| 4[141] | 7[146] | 4[141] | CGCTCAATCGCTGCAACAAAATCGAATAACGGATTCAAGGAGATTTTGCG | 50 |
| 13[56] | 10[49] | 13[56] | TAATTTAACTATCATCACTTACCCTTCTGACCTGAAACTGATAAATTAAT | 50 |
| 18[265] | 16[245] | 18[265] | GTAATGTTTGAGGGAGGCAAAGCGCCATAGGGGGATGTGCTGCAAGGCG | 49 |
| 16[244] | 41[251] | 16[244] | ATTAAGTCCAAGCTTGCATGCGCGCCGAGAGGCATTTCATCAGCATCAA | 49 |
| 42[258] | 34[266] | 42[258] | GACCATACATCTGCCAAGTAAAATGTTTCAGAAAAGCAAACACACCATC | 49 |
| 12[272] | 22[266] | 12[272] | GAACGCGAGTACAACATGAAAGCACCCTCGGAAGTTAAGAACTTTCAGTG | 50 |
| 7[315] | 2[315] | 7[315] | ATTCATTAAGGCCGGTTTACCGAGTGCCCGAACGATCAGTGAGAA | 45 |
| 10[62] | 7[55] | 10[62] | AAACAAGCCCTAAATTGAGGAAGGTTATCTGAGGATT | 37 |
| 3[175] | 6[182] | 3[175] | CCACCCTCTCAATAAACGGGTATTAATTATCCCACCACCCAATCACCG | 48 |
| 39[231] | 29[237] | 39[231] | CTTAATTGGTCATTAAGCAAATCTTTACGATGGGC | 35 |
| 4[265] | 9[272] | 4[265] | TATTAAGACTTGATATATCTTAAACTGAACACCC | 34 |
| 6[307] | 11[300] | 6[307] | CCAATGAAATATTGATCTTACCGAAGCCAATTGCTATTTTATAG | 44 |
| 12[279] | 15[272] | 12[279] | AATGCATCGCCACTTATAAATCAAATGCGTATAAATCTCCTCCAAAA | 47 |
| 1[245] | 11[251] | 1[245] | CCAACGCATCCCCGGGTACCGAGCCATGTACTAAGTCTTTTCATCGTAG | 49 |
| 17[245] | 34[238] | 17[245] | GGAAACCGACGACGAACCGTGAATCAAAGTCATTGCCGTTCTAGCTGAT | 49 |
| 33[273] | 18[280] | 33[273] | AGAGAATTAATCAGACGCCATCAAAAATCATCAACATAGCGTAAAGAAG | 49 |
| 19[280] | 21[293] | 19[280] | TTTACCAAGGTGAAAAAGACAGCATCGGGGACTAAGTCAGGA | 42 |
| 6[258] | 1[258] | 6[258] | TTAGCGTCACGATTGGCGGCTGAGACCTGAGTTCTCTAGAGGGCGGGGAG | 50 |
| 39[245] | 34[252] | 39[245] | ATAATGCATTTCGCAGGTGGCATCAATTAATCGGTTAGAACCTATTCAA | 49 |
| 2[265] | 5[272] | 2[265] | AAAAAAGATCGTCACCCCTGTTATTACCGCGCCCATCCTGATCACAAA | 48 |
| 0[97] | 4[91] | 0[97] | GCCGTAGTTTGAAATACCAGACTACAATATCCCAGTCACACGACCAGT | 48 |
| 12[34] | 6[35] | 12[34] | TCCAATCAACAGTACATAATAACAAAGTCTTTATTAGGAGC | 41 |
| 21[238] | 23[258] | 21[238] | ATCATTGGTAATGCACTCATCTTTGACCCGGTCAACAGACCAGGCGCAT | 49 |
| 4[69] | 13[76] | 4[69] | GAGATAGAGCCTGAGGTGTTTTTTGGCAAGTGTAGCGGGAACCCCTGACC | 50 |
| 12[76] | 6[70] | 12[76] | GGTTGGCTTGCTTCTGTATGATGAAATACCGATGGCAAATCAACAGTT | 48 |
| 4[174] | 0[175] | 4[174] | AATAGGTGTCAGAACCGCGAGCCGGAGAGCTAACTCACATTATCAAAG | 48 |
| 12[195] | 2[175] | 12[195] | ATGTAGAGTATCGTGGACTCCAACGATTGCGTAATTCCACACAACATA | 48 |
| 3[329] | 9[335] | 3[329] | TGTCGTCCAGTGCCTTGAGTAACTTCCAGTATTGAAGATAACCCACAAG | 49 |
| 0[202] | 4[196] | 0[202] | AAAGAACATATGCGTTATACTAATTCCACCCTCGATAAGTGCCGTCGAG | 49 |
| 4[307] | 7[314] | 4[307] | GTTAATGCAGCGCAGTTAGTTGAGCGCTAATATCTAGCTACGGAAATT | 48 |
| 13[140] | 5[145] | 13[140] | GAATAAAGAGTCAATAGTGAACATAGCGATAAGTTACAGTGCCA | 44 |
| 7[91] | 12[84] | 7[91] | ATTAAATCATTCATCAATATATGAAAGAAGAAATCGAACCTC | 42 |
| 12[223] | 6[224] | 12[223] | ATAATATACAAGCAAGCCGGCCTAAAGCATTGATTTTCGGT | 41 |
| 1[315] | 12[329] | 1[315] | CTGATTGCCCTTCACAGGCGACATTTTCGAGCCAAAAGGT | 40 |
| 28[272] | 36[266] | 28[272] | AACAGCTTATCATAACATTATAGCCCGATTAGAGAATTTTAAAGAGCAT | 49 |
| 6[272] | 21[279] | 6[272] | AGAATCAACAAGCGCTTAGCCGCTGACCTTCATCAACTGCTCAGGCTCAT | 50 |
| 34[265] | 24[259] | 34[265] | AATATGACTCATATGTACCTTCAACATGAATAAGGAGGCTGGGAACGAG | 49 |
| 6[181] | 9[174] | 6[181] | GAACCAGAAAGGTGGCCCTTATTACGCAACGTC | 33 |
| 7[224] | 3[237] | 7[224] | GTCACAATCAATAGACATCGGCACAGGAGGTAGGATTATAGGAAC | 45 |
| 12[342] | 2[322] | 12[342] | ACCGACAGTAAGGTTTGCCCCAGCCGCCTGGTCAACAGTTTCAGCGG | 47 |
| 12[69] | 1[62] | 12[69] | GTTATATCATCTTTAAAGGGAGCCCCTAGGGCGC | 34 |
| 9[49] | 5[69] | 9[49] | AATTGCGTAGATTACTTCTAGAAGTATTAGACTGAAAGGAAACATCGC | 48 |
| 13[84] | 3[76] | 13[84] | TAATGAAGCACTAAATCGTCACGCTGCCAGAATCCTGAGAATAGAAGAA | 49 |
| 31[245] | 20[238] | 31[245] | AAATCAGATTGTATAAGCAAACTATCAGAATCAGGGCGGATTGTTGAGA | 49 |
| 3[126] | 13[139] | 3[126] | TGCAACAGAATCAGAGCGCGTACTATGGTTGCGTGAACCAAATAA | 45 |
| 9[252] | 0[245] | 9[252] | GAATTCCAACGGAATCTATCAATAGGGGCCCGAGATAGGG | 40 |
| 12[321] | 2[301] | 12[321] | ATTCTGTCAGAGGAAATCCTGTTTGAGGCAACAGTAGAAAGGAACAACTA | 50 |
| 39[266] | 34[273] | 39[266] | TTTTAAATTTAGTTTAGTAGTAGCATTAAAGCCTCATGCAATGTCAAAT | 49 |
| 31[266] | 20[259] | 31[266] | AATAGGAAAAAGCCCCAAAAAGTCTGGACGAGAATAAGAGGATACAGGT | 49 |
| 6[202] | 11[195] | 6[202] | ATCTTTTCCGCAAAGGAACTGGCATGATAGCAGCTATTATACCAAG | 46 |
| 39[273] | 30[266] | 39[273] | TATGCAAGTCAGGAAAGACTTAACAGTTAGACTGGATTAAATGTGAGCG | 49 |
| 11[126] | 0[119] | 11[126] | TTGAAAATTTAGCGTTATCACCCAAATC | 28 |
| 9[70] | 6[84] | 9[70] | GTTTAACGTCAGAATCCTGAATTCGACAACTCGTCTGGTCAGT | 43 |
| 17[224] | 41[230] | pass17[224] | TCCGGCAAGGAAGATGGTGTACCTGACTATTTTTTTTTTTTATAGTCAG | 49 |
| 30[237] | 32[209] | pass30[237] | AACAAACGGCGGATAATTCGCATTGTAAACGTTAATATTTTTT | 43 |
| 25[207] | 24[214] | pass25[207] | TTTTTAAAGAGGCAAAAGAATACAAACTGACCAACTTTTTT | 41 |
| 31[207] | 30[207] | pass31[207] | TTTTTTTTTTTTGTTAATGACCGTAATGGTTTTT | 34 |
| 42[314] | 41[314] | pass42[314] | TTTTTATATTCATTAGCTTCAAATTTTT | 28 |
| 34[311] | 33[311] | pass34[311] | TTTTTTCAAAAGGGTGAGCGTAAAACTAGCTTTTTT | 36 |
| 40[230] | 38[213] | pass40[230] | TTTGCGGATTTTTTTTTTTGGCTTAGAGCTGTTTAGCTATTTTTTT | 46 |
| 37[213] | 34[231] | pass37[213] | TTTTTTATTTTCATTTGGGGCGCGATATGACCCAACGCAAAATTAA | 46 |
| 33[210] | 34[210] | pass33[210] | TTTTTTATTTTTGAGAGATCTTGCCGGAGAGGGTAGCTTTTT | 42 |
| 22[311] | 21[311] | pass22[311] | TTTTTTTCATTACCCAAACGTTGGGAAGAATTTTTT | 36 |
| 28[303] | 40[287] | pass28[303] | TTTTTAGCTTGCTTTCGGACGACGATAAAACCGCGTTTGCAAACT | 45 |
| 25[301] | 25[300] | pass25[301] | ATCGCCTGATTTTTTTTTTTAAATTGTGTCGAAATTTGTATC | 42 |
| 22[223] | 26[207] | pass22[223] | ATTGGGCTTTTTTTTTTTTGAGATGGTTCAACCTAAAACGTTTTT | 45 |
| 32[309] | 31[311] | pass32[309] | TTTTATGTCAATCATAGTCTGGCCTTTTTTTTTT | 34 |
| 41[294] | 29[315] | pass41[294] | TAATTCGGAATCCCTGCGGAATCGTCATAATTTTTT | 36 |
| 15[206] | 28[224] | pass15[206] | TTTTTTACGACGTTGTAAAACGACGATCGCCC | 32 |
| 18[311] | 17[308] | pass18[311] | TTTTTTATAGCGAGAGGCAAGGGCGATTTTTTT | 33 |
| 35[210] | 36[211] | pass35[210] | TTTTTGGAGAAGCCTTTATTTCTGTAATACTTTTGCGTTTT | 41 |
| 20[311] | 19[311] | pass20[311] | TTTTTTAAATCTACGTTAATAAAAACCAAATTTTTT | 36 |
| 17[209] | 16[206] | pass17[209] | TTTTTTAGCCAGCTTGTTTTCCCAGTCTTTTTT | 33 |
| 30[307] | 33[286] | pass30[307] | TTTTTTCCTGTAGCCAGCTTTAATTCGCTGTACCCCGGTTGACGATGAA | 49 |
| 16[307] | 18[287] | pass16[307] | TTTTTCGGTGCGGGCCTCTTCTGTTGGGTTTTGCA | 35 |
| 24[293] | 23[311] | pass24[293] | CCGCGACCTTGACAAGAACCGGATATTTTTTT | 32 |
| 38[315] | 40[294] | pass38[315] | TTTTTTATATAACAGTTGATTCCTGGAAGACCGGAA | 36 |
| 29[207] | 18[211] | pass29[207] | TTTTTGATAGGTCACGTTCGCACTCCTTTT | 30 |
| 16[286] | 15[303] | pass16[286] | GCTATTATTAATTGTATCGGTTTATCTTTTT | 31 |
| 36[311] | 35[311] | pass36[311] | TTTTTTCAAAGAATTAGCTAGGTAAAGATTTTTTTT | 36 |
| 19[224] | 20[210] | pass19[224] | CATAACGATACCACATTCAACTATTTTT | 28 |
| 22[293] | 37[311] | pass22[293] | TCAACGTACGGTGTCCAATTCATAAATCATACAGGCAAGGTTTTTT | 46 |
| 40[314] | 39[314] | pass40[314] | TTTTTGCGAACCAGTTTCATTCCTTTTT | 28 |
| 26[300] | 26[301] | pass26[300] | GCTTTGAAACGAGGGTAGCAACGTTTTTTTTTTGCTACAGAG | 42 |
| 23[213] | 22[231] | pass23[213] | TTTTTTTTGAAAGAGGACAGATGAAAGAACGA | 32 |
| 27[207] | 22[224] | pass27[207] | TTTTTTATATTCGGTCGCTGAGGCAAGGCACTAATTTCGTAGTAA | 45 |
| 19[211] | 28[207] | pass19[211] | TTTTATGCAGATAACGCATAACCGATTTTT | 30 |
| 3[273] | 13[286] | ring_verb3[273] | ACTACAACCACGTTGATGGGCGCCAGGGTGGAAAATCCTATTTATTTTTTTTATACCGTATTCCGAGGCG | 70 |
| 4[286] | 9[286] | ring_verb4[286] | ACCTATTATCCTCATCAGCTACAAGTCATTTTTTTTACGGCCTCGAATAGTAGA | 54 |
| 2[237] | 13[244] | ring_verb2[237] | CTCGAATTGCATTAATGAATCGGTTGAGTAACGCTTTTTTTTTAATCGCTATGGTGTGGTT | 61 |
| 4[244] | 9[244] | ring_verb4[244] | AGAAGGATTTGAGGCAGCGTCTCATTAGTTTTTTTTACCAGGGCAGTTTTATCC | 54 |
| 9[245] | 7[258] | ring_verb9[245] | GTTCACATTAGTCGGCAATTTTTTTTACGGGACCAGAAGATGGTTTACCAGCGC | 54 |
| 9[287] | 6[294] | ring_verb9[287] | TGGTATTCGCAATGGTCTTTTTTTTTGAGGGTCTTTTTGAGGGAGGGAAGGTAAACCATCGAT | 63 |
| 13[287] | 4[287] | ring_verb13[287] | ATATAGCGGCTAGGAACGTTTTTTTTACAACGAACATGGCATTCCAATTTCGGA | 54 |
| 13[245] | 4[245] | ring_verb13[245] | GGTTTCCTAGCAGTTTATTTTTTTTTCAACAGCAATAGACGTAACATCCTCAAG | 54 |
| 3[152] | 4[142] | Mitte3[152] | TTTAGTACCGCCACCCTATCACCGTACTCAGGAGGCCTACATTTTGA | 47 |
| 5[146] | 6[145] | Mitte5[146] | CGCTGAGCCGCCACCCTCAGAACGGAACCGCCTCCCTCAGAGAGCCAGC | 49 |
| 10[167] | 11[167] | Mitte10[167] | AAACGATTTTTTGAATACCAGCTTAGATTATTATCATT | 38 |
| 12[167] | 0[140] | Mitte12[167] | TGTCTTTCCAGACGCTGAGAACACCGGATGGCCCACTAC | 39 |
| 7[147] | 9[167] | Mitte7[147] | GAACAAAGAAACGTAAGCAAACCACCAGGCCTGATTGCTTTGTTTA | 46 |
| 2[160] | 13[167] | Mitte2[160] | GTGTAAAGCCTATAACGCACGTGGGGTGCCTCTATCAGAATCATAATTA | 49 |
| 7[343] | 8[28] | verschl7[343] | GACTTGAGCCATTTGGGTTTTTTGATTAGAGCCGTCAACCTAC | 43 |
| 9[343] | 10[343] | verschl9[343] | AGTTAAGCTTTTTTTATTTGCACGTGAATTACCTTTTTTTTCCTCCCGA | 49 |
| 5[349] | 9[41] | verschl5[349] | GATACAGGAGTTTTTTTTGAATGGCTATTTTTCATTTAAAACAGAAAT | 48 |
| 4[35] | 5[348] | verschl4[35] | GCACAGACAATATTTTTTTTTTGTACTGGTAATAAGTTTTTTTGAT | 46 |
| 6[34] | 6[343] | verschl6[34] | ACTAACAACTAATATTTTTTAATTAGAGCCAGCAAAAT | 38 |
| 0[27] | 13[27] | verschl0[27] | GGCGAACGTTTTTTCGGTCCACGCTTAAGAGAATTTTTTAACGCGAGAA | 49 |
| 0[41] | 2[352] | verschl0[41] | GACGGGAAGAAAGCAGAGTCTGTCCATCACGCAAATTTTTTTTCTGTATG | 50 |
| 2[351] | 3[34] | verschl2[351] | GGATTTTGCTCGTTAGTAAATGAATTTTTTTTTTAACCGTTGTAGC | 46 |
| 8[27] | 11[342] | verschl8[27] | CATATCAAAATTTTTTTCCAATAATAAGAGCAATTGCTTGCGGCGTT | 47 |
| 12[27] | 11[27] | verschl12[27] | GCAAGACAAAGTTTTTTTATAAAGTTTAGCGAATTTTTTTTTTAATGGA | 49 |
| 3[336] | 1[24] | verschl3[336] | TTTCCAGAAAACAACTTCCCTGAGAGAGTTGCAGCAAGTTTTTTGGCGAG | 50 |
